# Stem cell graft dose and composition could impact on the expansion of donor-derived clones after allogeneic hematopoietic stem cell transplantation – a virtual clinical trial

**DOI:** 10.3389/fimmu.2024.1321336

**Published:** 2024-12-16

**Authors:** Thomas Stiehl

**Affiliations:** ^1^ Aachen Medical School, Institute for Computational Biomedicine & Disease Modeling, RWTH Aachen University, Aachen, Germany; ^2^ Department for Science and Environment, Roskilde University, Roskilde, Denmark

**Keywords:** allogeneic hematopoietic stem cell transplantation (AlloHCT), clonal hematopoiesis of indeterminate potential (CHIP), chronic inflammation, clonal dynamics, hematopoietic stem cell (HSC), mechanistic computational model, virtual clinical trial, mathematical oncology

## Abstract

**Introduction:**

Hematopoietic stem cell transplantation is a potentially curative intervention for a broad range of diseases. However, there is evidence that malignant or pre-malignant clones contained in the transplant can expand in the recipient and trigger donor-derived malignancies. This observation has gained much attention in the context of clonal hematopoiesis, a medical condition where significant amounts of healthy blood cells are derived from a small number of hematopoietic stem cell clones. In many cases the dominating clones carry mutations conferring a growth advantage and thus could undergo malignant transformation in the recipient. Since clonal hematopoiesis exists in a significant proportion of potential stem cell donors, a more detailed understanding of its role for stem cell transplantation is required.

**Methods:**

We propose mechanistic computational models and perform virtual clinical trials to investigate clonal dynamics during and after allogenic hematopoietic stem cell transplantation. Different mechanisms of clonal expansion are considered, including mutation-related changes of stem cell proliferation and self-renewal, aberrant response of mutated cells to systemic signals, and self-sustaining chronic inflammation triggered by the mutated cells.

**Results:**

Model simulations suggest that an aberrant response of mutated cells to systemic signals is sufficient to explain the frequently observed quick expansion of the mutated clone shortly after transplantation which is followed by a stabilization of the mutated cell number at a constant value. In contrary, a mutation-related increase of self-renewal or self-sustaining chronic inflammation lead to ongoing clonal expansion. Our virtual clinical trials suggest that a low number of transplanted stem cells per kg of body weight increases the transplantation-related expansion of donor-derived clones, whereas the transplanted progenitor dose or growth factor support after transplantation have no impact on clonal dynamics. Furthermore, in our simulations the change of the donors’ variant allele frequencies in the year before stem cell donation is associated with the expansion of donor-derived clones in the recipient.

**Discussion:**

This in silico study provides insights in the mechanisms leading to clonal expansion and identifies questions that could be addressed in future clinical trials.

## Introduction

Hematopoietic stem cell transplantation (HSCT) is a widely used intervention to cure malignant and non-malignant diseases (1–3). A rare but severe complication is donor-derived malignancy, where (pre-)malignant donor clones engraft in the recipient and progress into overt hematological cancers (4). This topic has gained much attention in the context of clonal hematopoiesis of indeterminate potential (CHIP). CHIP is defined as a state where a significant amount of an individual’s mature blood cells are derived from the same hematopoietic stem cell (HSC) clone. The respective clone is characterized by one or multiple mutations which presumably result in a growth advantage. Usually a variant allele frequency (VAF) of 2% and above is used to define an individual as CHIP positive (5).

Statistically CHIP is closely intertwined with systemic chronic inflammation as demonstrated by its association with inflammation-related conditions such as atherosclerosis, smoking and autoimmunity (5–9). There is evidence from animal models that chronic inflammation supports the expansion of the mutated clones (10–12). In addition to this, mutated cells can exhibit high expression of pro-inflammatory mediators and thus increase the systemic inflammatory burden. This self-enhancing loop might not only aggravate inflammatory comorbidities, it could also trigger the evolution of mutated clones towards malignancy (5, 13).

The prevalence of CHIP is approximately 1% in individuals below 50 years of age and above 10% in individuals aged 65 or older (14). With sensitive methods clones carrying CHIP-related mutations can be detected in 95% of individuals older than 50 (15). Therefore, clonal hematopoiesis affects a significant proportion of persons eligible for donation of hematopoietic stem and progenitor cells (HSPCs). The role of CHIP for allogeneic HSCT is so far not well understood. Complications that could arise in the case of donor CHIP include: (I) Donor clones harboring a growth advantage expand in the recipient, accumulate mutations and transform into donor-derived leukemia (16, 17). (II) Donor clones give rise to pro-inflammatory cells which trigger chronic inflammation and increase the recipient’s comorbidity burden. The latter fear is grounded in the observations that CHIP is associated with high all cause mortality and that CHIP mutations may have adverse effects on the progression of conditions such as chronic ischemic heart failure or chronic kidney disease (18–20).

Multiple clinical trials have been performed to shed light on these potential complications. For autologous HSCT in multiple myeloma CHIP seems to be associated with adverse outcomes (21). For the allogenic setting some trials report no impact of donor CHIP on recipient survival, disease progression and transplantation-related complications (22), whereas other studies report e.g., higher incidences of acute or chronic graft-versus-host disease in the presence of donor CHIP (23–25). Data on young recipients and transplantation of unrelated donor grafts is still relatively rare and subgroup effects potentially exist (23, 25, 26). Therefore, it remains an open question whether donors should be screened for CHIP and excluded from stem cell donation if CHIP is detected (27, 28). Since the latter might considerably reduce the access to matching donors, a careful risk-assessment based on patho-physiological mechanisms is required before recommendations can be given.

Recently, longitudinal data on the dynamics of donor CHIP clones after allogeneic transplantation have become available (23, 29). They reveal a broad spectrum of clonal dynamics ranging from moderate temporary expansion in the months post transplantation to persistent growth over multiple years. Since the mechanisms leading to the observed heterogeneity are unknown it is challenging to predict how donor-derived CHIP clones will evolve in the recipient.

In this work we will use computational models to provide insights in the patho-mechanisms leading to the expansion of donor clones after allogeneic HSCT. Mathematical modeling has significantly contributed to the understanding of the hematopoietic system and its diseases (30–36). The feedback mechanisms governing hematopoietic reconstitution after HSCT have been studied e.g., in (37–41). The role of feedback mechanisms in the progression and treatment of hematopoietic malignancies has been considered in (42–45). The impact of stem and progenitor cell kinetics on clonal dynamics or progression of hematologic malignancies has been modeled in (46–58, 33). Important previous modeling works on clonal hematopoiesis cover the role of chronic infection as a driver of clonal hematopoiesis (11) or the interrelation of atherosclerosis, stem cell proliferation and clonal hematopoiesis (59). Park et al. (60) have proposed an ordinary differential equation model accounting for stem cells, mature lymphoid and mature myeloid cells. The model considers two clones (wildtype and mutated). They use the model to simulate the impact of clonal competition and differences of clonal growth rates on hematopoietic productivity and clonal frequency after autologous stem cell transplantation. They apply their model to mouse data to estimate the intensity of clonal competition.

Novel human data which has recently become available (23, 29) paves the way for the development of new quantitative models with potential clinical applications. Such models not only give us the opportunity to systematically simulate how transplantation procedures impact on the dynamics of donor-derived clones, they also advance our understanding of the patho-physiological mechanisms shaping the tremendous inter-individual heterogeneity of clonal trajectories.

Notably, the risk and severity of important CHIP-related complications depend on the abundance of the mutated cells, quantified by their VAF. The more mutated cells exist, the higher the probability that at least one of them acquires sufficient additional mutations to undergo malignant transformation. Similarly, the more pro-inflammatory donor-derived cells are present, the higher is the recipient’s risk to develop inflammation-related CHIP comorbidities such as atherosclerosis. For this reason, it is important to understand which mechanisms trigger the expansion of donor-derived clones in the recipient, whether the degree of expansion can be predicted and whether there exist straightforward means to reduce it.

In this work we propose mechanistic computational models of clonal dynamics after allogeneic hematopoietic stem cell transplantation. The models account for important drivers of clonal expansion such as mutation-induced alterations of kinetic cell properties, aberrant response of CHIP clones to systemic cytokines, and mutation-driven inflammatory processes. To account for the inter-individual heterogeneity among donors and recipients we perform *in silico* clinical trials to study the impacts of the transplantation procedure on clonal dynamics. We focus on the following questions:

- Which mechanisms can explain the different patterns of clonal expansion after transplantation?
- How does the interplay of chronic inflammation and CHIP impact on the clonal dynamics in the host?
- What is the impact of the number of transplanted cells on the VAF dynamics after transplantation?
- What is the impact of growth factor support such as pegfilgrastim on the VAF dynamics after transplantation?
- Which quantities could be used to predict the expansion of donor-derived clones in the recipient?

The results shed light on the mechanisms underlying clonal expansion and lead to new testable hypotheses which might inspire clinical trials.

In the next section, we derive the mechanistic models of engraftment after allogeneic HSCT. We perform model simulations to investigate which patho-physiological mechanisms can trigger the expansion of donor-derived clones. After that we compare model dynamics to patient data from literature and perform *in silico* clinical trials to investigate how the dose of transplanted cells and growth factor support impact on the expansion of CHIP clones. We also run exemplary simulations to better understand the role of chronic inflammation for the expansion of donor-derived clones.

## Materials and methods

### Mechanistic ordinary differential equation model of white blood cell formation

We aim to study the dynamics of the variant allele frequency (VAF) after allogenic hematopoietic stem cell transplantation (HSCT). The quantification of the VAF in peripheral blood (PB) is based on nuclear DNA which is contained in white blood cells. The largest fraction of white blood cells are neutrophil granulocytes (61). Therefore, we derive a mechanistic model of neutrophil formation after HSCT. Due to the high amount of cells forming the hematopoietic system, we use ordinary differential equations (62).

**Figure 1:**
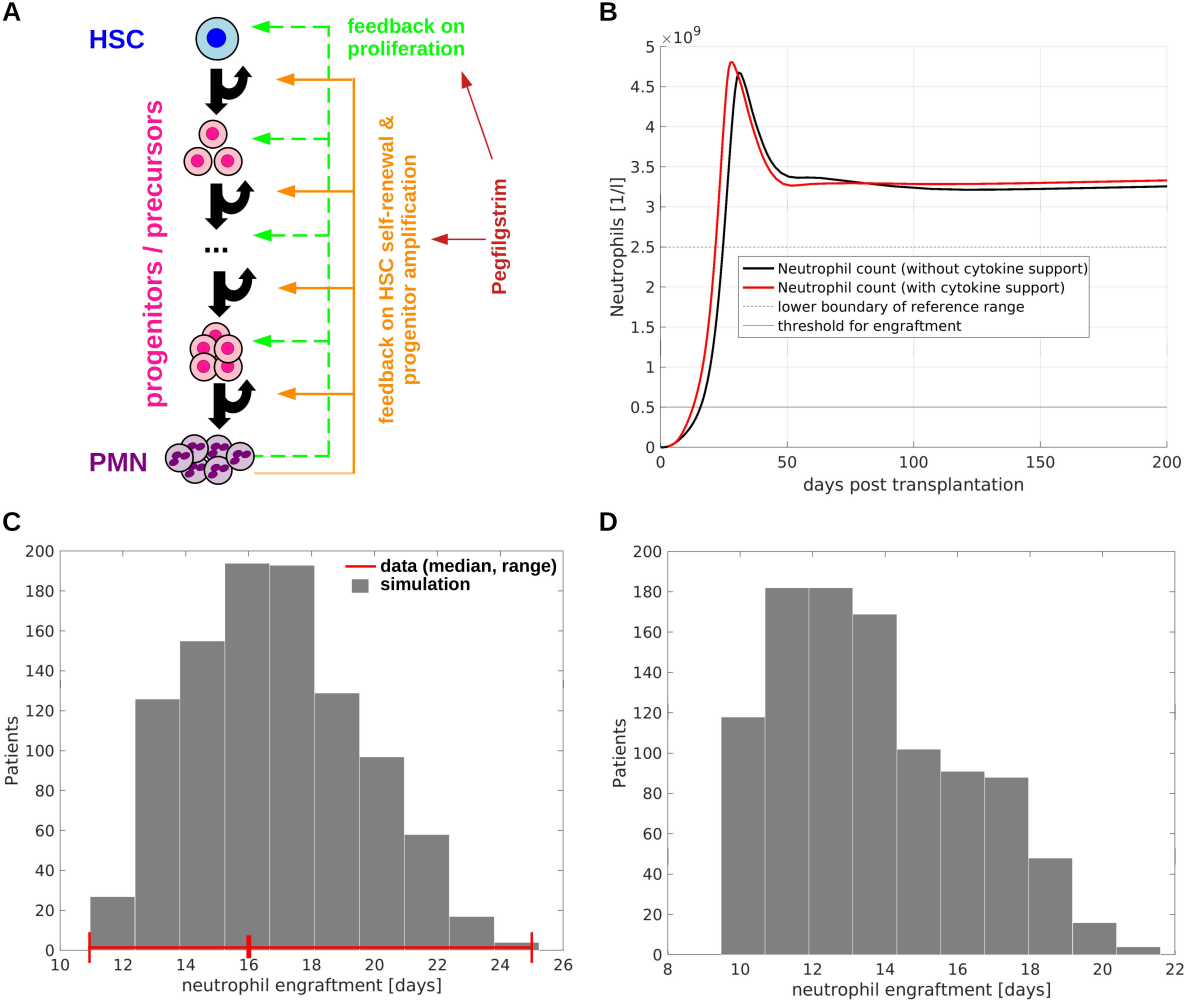
Model of allogeneic hematopoietic stem cell transplantation. **(A)** Overview of model structure and feedback regulations. The model accounts for HSCs, multiple stages of progenitors/precursors and mature neutrophils (polymorphnuclear neutrophils, PMN). If there is a shortage of mature cells, HSC self-renewal, immature cell proliferation as well as the number of divisions immature cells perform before terminal differentiation increase. Exogenous cytokines such as pegfilgrastim have the same effects as endogenous feedback signals. **(B)** Example simulation of neutrophil engraftment with and without growth factor support. At time zero 4.7x10^6^ CD34+ cells per kg of body weight are transplanted. The time to neutrophil engraftment (time to reach 5x10^8^ neutrophils per liter of blood) is in line with observations from clinical trials (79). **(C)** Neutrophil engraftment after simulated allogeneic HSCT in a cohort of 1000 virtual patients. Each patient is characterized by an individual set of model parameters which was randomly generated. The number of transplanted CD34+ is 4.7x10^6^ per kg of body weight in analogy to (79). The red lines indicate the median and the range of the time to neutrophil engraftment observed in the trial from (79). The deviations between data and simulation are less than one day, which is acceptable since in clinics engraftment is measured in full days. **(D)** Neutrophil engraftment after simulated allogeneic HSCT with pegfilgrastim support (one dose at day 3). The transplantations are simulated for the same virtual patient cohort as shown in **(C)**. The reduction of the median time to neutrophil engraftment by approx. 3 days is in line with clinical observations (81).

The model accounts for hematopoietic stem cells (HSCs), hematopoietic progenitor/precursor cells (HPCs) and mature polymorphnuclear neutrophils (PMN). It is visualized in **Figure 1A**. Details are provided in **Section 1.1-1.7 of the Supplementary** and **Supplementary Figure 1A**. We consider the following processes: (i) cell proliferation (division), (ii) self-renewal: daughter cells originating from division belong to the same cell type as their parent cell, e.g., stem cells give rise to stem cells, (iii) differentiation: daughter cells originating from division adopt a more mature phenotype compared to their parent cell, e.g., stem cells give rise to progenitor cells, precursor cells give rise to mature cells. In the model only HSCs possess the ability of indefinite self-renewal (63). Progenitors and precursors are assumed to terminally differentiate after a finite number of divisions. Self-renewal and differentiation are quantified using the self-renewal probability (fraction of self-renewal) (40, 64), which is defined as the probability that a daughter cell arising from division adopts the same fate as its parent cell.

To account for gradual changes of cell properties during differentiation the model comprises 15 immature cell compartments between the HSC and the mature cell state. Other choices for the number of compartments lead to similar results. We consider multiple non-linear feedback loops governing HSC and HPC kinetics. They are inspired by the biological function of hematopoietic cytokines such as G-CSF. The feedback signals regulate the HSC & HPC proliferation rates, the HSC self-renewal probability and the number of divisions progenitors perform before terminal differentiation (38, 40, 41). The feedback signals are given by first-order Hill-functions of the mature cell counts. This approach is widely used and leads to realistic dynamics (38, 40, 41, 43–45, 47, 48, 53, 56). The Hill functions can be derived rigorously using time scale separation techniques (65).

In addition, we consider two modified versions of the above-described model which account for the HSC micro-environments, the so-called stem cell niches. It is known from various studies that HSCs respond to both systemic signals such as G-CSF or IFN (66–68) and to micro-environmental cues (69). Potentially, the niche is also required to mediate the response of HSCs to systemic feedbacks. A simple way to model local signals emerging from the niche is to introduce feedback loops which depend on the HSC count, whereas systemic signals are modeled by functions depending on mature cells (70). In the first modified model (modification 1), HSC self-renewal is assumed to depend on the niche, i.e., on the number of HSCs, whereas HSC proliferation and progenitor properties are subjected to systemic feedbacks, i.e., depend on the mature cell counts. In the second modified model (modification 2), HSC proliferation and self-renewal depend on the HSC counts, but the progenitor properties depend on the mature cells. Since HSCs loose their self-renewal potential in cell culture (71), we assume in both modified models that HSC self-renewal is regulated by the niche. HSC proliferation can change in response to systemic signals (72), but also depends on the niche (73). Therefore, we consider one version of the model where HSC proliferation is regulated by the niche (modification 2) and one version where it is a function of systemic signals (modification 1). Details are provided in **Section 1.4 of the Supplementary**. As we will see later, all versions of the model show the same qualitative dynamics.

### Model parametrization

The proposed model is parameterized based on data from literature. For details see **Section 2.1-2.4 of the Supplementary**. In total 100000 HSCs are assumed to contribute to neutrophil formation (74). The physiological neutrophil count is set to 5 x 10^9^ cells per liter of blood (61), corresponding to 3.1 x 10^8^ neutrophils per kg of body weight for a total blood volume of 5 l and a body weight of 80 kg. The neutrophil half-life in blood stream is assumed to be between 7 and 8 hours, corresponding to a clearance rate of 2.3 per day (61).

In agreement with literature, we assume that HSCs divide rarely (35, 75). The HSC proliferation rate under homeostatic conditions is set to 2/year (35, 75). According to experimental estimates the maximal cell division frequency among neutrophil precursors is approx. 1/day (76). To mimic a gradual change of cell properties during differentiation, we assume that the division rate of immature cells increases by the same amount from one compartment to the next with a minimum of 2/year (HSCs) and a maximum of 1/day (last mitotic immature cell stage). In agreement with experiments, immature cells can increase their proliferation rates approx. 4 fold if they are stimulated by cytokines (77). We use this information to calibrate the feedback loop acting on proliferation. Furthermore, the number of immature cell divisions before terminal differentiation increases in presence of cytokines (78). This is accounted for by a feedback loop acting on the self-renewal probabilities of HPCs. This feedback loop is calibrated based on the recovery of neutrophil counts after HSCT. The calibrated model shows a realistic time to neutrophil engraftment between 10 and 20 days (79), **Figure 1**.

### Simulation of HSCT

To simulate HSCT we set the numbers of transplanted HSCs and HPCs per kg of body weight as initial condition of the ordinary differential equation model. The amounts of transplanted HSCs and HPCs are obtained from literature. Details are given in **Section 2.2 of the Supplementary**. We only consider CD34+ hematopoietic stem and progenitor cell grafts which have been collected from peripheral blood. We assume that approximately 50% of the transplanted cells successfully home to the marrow and contribute to blood cell formation. For different percentages we obtain very similar model dynamics. We neglect the time required by the cells to migrate from blood stream to bone marrow, since this is less than one day (80). Furthermore, we neglect the donor-derived neutrophils which are present at the time of transplantation since they are rapidly cleared (41).

### Model of cytokine support after transplantation

To speed up neutrophil engraftment, G-CSF or its analogues can be administered after HSCT (81, 82). We refer to this as *growth factor support* or *cytokine support*. We consider a submodel of cytokine support which is motivated by pegfilgrastim kinetics. The drug is assumed to be injected into a subcutaneous compartment and enters blood stream at a rate which is proportional to the concentration in the subcutaneous compartment. For simplicity, we assume that elimination from the blood stream is proportional to the plasma concentration. We choose parameters such that the maximum plasma concentration is reached approx. 2 days after injection which is in the reported range of 16 to 120 hours (83). The clearance is chosen such that the half-maximal concentration is measured 3-4 days after the maximum plasma concentration is reached. This is in line with observations in individuals after chemotherapy (84). Details are provided in **Section 1.3 of the Supplementary** and in **Supplementary Figure 3**. In the model exogenous cytokine analogues such as pegfilgrastim have the same qualitative biological effects as endogenous growth factors, namely increase of the cell division rates, stem cell self-renewal and the number of progenitor/precursor cell divisions before terminal differentiation.

### Model of clonal hematopoiesis

**Figure 2:**
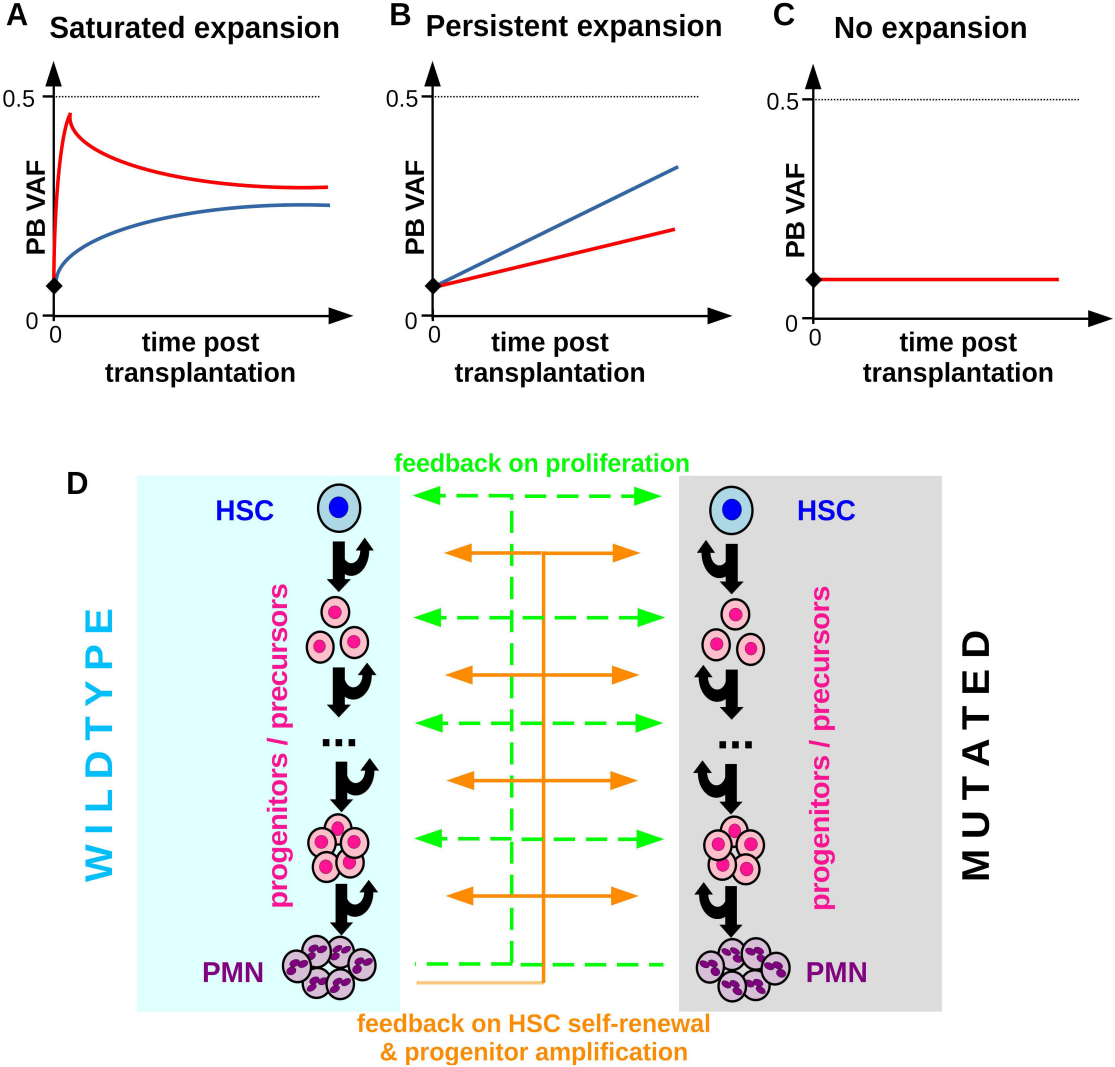
Patterns of clonal expansion after allogenic HSCT. **(A)** Saturated expansion: The VAF expansion is maximal shortly after the transplantation and slows down thereafter until the VAF eventually stabilizes at a constant value which is below 0.5 in case of heterozygous mutations. The figure depicts hand-drawn idealized examples. Different colors correspond to different individuals, the black diamond denotes the VAF in the donor’s PB. **(B)** Persistent expansion: The VAF expands over multiple years at an approximately constant rate. **(C)** No expansion: The VAF in the recipient is constant over time and similar to the donor VAF. **(D)** Overview of the extended model accounting for one CHIP clone and wildtype hematopoiesis. Mutated and wildtype cells are subjected to the same feedback loops. Compared to wildtype cells mutated cells have different parameters (self-renewal probabilities, proliferation rates, clearance rate) and can respond differently to feedback signals.

We extend the model of neutrophil formation to account for clonal hematopoiesis, **Supplementary Figure 1B**; **Figure 2**. Our main question is how donor-derived clones expand in the recipient after allogeneic HSCT. Therefore, we do not model the acquisition of new mutations but study expansion dynamics of existing clones. We assume that the mutated cells adopt the same compartmental organization as wildtype cells. In the model the effects of mutations are represented as modifications of cell parameters (proliferation rates, fractions of self-renewal, clearance rates of mature cells). Consequently, the mutated cell parameters can be different from the respective parameters of wildtype cells. In addition to that, we consider the scenario that mutated cells can respond more sensitively to perturbations of the feedback signals compared to their wildtype counterparts. Details are provided in the Supplementary (**Section 1.2.2 of Supplementary**, **Supplementary Figure 2**). For simplicity, we focus on scenarios with a single mutated clone. The model can be straightforwardly extended to account for multiple clones, however, most longitudinal observations available in literature are from patients harboring a single clone. In **Section 1.7 of the Supplementary** we consider a version of the model accounting for two mutated clones.

### CHIP-driven inflammation

There is evidence that mutated cells increase the systemic inflammatory burden due to the high expression of inflammatory mediators (5, 13). The systemic inflammatory burden is modeled by a separate ordinary differential equation with a source term which is proportional to the concentration of mutated mature cells. We assume that the inflammatory mediators have a short half-life, as most cytokines, and are degraded in a cell independent manner. Based on these assumptions we use a quasi-steady state approximation to obtain the amount of inflammatory mediators as a function of mutated mature cells. We assume that the impact of the inflammatory mediators on mutated cells saturates for high mediator concentrations. This is achieved using a first order Hill function. We consider one version of the model, where chronic inflammation triggers the expansion of mutated cells (10–12) and one version of the model where chronic inflammation, exhausts wildtype cells (85). Details are provided in **Sections 1.5** and **1.6 of the Supplementary**.

### *In silico* trials

To account for differences between individual patients we perform *in silico* clinical trials (virtual clinical trials). Each virtual patient is characterized by an individual set of parameters which is obtained by random perturbation of a reference parameter set. The perturbations are chosen from uniform distributions such that the virtual patient cohort recapitulates the variations observed in clinical trials. An important quantity after allogeneic HSCT is the time to neutrophil engraftment. Therefore, we design our virtual patient cohort (n=1000) to match the variation of the times to neutrophil engraftment observed in clinical trials, see **Figure 1**.

### Simulations

Simulations are performed using the ordinary differential equation solver *ode23s* from Matlab R2021b which is suitable for stiff systems. Random numbers are generated using the function *rand*. Parameters used for the simulations shown in the Figures are listed in **Section 3 of the Supplementary**.

### Data of clonal hematopoiesis

We relate the model dynamics to longitudinal VAF measurements taken from literature (23, 29). We solely consider heterozygous mutations. VAF at time *t* is calculated as *c_3_^M^(t)/(c_3_^WT^(t) + c_3_^M^(t))/2*, where *c_3_^M^(t)* denotes the concentration of mutated PMN in peripheral blood and *c_3_^WT^(t)* that of wildtype PMN. The factor ½ accounts for the fact that the mutations are assumed to be heterozygous. The maximal possible VAF is 0.5 and corresponds to the situation where all circulating neutrophils carry the same heterozygous mutation.

## Results

### The mechanistic model can recapitulate neutrophil engraftment with and without growth factor support

We have developed a mechanistic mathematical model of neutrophil engraftment after allogeneic HSCT. The model accounts for multiple cell types, namely hematopoietic stem cells (HSCs), progenitors, precursors and circulating neutrophils, **Figure 1A**. Important model parameters are the proliferation rate and the self-renewal probability (64). The proliferation rate quantifies how often cells of a given type divide per unit of time. The self-renewal probability corresponds to the probability that a cell arising from division belongs to the same type as its parent cell, e.g., an HSC gives rise to an HSC (64). In the model HSCs are the only cell type with indefinite self-renewal potential, all other considered cell types enter terminal differentiation after a finite number of divisions.

Our model considers multiple non-linear feedback loops linking the mature cell count to immature cell kinetics. If there is a shortage of mature cells, the division rates of immature cells and the number of divisions they perform before terminal differentiation increase. These assumptions are in line with the effects of G-CSF and its analogues (78). Furthermore, in agreement with previous findings (38, 40, 41, 53) we assume that HSC self-renewal increases if there is a shortage of mature cells. The model is calibrated using data from literature about the composition of CD34+ cell grafts and neutrophil kinetics after transplantation. The parameterized model can recapitulate the timely neutrophil engraftment after HSCT with and without cytokine support **Figure 1B**. The time evolution of the immature cell types is depicted in **Supplementary Figures 4**, **5**.

To obtain insights in the inter-individual heterogeneity we design a cohort of 1000 virtual patients, each characterized by individual model parameters. The individual parameters are obtained by random perturbation of the calibrated model parameters. The variation of the times to neutrophil engraftment observed in the virtual patient cohort agrees with the ranges reported in clinical trials [11-25 days (79)], **Figure 1C**. The same applies to the impact of cytokine support on neutrophil engraftment [median reduction of 3 days due to growth factor support (81)], **Figure 1D**.

In summary, we have derived and calibrated a mechanistic model of neutrophil engraftment after allogeneic HSCT. The model can recapitulate neutrophil dynamics in presence and absence of cytokine support.

### Different patterns of clonal expansion after HSCT have been observed

Before we investigate the potential mechanisms governing clonal expansion after allogenic HSCT we provide an overview of frequently observed dynamical patterns. We focus on scenarios where a donor CHIP clone engrafts in the recipient, which is the case in a majority of patients (25, 29). Clonal expansion after HSCT is highly heterogeneous. **Figure 2** shows sketches of different expansion patterns which have been observed in recipients of allogeneic HSCT (23, 29). In many patients the VAF in the recipient’s PB 100 days post transplantation exceeds the VAF in the stem cell graft and in the donor’s PB, **Figures 2A, B**. Frequently, the growth rate of the VAF is maximal in the first weeks after transplantation and slows down thereafter. Several months later the VAF often stabilizes at a constant value which is below the maximal possible VAF of 0.5 (heterozyguous mutation) or 1 (homozyguous mutation) (23, 29). We refer to this pattern as *saturated expansion*, **Figure 2A**. In some individuals exhibiting saturated expansion the VAF shows an initial overshoot before it stabilizes.

In other individuals the VAF can increase over many years at practically constant rates. We refer to this scenario as *persistent expansion*, **Figure 2B**. Naturally, also in these patients the VAF expansion will eventually saturate when it approaches its maximal possible value of 0.5 (heterozygous) or 1 (homozyugous).

**Figure 2C** depicts the scenario where the recipient’s VAF is practically identical to the donor’s VAF. We refer to this as scenario as *no expansion*. Before we compare the model dynamics to patient data, we will investigate which mechanisms could underlie the different growth patterns shown in **Figures 2A–C**. For this sake, we extend the calibrated model described in the previous section to account for one CHIP clone. The mutated cells show the same compartmental organization as the wildtype cells, however, they are characterized by different model parameters (proliferation rate, self-renewal probability, clearance rate) and different responses to feedback signals or mediators of inflammation. An overview of the extended model is shown in **Figure 2D**.

### Aberrant response to growth factors can explain saturated expansion of donor clones after transplantation

**Figure 3:**
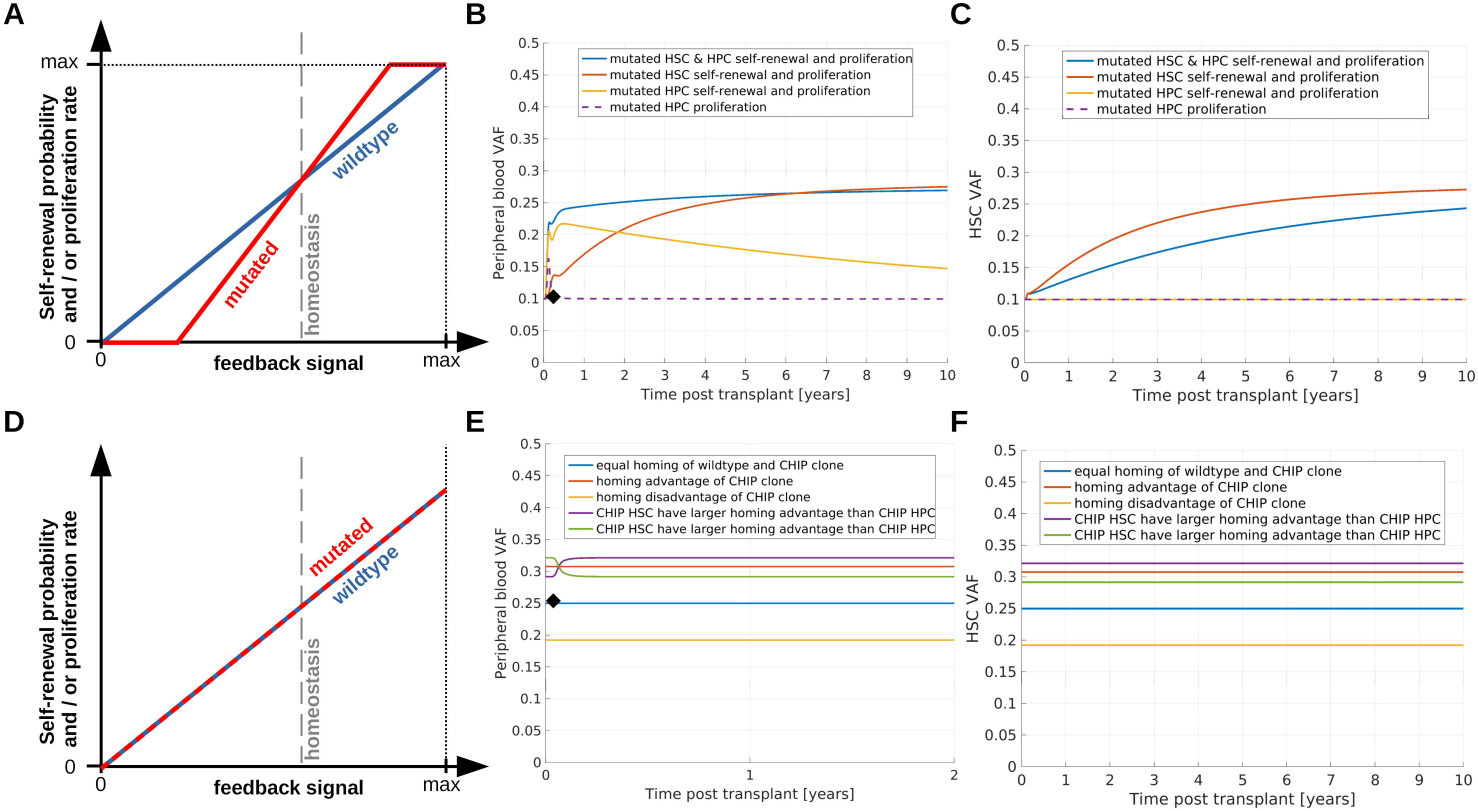
Simulation of saturated clonal expansion. **(A–C)** Consider a scenario which can explain saturated expansion. **(A)** Dependence of immature cell proliferation and self-renewal on the concentrations of the feedback signals. For homeostatic values of the feedback signals mutated and wildtype cells have identical proliferation rates and self-renewal probabilities. Mutated cells are assumed to respond more sensitively compared to wildtpye cells if the homeostatic feedback signal concentrations are perturbed. This implies that an increase of the feedback signals after HSCT confers a growth advantage to mutated cells which leads to an increase of the VAF. **(B)** Recipient PB VAF after transplantation. Donor PB VAF is indicated as black diamond. Dashed purple line: Mutated progenitor cell proliferation responds more sensitively to changes of the feedback signal compared to wildtype progenitor cell proliferation, all other mutated cell properties are identical to the respective wildtype cell properties. Since there is no growth advantage for the mutated stem cells, the VAF increase is only temporary. Yellow line: Mutated progenitor cell proliferation and amplification (number of divisions before terminal differentiation) depend more sensitively on the feedback signals compared to the respective properties of wildtpye progenitors. In this scenario progenitors expand in the months after transplantation. The longer the growth stimulus persists, the more mutated progenitors are generated. The immature progenitors can persist in the system for long times, similar as short term HSCs. Therefore, we observe an increase of VAF followed by a slow decline. Red line: Mutated HSC self-renewal probability and proliferation rate depend more sensitively on the feedback signals compared to the respective wildtype cell properties. This leads to a growth advantage of mutated HSCs and a long-lasting increase of the recipient's VAF compared to the donor's VAF. Blue line: Properties of mutated stem and progenitor cells depend more sensitively on the feedback signals compared to wildtype cells. **(C)** VAF in the recipient’s stem cell compartment. Line colors correspond to the same scenarios as in **(B)**. To observe an increase of VAF in the stem cell compartment, HSC self-renewal probability or proliferation rate have to depend more sensitively on the feedback signals compared to the respective wildtype cell properties. **(D–F)** Show how different homing capabilities impact on clonal dynamics after transplantation. **(D)** Mutated stem and progenitor cells have the same self-renewal probabilities and proliferation rates as the respective wildtype cells. **(E)** Mutated and wildtype cells differ only with respect to their homing capability, i.e., the percentage of transplanted wildtype cells homing to the bone marrow differs from the percentage of transplanted mutated cells homing to the bone marrow. PB VAF is shown in **(E)**, VAF in the HSC compartment in **(F)**. Blue line: 50% of transplanted wildtype cells and 50% of transplanted mutated cells home to the marrow, red line: 50% of transplanted wildtype cells and 80% of transplanted mutated cells home to the marrow, yellow line: 80% of transplanted wildtype cells and 50% of transplanted mutated cells home to the marrow, purple line: 50% of transplanted wildtype cells, 90% of transplanted mutated HSCs and 70% transplanted mutated HPCs home to the marrow, green line: 50% of transplanted wildtype cells, 70% of transplanted mutated HSCs and 90% transplanted mutated HPCs home to the marrow.

We ask whether the saturated expansion of CHIP clones as shown in **Figure 2A** can be explained by a growth advantage of mutated cells under stress conditions. For this purpose, we consider a scenario where mutated cells respond more sensitively to perturbations of the endogenous feedback signals (accounting for growth factors such as G-CSF and GM-CSF) than wildtype cells. We assume that mutated and wildtype cells have the same proliferation rates and self-renewal probabilities under homeostatic conditions, however, a perturbation of the endogenous feedback signal leads to more pronounced changes in mutated compared to wildtype cell kinetics, **Figure 3A**. This corresponds to a scenario where the mutant clone is stable under homeostatic conditions and expands only in the presence of increased growth factor concentrations as they occur due to inflammation/infection or after transplantation. Such a mechanism can explain the observation that in many individuals VAFs remain constant over multiple years (86, 87).

The simulations shown in **Figures 3B, C** suggest that these assumptions are sufficient to recapitulate saturated expansion dynamics. Since mutated cells respond more sensitively to growth factors, the donor-derived mutant clone expands at a higher rate as long as it is exposed to a growth stimulus. This results in an increase of the VAF after HSCT. Over time the cell counts approach their homeostatic values and the growth stimulus decreases. This leads to a reduction of the growth advantage of the CHIP clone and a consecutive slow down of its expansion.

Only if the mutated HSCs harbor at least a temporary growth advantage, the recipient's VAF can stay above the donor's VAF for extended periods of time. If only mutated progenitor and precursor cells exhibit a growth advantage during hematopoietic stress, the VAF first increases and then declines, **Figure 3B** (yellow and purple lines).

Clinical studies suggest that over multiple years the HSC counts in recipients of allogeneic HSCT do not reach the homeostatic HSC counts measured in the donors or healthy individuals (88). It is unknown whether the chronically reduced stem cell counts are linked to a chronic growth stimulus and a subsequent chronic but slow expansion of the HSCs or whether the stem cell expansion stops as soon as the HSC numbers suffice to maintain physiological peripheral cell counts (89). Depending on whether the growth stimulus persists chronically or ceases, the growth advantage of mutated cells may be present for longer or shorter time periods. Correspondingly, the VAF will stabilize earlier or later after transplantation.

In summary, an aberrant response of CHIP clones to endogenous growth factors can recapitulate the dynamics of saturated expansion.

### Homing advantages of mutated cells can explain saturated expansion of donor clones

In a subset of CHIP cases the VAF remains practically constant over multiple years (86) and the triggers leading to its expansion are unknown. In individuals with a constant VAF, the mutated clone may not exhibit a growth advantage at the time of HSPC donation and transplantation. Motivated by this observation, the following section considers scenarios which can lead to a transplantation-related increase of the VAF in absence of a growth advantage of mutated cells.

Most probably not all transplanted stem cells successfully home to the bone marrow and contribute to blood cell formation. Let us assume that mutated and wildtype cells have identical kinetic properties, such as self-renewal probabilities, proliferation rates and clearances. If the percentage of mutated stem cells homing to the host’s marrow is higher than the percentage of wildtype stem cells, we observe an increased VAF in the host compared to the donor. Such a scenario is shown in **Figures 3D–F**. Conversely a homing disadvantage can lead to a reduced VAF in the recipient or to no engraftment of a CHIP clone. In the example simulations in **Figure 3** we assume that mutated and wild type cells solely differ with respect to their homing capabilities. Consequently, the CHIP clone does not expand under homeostatic conditions. This is in line with the finding that VAFs can remain constant over many years (86, 87). Which conditions trigger the expansion of such clones is not well understood. Potential triggers are inflammatory signals or infections (10, 11).

If the percentage of transplanted HSCs homing to the marrow is identical to that of transplanted HPCs, the VAF remains constant as soon as the homing is completed, **Figure 3E** (red, blue, yellow lines). This is accomplished most probably a few days after transplantation in consideration of the fast clearance of infused CD34+ cells from blood stream (80). Consequently, the VAF is virtually constant in all follow up samples. This, however, is rarely observed. In most cases the VAF dynamically changes in the first months after HSCT. If the percentage of mutated HSCs homing to the marrow is higher than the percentage of mutated HPCs homing to the marrow, the VAF in PB increases before it reaches a constant value, **Figure 3E** (purple line). In the opposite case, i.e., if the percentage of mutated HPCs homing to the marrow is higher than the percentage of mutated HSCs homing to the marrow, the VAF in PB declines before it reaches a constant value, **Figure 3E** (green line). We have repeated simulations for many different parameter sets and we have always observed either constant PB VAFs or monotonous VAF changes eventually converging to a constant value. Non-monotonous VAF dynamics such as overshoots have not been observed if mutated and wildtype cells differ only with respect to their homing dynamics. If we assume that mutated cells are preferentially mobilized and over-represented in the graft, we observe identical dynamics as in the case where mutated cells have homing advantages.

We conclude that a homing advantage of mutated cells or an over-representation of mutated cells in the graft are sufficient to explain saturated expansion independent of mutation-related alterations of cell kinetics. In combination with the mechanisms considered in the previous and in the next section, homing advantages can amplify the VAF increase which is triggered by growth advantages of the mutated cells.

### Persistent expansion of donor clones after transplantation requires permanent alterations of kinetic cell properties or ongoing selective stimulation of mutated cells

**Figure 4:**
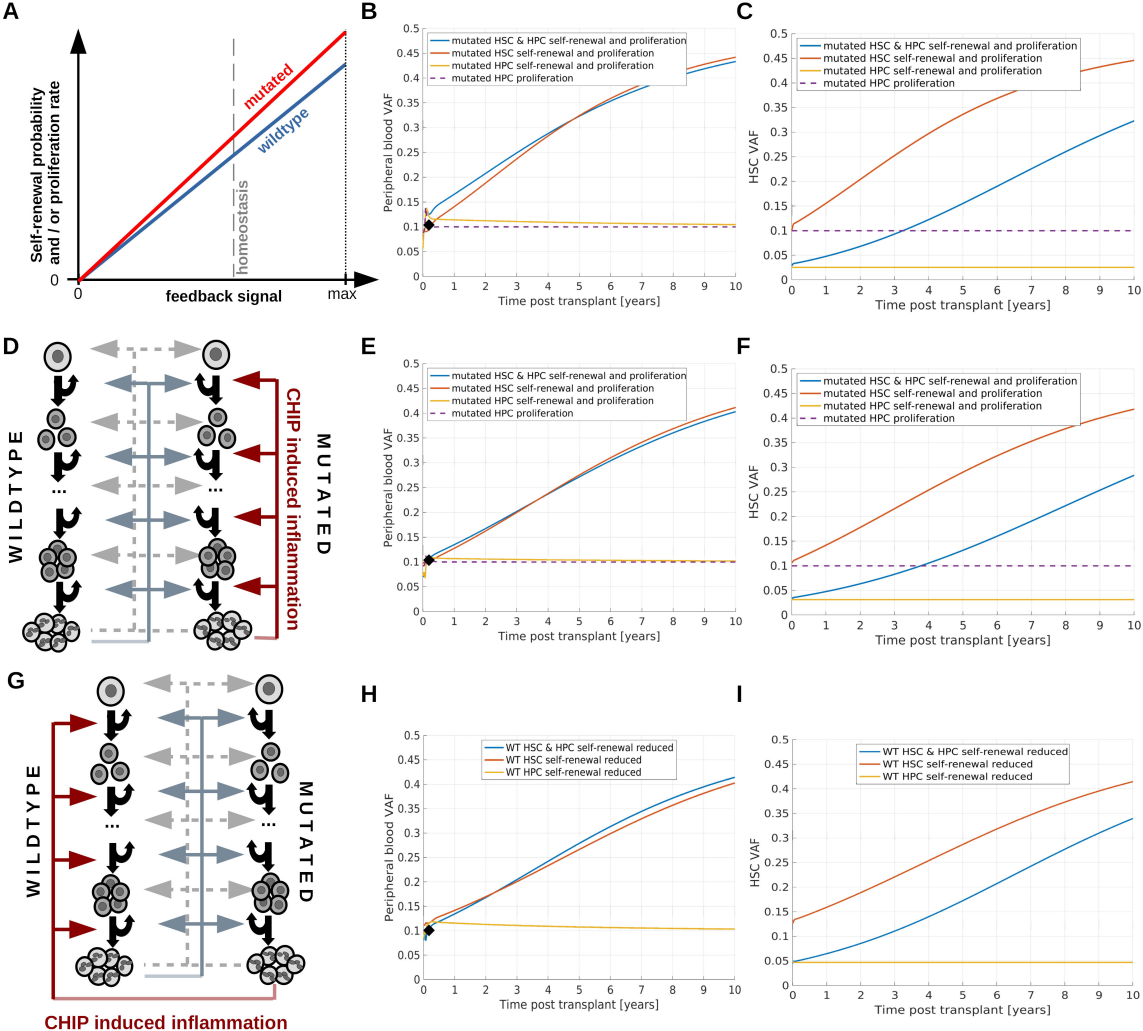
Simulation of persistent clonal expansion. **(A–C)** Consider a scenario where persistent clonal expansion is driven by increased HSC self-renewal and proliferation of mutated compared to wildtype cells. **(A)** Dependence of self-renewal probability and proliferation rate on the respective feedback signals. For each value of the feedback signal, the self-renewal and proliferation of mutated cells is higher compared to that of wildtype cells. **(B)** Dynamics of PB VAF after HSCT. The black diamond indicates the VAF in the donor’s PB. Blue line: Mutated HSC and HPC proliferation, mutated HSC self-renewal probability and mutated HPC amplification (cell divisions before terminal differentiation) are higher compared to wild type cells. Red line: Mutated HSC self-renewal probability and proliferation rate are higher than the respective properties of wildtype cells. Yellow line: Mutated HPC proliferation rate and amplification are increased compared to the respective properties of wildtype HPCs. Since HSC properties are identical for mutated and wildtype cells, we observe transient but no persisting changes of the VAF. Purple line: Mutated HPC proliferation rate is increased compared to wildtype HPC proliferation rate. Also in this case we observe only temporary changes of the recipient’s VAF. **(C)** VAF dynamics in the stem cell compartment. Color coding as in **(B)**. If the HPC amplification of mutated cells is higher compared to wildtype cells, i.e., mutated progenitors perform a higher number of divisions before terminal differentiation than wildtype progenitors, the VAF in PB is higher than in the HSC compartment. This applies to the simulations shown as yellow and blue lines. **(D–F)** consider a scenario where CHIP-induced chronic inflammation triggers persistent mutated cell expansion. **(D)** Mutated white blood cells trigger chronic systemic inflammation which in turn confers a competitive advantage to mutated HSCs and HPCs by increasing proliferation rates, HSC self-renewal probabilities and HPC amplification. **(E)** Dynamics of PB VAF after HSCT. The black diamond indicates the VAF in the donor’s PB. Blue line: Mutated HSC and HPC proliferation, mutated HSC self-renewal probability and mutated HPC amplification are increased in response to the inflammatory cytokines. Red line: Mutated HSC self-renewal probability and proliferation rate are increased in response to the inflammatory cytokines. Yellow line: Mutated HPC proliferation rate and amplification are increased in response to the inflammatory cytokines. Since HSC properties are identical for mutated and wildtype cells, we observe no persistent VAF increase. Purple line: Mutated HPC proliferation rate is increased in response to the inflammatory cytokines. In all scenarios the chronic inflammation does not affect wildtype cells. **(F)** VAF dynamics in the stem cell compartment. Color coding as in **(E)**. **(G–I)** consider a scenario where CHIP-induced chronic inflammation reduces the fitness of wildtype cells. **(G)** Mutated white blood cells trigger chronic inflammation. The higher the inflammatory burden, the lower the self-renewal probability of wildtype HSC and the amplification of wildtype progenitors. **(H)** Dynamics of PB VAF after HSCT. The black diamond indicates the VAF in the donor’s PB. Blue line: Wildtype HSC self-renewal probability and HPC amplification are reduced in response to the inflammatory cytokines. Red line: Wildtype HSC self-renewal probability is decreased in response to the inflammatory cytokines. Yellow line: Wildtype HPC amplification is reduced in response to the inflammatory cytokines. Since HSC properties are identical for mutated and wildtype cells, we observe no persistent VAF increase. **(I)** VAF dynamics in the stem cell compartment. Color coding as in **(G)**. If we assume that chronic inflammation increases wildtype cell proliferation rate in addition to the effects considered in **(G–I)**, the VAF dynamics is faster, see **Supplementary Figure 6**.

Inspired by patho-physiological hypotheses we consider three mechanisms which can lead to persistent expansion of CHIP clones. Mechanism 1: CHIP mutations lead to a persistent growth advantage mediated by an increased stem cell self-renewal probability which can be accompanied by an increased proliferation rate. Such changes have been observed for the loss of Tet2 or Dnmt3a (90, 91, 10). Example simulations are provided in **Figures 4A–C**. Mechanism 2: CHIP mutations trigger the expression of pro-inflammatory genes in neutrophils and monocytes which lead to chronic systemic inflammation. The inflammatory cytokines confer a relative growth advantage to mutated cells by increasing their self-renewal and proliferation. There is evidence that this mechanism plays a role in the case of Tet2 or Dnmt3 mutations (10, 11). Example simulations are shown in **Figures 4D–F**. Mechanism 3: CHIP mutations trigger the expression of pro-inflammatory genes in neutrophils and monocytes which lead to chronic systemic inflammation. The inflammatory cytokines confer a relative growth disadvantage to wildtype cells by reducing their self-renewal probability. This increases the probability that cycling stem cells get lost due to differentiation events. This model is based on findings in zebrafish suggesting that mutated cells are resistant to the effects of chronic inflammation which drive wildtype cells into replicative exhaustion (85). Example simulations are shown in **Figures 4G–I** and **Supplementary Figure 6**. All three mechanisms can lead to persistent expansion of CHIP clones in the donor as well as in the recipient, if they affect HSCs. As long as only progenitors are affected no persistent expansion is observed (yellow and purple lines in **Figure 4**).

### The mechanistic model can recapitulate different clonal growth patterns observed after allogeneic hematopoietic stem cell transplantation

**Figure 5:**
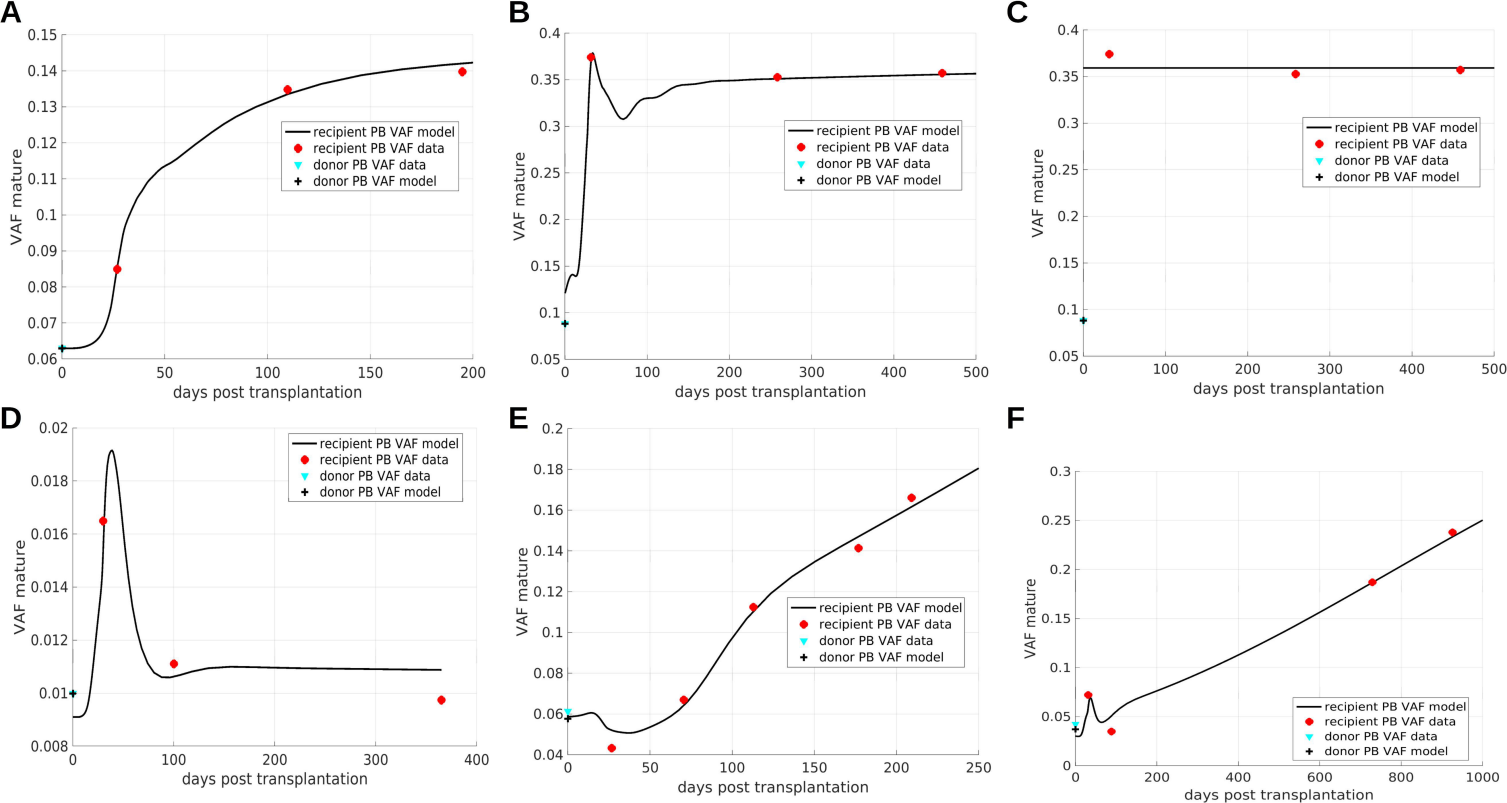
Comparison of model simulations to patient data. **(A–D)** Show saturated expansion, **(E, F)** exhibit persistent clonal expansion. The overshoots result from a proliferative advantage of mutated progenitor cells. Persistent clonal expansion is triggered by a mutation-related increase of the HSC self-renewal probability. The simulations serve as a proof of concept and parameters are not necessarily unique. The data in panel **(B, C)** is identical. In panel **(B)** the VAF expansion results from a combination of different mechanisms: aberrant response of mutated cells to feedback signals, increased proliferation of mutated progenitors compared to wildtype progenitors, and preferential homing of mutated cells. In **(C)** the preferential homing of mutated cells is more pronounced compared to **(B)** and is the only mechanisms leading to VAF expansion. **(A–C, E, F)** data from (23), **(D)** data from (29).

In the previous sections we have considered qualitative simulations to provide insights in the mechanisms governing clonal dynamics after HSCT. In this section, we compare the model to longitudinal VAF data from literature. We observe that the model can reproduce a broad range of dynamic patterns leading to saturated, **Figures 5A–D**, or persisting clonal expansion, **Figures 5E, F**.

The saturated expansion in **Figure 5A** can be reproduced assuming that mutated HSCs and HPCs respond more sensitively to increased cytokine concentrations than their wildtype counterparts (for illustration see **Figure 3A**). The data in **Figures 5B, C** are identical. In **Figure 5B** the VAF expansion is achieved by a more sensitive response of mutated cells to changes of the feedback signals in combination with a proliferative and amplification advantage of mutated progenitors and a preferential homing of mutated cells. In **Figure 5C** the VAF expansion is solely triggered by preferential homing of mutated cells to the bone marrow. This illustrates that the same data set can be explained by different patho-physiological mechanisms.

**Figure 5D** shows an example for a transient increase of the VAF. This can be reproduced assuming that only mutated HPCs but not HSCs have a growth advantage in the post-transplantation period. The driver of the persistent clonal expansion in **Figures 5E, F** is a permanent increase of mutated HSC self-renewal.

If mutated progenitors have a proliferative advantage and are exposed to increased feedback signals after HSCT, this leads to a temporary overshoot of mature mutated cells as it is observed in **Figures 5B, D, F**. The temporary decline of VAF observed in **Figures 5E, F** follows from a high mutant HSC self-renewal probability. After HSCT high concentrations of the feedback signal increase the HSC self-renewal probability. Therefore, in the early post-transplantation period mutant HSCs give rise nearly exclusively to mutant HSCs and not to progenitors, which leads to a temporary decrease of the PB VAF. An example of a patient harboring two CHIP clones is provided in **Supplementary Figure 7**.

The model simulations shown in **Figure 5** are to be understood as a proof of principle. Due to the sparsity of the available data, the parameters and mechanisms which are sufficient to recapitulate the VAF dynamics are not unique. **Figure 5** suggests that the proposed modeling framework can capture real-world clonal dynamics.

### Comparison of clonal dynamics in donor and recipient reveals accelerated clonal expansion in the first years after transplantation

**Figure 6:**
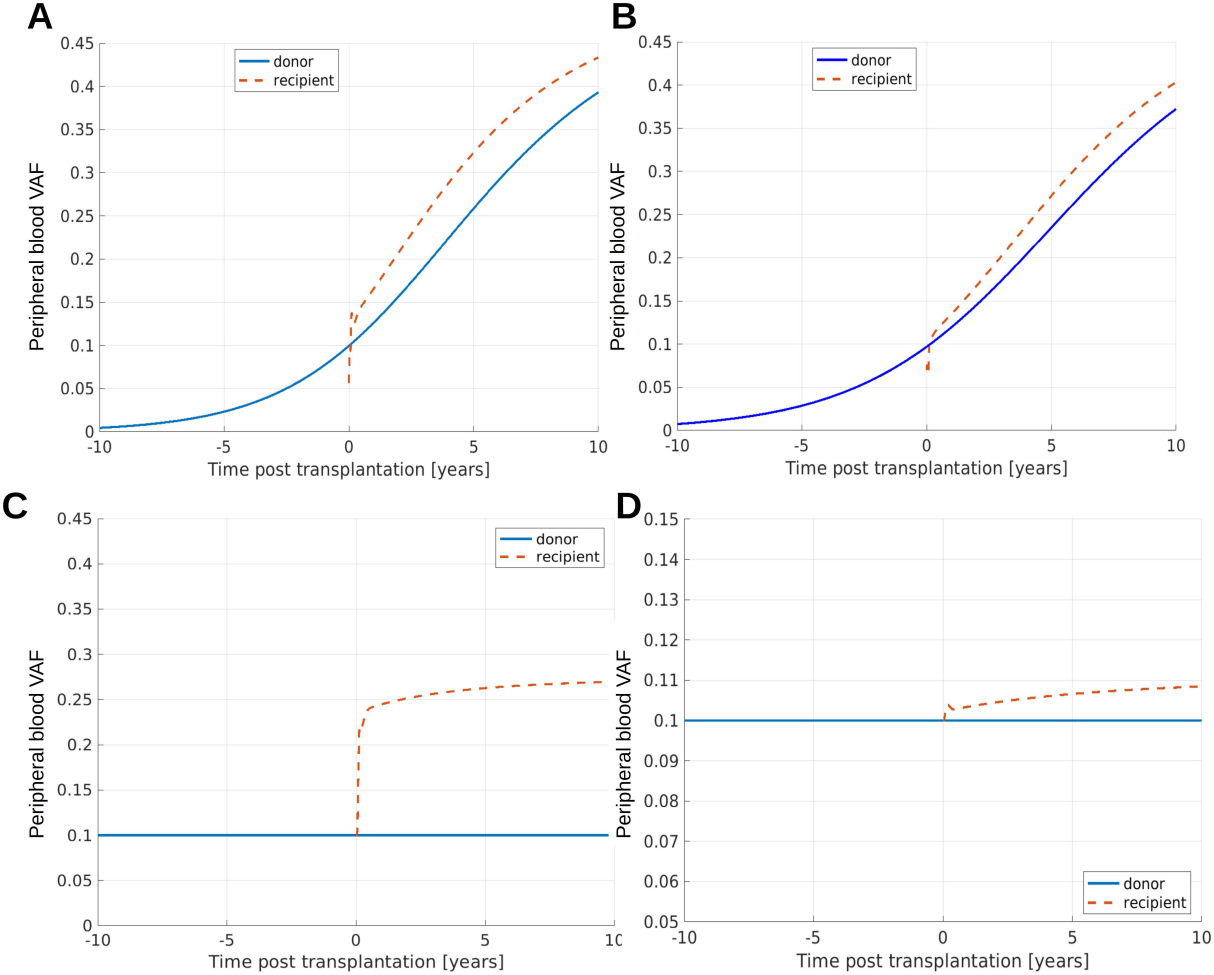
Clonal dynamics in donor and recipient. **(A, B)** Show scenarios where the mutant clone exhibits persistent expansion in the donor and in the recipient. The model parameters in **(A)** are identical to those from **Figures 4B, C**, the model parameters in **(B)** are identical to those from **Figures 4E, F**. In the recipient we observe quick changes of VAF in the first months after transplantation. After that, the difference of recipient VAF and donor VAF changes considerably slower or remains practically constant. The difference observed in the simulations is qualitatively in line with the measurements in (92). **(C, D)** Show scenarios where the donor VAF is stable over time and the recipient exhibits saturated expansion. The precise value of the difference of donor and recipient VAF depends on the mutated cell parameters. The parameters in **(C)** are identical to the parameters in **Figures 3B, C**.

It is an interesting question how the VAF evolves in the donor compared to the recipient. We have performed simulations of VAF dynamics in the donor and recipient. In our simulations we observe that recipients’ PB VAFs can quickly change in the first year after transplantation. After that the difference of PB VAF in donor and recipient remains approximately constant for multiple years. This applies to both persistent expansion (**Figures 6A, B**) and saturated expansion (**Figures 6C, D**). This observation is in line with a recent trial (92) comparing donor and recipient PB VAF in long term survivors after HSCT. The study (92) identifies 5 donor-recipient pairs where donor-derived clones are detectable in the recipient multiple years after HSCT. In median 15 years after transplantation an increase of the PB VAF in the recipient compared to the donor is detected. The absolute VAF increase reported in (92) is in the order of 0.1 or below. Observations from (23) suggest that more pronounced increases of the VAF might be possible, see **Figure 5B**. In our qualitative simulations we observe that the precise value of the VAF difference in recipients and donors depends on the mutated cell parameters and can be above or below 0.1. The qualitative dynamics of our model are in agreement with the observations in (92).

### The number of transplanted cells impacts on the clonal growth dynamics after transplantation

One important parameter during HSCT is the number of transplanted CD34+ cells per kg of body weight. We refer to this quantity as *transplant dose*. To study how the transplant dose impacts on the clonal dynamics, we simulate HSCTs in an *in silico* trial cohort consisting of 1000 virtual patients. The advantage of this approach is that it allows to account for the inter-individual heterogeneity. We consider the same virtual patients as in Section 1, since this cohort reproduces the inter-individual variation of the time to neutrophil engraftment reported in clinical trials. The properties of CHIP cells and the VAFs at the time of donation are randomly chosen. We simulate transplantation of different cell doses ranging from 1x10^6^ to 1.5x10^7^ CD34+ cells per kg of body weight. These are clinically realistic quantities (79). Unlike clinical trials, where each patient can only be transplanted once at a given time, the *in silico* approach allows to compare how the exactly same recipients respond to different transplant doses.

**Figure 7:**
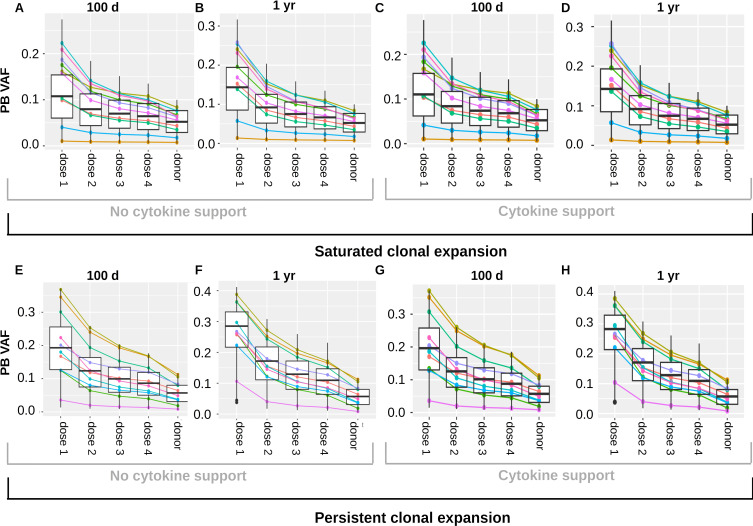
Impact of transplant dose on clonal dynamics. The figure summarizes the results of a virtual clinical trial. **(A–D)** depict a cohort with saturated clonal expansion, **(E–H)** depict a cohort with persistent clonal expansion. Each cohort consists of 1000 virtual patients. The colored lines visualize the results for 10 randomly chosen virtual patients. Donors’ PB VAFs are between 0.005 and 0.125. Different cell doses were transplanted and the recipient VAFs in PB were recorded 100 days and one year after transplantation. For the sake of direct comparison all results shown in **(A–D)** were obtained from the same virtual patient cohort. The columns “dose 1”, “dose 2”, “dose 3”, dose 4” show the recipients’ VAFs 100 days or 1 year after transplantation of the cell doses 1-4. The column “donor” shows the PB VAF of the donors. Analogously, all results shown in **(E–H)** were obtained from the same virtual patient cohort. **(A, C, E, G)** relate the PB donor VAF to the PB recipient VAF 100 days after transplantation. **(B, D, F, H)** relate the PB donor VAF to the PB recipient VAF 1 year after transplantation. In **(A, B, E, F)** no growth factors were administered after transplantation, in **(C, D, G, H)** one dose of pegfilgrastim was administered at day 3 after transplantation. Dose 1: 1x10^6^ CD34+ cells per kg of body weight, dose 2: 5x10^6^ CD34+ cells per kg of body weight, dose 3: 1x10^7^ CD34+ cells per kg of body weight, dose 4: 1.5x10^7^ CD34+ cells per kg of body weight.

We simulate engraftment and clonal dynamics in each virtual patient for each of the considered transplant doses. We compare how the VAFs at 100 days and 1 year after transplantation differ for different doses of transplanted cells. We run separate simulations for CHIP cell characteristics leading to saturated clonal expansion (**Figures 7A–D**) and persistent clonal expansion (**Figures 7E–H**). We observe that the recipient PB VAF is higher for small compared to large transplant doses.

The mechanism underlying this observation is as follows: The less cells are transplanted the more they have to expand to reestablish the physiological state. Mutated cells have a growth advantage during the expansion period. The higher the factor by which the transplanted cells have to expand, the longer the expansion period and the more mutated cells are produced compared to wildtype cells. This applies to saturated and persistent clonal expansion. As expected, the recipients exhibiting persistent clonal expansion show an ongoing VAF increase after transplantation.

A well known approach to speed up neutrophil engraftment is the administration of hematopoietic growth factors such as pegfilgrastim after the transplantation (81, 82). We repeat all simulations in the presence of growth factor support to check whether this might affect the expansion of CHIP clones in the recipient. Results for saturated clonal expansion are shown in **Figures 7C, D**, results for persistent expansion in **Figures 7G, H**. For the simulations we assume that in presence of growth factors the proliferation rates, HSC self-renewal probability and HPC amplification increase in a dose dependent manner. The growth factors are assumed to increase the stimulating feedback signals acting on both wildtype and mutated cell kinetics. Under these assumptions the cytokine administration has no relevant impact on the clonal expansion dynamics in the recipient. **Figure 7** is based on a model where stem cell proliferation and self-renewal are regulated by systemic feedback signals. We repeated all simulations for two modified versions of the model (**Supplementary Figures 8**–**13**). In the first modification, we assume that HSC self-renewal is regulated by the stem cell niche (**Supplementary Figure 9**). In the second modification, we assume that HSC self-renewal and proliferation are regulated by the stem cell niche (**Supplementary Figure 12**). The results are very similar to those in **Figure 7**.

### The number of transplanted stem cells and not of transplanted non-stem progenitors impacts on the clonal growth dynamics after transplantation

**Figure 8:**
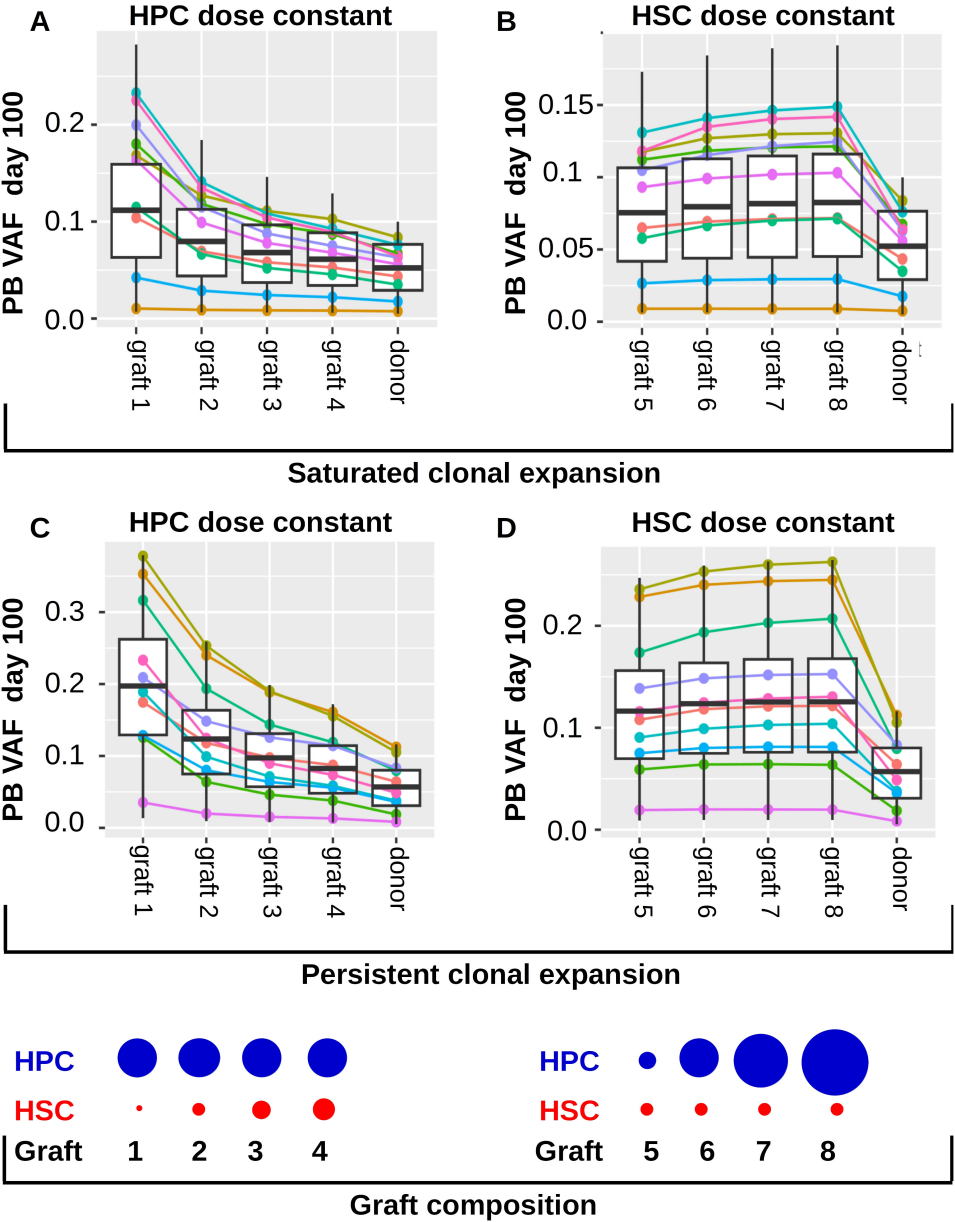
Impact of transplant composition on clonal expansion dynamics in the recipients. Panels **(A, B)** refer to individuals exhibiting saturated clonal expansion. **(A)** The 1000 virtual patients from **Figures 7A–D** are transplanted with grafts containing different HSC numbers. The ratios of transplanted HSC numbers are as follows: graft 1: graft 2: graft 3: graft 4 = 1: 5: 10: 15. The number of transplanted non-stem progenitors (HPCs) is the same for all virtual patients. The total transplant dose is 5x10^6^ CD34+ cells per kg of body weight. The leftmost 4 columns “graft 1”, “graft 2”, “graft 3”, “graft 4” show the recipients’ VAFs 100 days after transplantation of graft 1, graft 2, graft 3, graft 4, respectively. The rightmost column shows the VAF in the donors’ peripheral blood. We observe that the recipient’s VAFs are higher if the number of transplanted HSCs decreases. The colored lines show the VAFs of 10 randomly selected example virtual patients. **(B)** The 1000 virtual patients from **Figures 7A–D** are transplanted with grafts containing different HPC numbers but the same HSC number. The ratios of HPCs are as follows graft 5: graft 6: graft 7: graft 8 = 1: 5: 10: 15, corresponding to total transplant doses of 1x10^6^, 5x10^6^, 1x10^7^, 1.5 x10^7^ CD34+ cells per kg of body weight. The number of HSCs is the same in all grafts and corresponds to the HSC content of a graft of 5x10^6^ CD34+ cells per kg of body weight. The leftmost 4 columns “graft 5”, “graft 6”, “graft 7”, “graft 8” show the recipients’ VAFs 100 days after transplantation of graft 5, graft 6, graft 7, graft 8, respectively. The rightmost column shows the VAFs in the donors’ peripheral blood. We observe that the recipients’ VAFs are similar for the different grafts. Panels **(C, D)** refer to individuals exhibiting persistent clonal expansion. The virtual patients are identical to those from **Figures 7E–H**. The grafts in **(C)** are as in **(A)**, the grafts in **(D)** are as in **(B)**. The blue and red circles at the bottom of the Figure visualize the transplant composition. We note that graft 2 and graft 6 have have identical compositions.

In this section we investigate whether the differences in the transplanted HSC numbers, the differences in the transplanted non-stem progenitor numbers or both lead to the negative correlation of recipient VAF and transplant dose shown in **Figure 7**. For this reason, we simulate two different clinical trials using the virtual patient cohort from the previous sections. The results for virtual patients exhibiting saturated clonal expansion are shown in **Figures 8A, B**, the results for virtual patients exhibiting persistent clonal expansion are depicted in **Figures 8C, D**. In the first simulated trial (**Figures 8A, C**) the transplanted grafts differ only with respect to the HSC numbers. The number of non-stem progenitors is the same for all virtual patients. Similar as in **Figure 7** we observe that the recipients’ VAFs increases if the number of transplanted HSCs decreases. In the second simulated trial (**Figures 8B, D**) the transplanted grafts differ only with respect to the numbers of non-stem progenitor cells. The number of transplanted HSCs is the same for all virtual patients. In this setting we observe very similar VAFs for all virtual patients. Therefore, we conclude that the number of transplanted HSCs but not of non-stem progenitors is an important determinant of the recipient’s VAF in the considered model. The results in **Figure 8** refer to the setting without post transplant growth factor support. In case of growth factor support analogous dynamics are observed. **Figure 8** is based on a model where stem cell proliferation and self-renewal are regulated by systemic feedback signals. If we assume that HSC self-renewal (**Supplementary Figure 10**) or both HSC self-renewal and proliferation (**Supplementary Figure 13**) are regulated by the stem cell niche, we obtain similar results.

### The VAF increase in the donor’s PB one year prior to HSPC donation correlates with the expansion of the mutated clone in response to transplantation

**Figure 9:**
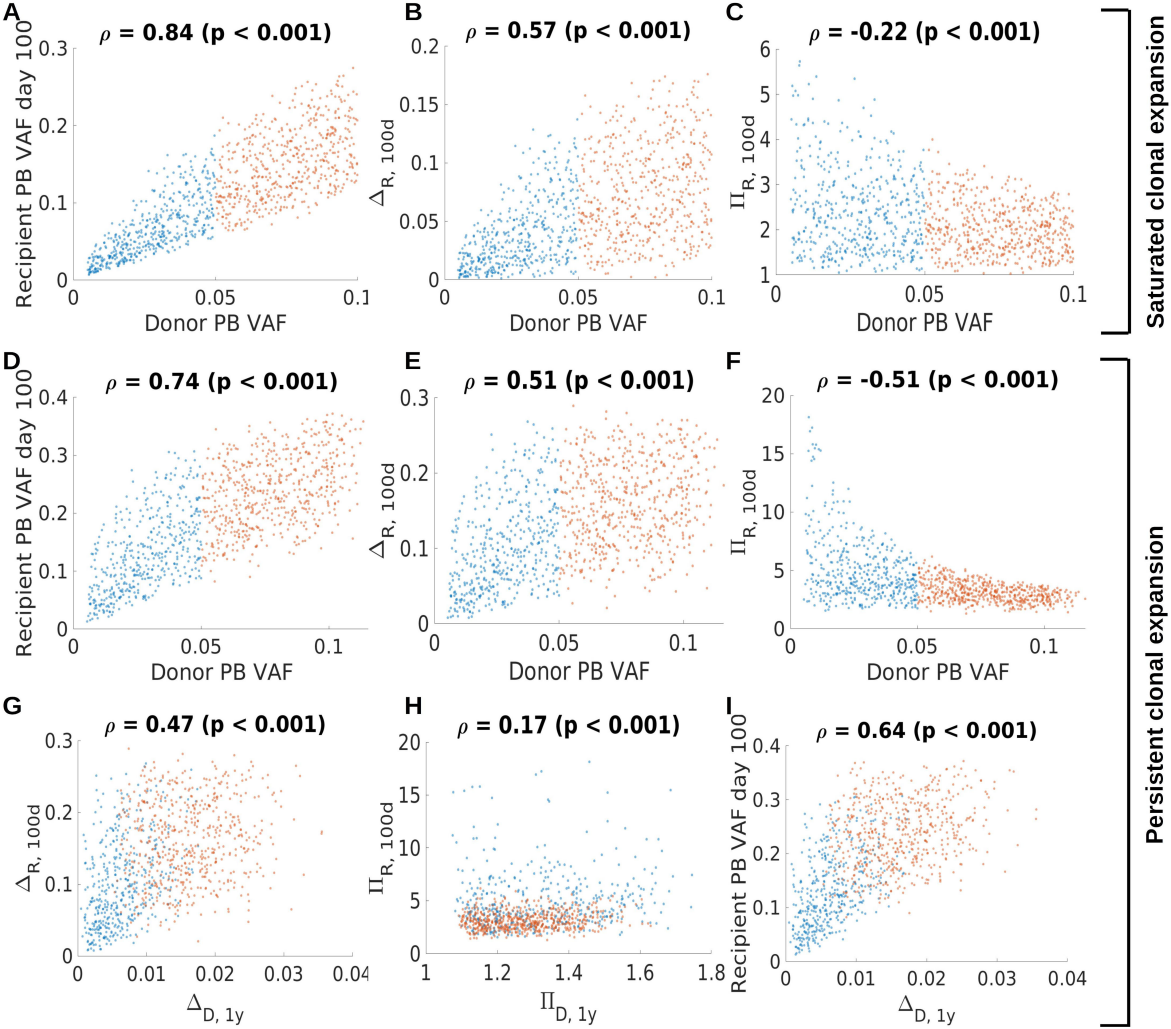
Association of donor and recipient clonal dynamics. Panels **(A–C)** refer to saturated clonal expansion. There is positive association (Spearman’ rank correlation coefficient) between donor and recipient VAF **(A)** and between donor VAF and the absolute clonal expansion within the first 100 days after transplantation **(B)**. The difference of recipient PB VAF at day 100 and the donor PB VAF at the time of donation is referred to as Δ_R,100d_. There is a negative association between the donor PB VAF and the relative clonal expansion within the first 100 days after transplantation. The ratio of the recipient PB VAF at day 100 and the donor PB VAF at the time of donation is referred to as π_R,100d_. This indicates that large donor clones show less relative increase compared to smaller donor clones. Panels **(D–I)** refer to persistent clonal expansion. Similar associations as in **(A–C)** hold for virtual patients exhibiting persistent clonal expansion, panels **(C–F)**. The relative clonal expansion within the first 100 days is higher for clones exhibiting persistent expansion (panel **F**) compared to clones exhibiting saturated expansion (panel **C**). Panels **(G–I)** relate the clonal dynamics in the donor one year prior to donation to the clonal dynamics in the recipient. The absolute clonal expansion in the donor one year before donation, Δ_D,1y_, is positively correlated with the absolute clonal expansion in the recipient within in the first 100 days after transplantation, Δ_R,100d_, as shown in **(G)**. Δ_D,1y_ is defined as the difference of the donor’s PB VAF at the time of donation and the donor’s PB VAF one year prior to donation. There is a weak association between the relative clonal expansion in the donor one year before donation, π_D,1y_, and the relative clonal expansion within the first 100 days after transplantation **(H)**. π_D,1y_ is defined as the ratio of the donor’s PB VAF at the time of donation and the donor’s PB VAF one year prior to donation. The absolute clonal expansion in the donor is positively associated with the recipient PB VAF on day 100 **(I)**. Red dots correspond to donor PB VAF above 0.05, blue dots to donor PB VAF of 0.05 or below.

To better understand whether the recipient VAF and the expansion of the mutated clone in response to HSCT can be predicted, we calculated Spearman’s rank correlation coefficients for different quantities related to the donor and recipient clonal dynamics. We separately analyzed the virtual patient cohort exhibiting saturated clonal expansion (**Figures 9A–C**) and the virtual patient cohort exhibiting persisting clonal expansion (**Figures 9D–I**). Not surprisingly, for both cohorts there is a strong association between donor PB VAF and recipient PB VAF 100 days after transplantation, **Figures 9A, D**. Similarly, there is a positive association of the donor PB VAF and the absolute transplantation-related expansion. We define the latter as the difference of recipient VAF at day 100 and donor VAF, **Figures 9B, E**. This indicates that the absolute increase of VAF due to transplantation is higher for larger donor clones than for smaller donor clones. The relative transplantation-related clonal expansion (recipient VAF at day 100 divided by donor VAF) shows an opposite trend, **Figures 9C, F**. This indicates that only small donor clones (donor PB VAF<0.05) can expand more than 3-fold due to transplantation. Larger donor clones (donor PB VAF>0.05) in our simulations rarely expand more than 3-fold.

For the virtual patient cohort exhibiting persistent clonal expansion we searched for associations between clonal dynamics in the donor one year before transplantation and the transplantation-related clonal expansion. We observe a positive correlation of the absolute donor’s VAF increase in the year before donation (the difference of the VAF at the time of donation and the VAF one year prior to donation) and the transplant related absolute VAF increase (recipient VAF 100 days after transplantation minus donor VAF at the time of donation), **Figure 9G**. For the respective relative quantities (recipient VAF 100 days after transplantation divided by donor VAF at the time of donation, donor VAF at the time of donation divided by donor VAF one year prior to donation), although statistically significant, we observe no relevant association, **Figure 9H**. We also observe that the absolute donor’s VAF increase in the year before donation is positively correlated with the recipient VAF at day 100, **Figure 9I**.

In summary, these analyses suggest that the donor’s VAF and the absolute change of the donor’s VAF in the year before donation might be suitable to roughly estimate the clonal expansion triggered by the transplantation.

### The interplay of CHIP-induced inflammation and inflammation-dependent clonal fitness can lead to complex dynamics

CHIP is closely related to inflammatory comorbidities such as atherosclerosis (18, 93, 94) and it has been shown that chronic inflammation can confer a growth advantage to mutated cells (10, 11). In this section, we consider two handpicked examples to demonstrate the complexity emerging from the interplay of CHIP clones and chronic inflammation.

**Figure 10:**
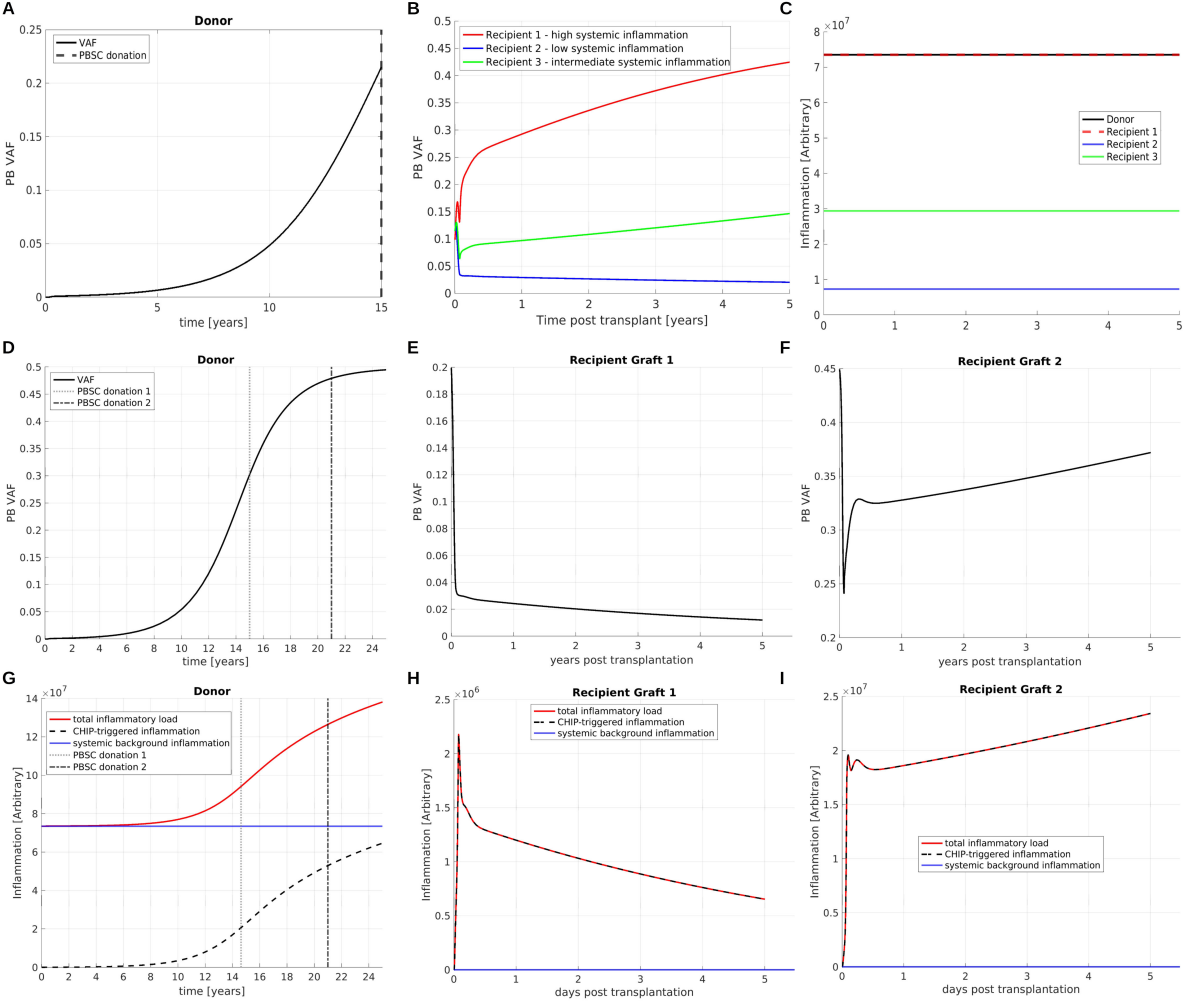
Interplay of CHIP and chronic inflammation. **(A)** VAF dynamics in a donor harboring a CHIP clone. The clone is dependent on chronic inflammation in the sense that it exhibits a growth advantage in presence and a growth disadvantage in absence of systemic inflammatory signals. The time of donation is indicated by a vertical line. **(B)** The donated cells are transplanted into three recipients. Depending on the recipient’s systemic inflammatory burden the CHIP clone expands (red line and green line) at different rates or declines (blue line). **(C)** shows the systemic inflammatory burdens in the donor and the recipients. For simplicity we assume that they are constant in time. **(D)** VAF dynamics in a donor harboring a CHIP clone. As in **(A–C)** the clone is dependent on chronic inflammation. The mutant mature cells secrete pro-inflammatory mediators and thus trigger expansion of the CHIP clone. The donor’s total systemic inflammatory burden is composed of a CHIP-dependent and a CHIP-independent part, as shown in panel **(G)** as dashed black and continuous blue line. Two grafts are obtained at the time points indicated by vertical lines. The grafts are transplanted in two identical recipients. The recipients have a lower systemic inflammatory burden compared to the donor. The clonal expansion dynamics in the recipients are depicted in panels **(E, F)**, the respective systemic inflammatory burdens in **(H, I)**. In recipient 1, shown in Panel **(E)**, we observe a decline of the mutated clone after transplantation, since the total inflammatory burden is too low to confer a sufficient growth advantage to the mutated clone. In recipient 2, shown in panel **(F)**, we observe clonal expansion. Recipient 2 obtained a graft with a higher VAF compared to recipient 1. Since a higher amount of mutated cells causes a higher amount of CHIP-triggered systemic inflammation, the total inflammatory burden in recipient 2 is sufficient to drive clonal expansion.

In both examples we consider CHIP clones which are dependent on chronic inflammation in the sense that they exhibit a growth advantage in presence and a growth disadvantage in absence of chronic inflammation. In the first example (**Figures 10A–C**) we consider a donor harboring a systemic inflammatory burden which is independent of CHIP and e.g., related to atherosclerosis or other forms of inflammaging (95). The donor’s inflammatory burden is sufficiently high to confer a growth advantage to the CHIP clone which leads to its expansion, **Figure 10A**. In this scenario the inflammatory burden in the host decides whether and at which rates the transplanted CHIP clone expands or declines, **Figures 10B, C**. This simulation supports the concept that host factors may decide about expansion and engraftment of CHIP clones.

In the second example (**Figures 10D–I**) we consider a CHIP clone which increases the systemic inflammatory burden, e.g., by giving rise to neutrophils and monocytes expressing increased amounts of pro-inflammatory cytokines, as described in (5). There are concerns that the transplantation of a such a CHIP clone may trigger CHIP-related inflammatory complications in the host. We again consider a donor harboring a systemic inflammatory burden which is independent of CHIP. This inflammatory burden triggers the growth of the CHIP clone, **Figure 10D**. In addition to that, the CHIP clones increases the systemic inflammatory burden and thus stimulates its own expansion, **Figure 10G**.

If, as assumed in the considered example, the CHIP clone has a growth disadvantage in the absence of chronic inflammation its growth dynamics after transplantation may depend on the presence of systemic inflammatory mediators in the host environment. To study this in more detail we simulate two HSCTs. The grafts were obtained from the same donor but at different time points, as indicated in **Figure 10D**. The grafts are transplanted in two identical recipients, referred to as recipient 1 (**Figures 10E, H**) and recipient 2 (**Figures 10F, I**). We assume that the CHIP-independent inflammatory burden in the hosts is lower compared to the donor. The graft collected at the earlier time point has a lower VAF compared to the graft collected at the later time point. Upon transplantation we observe expansion of the CHIP clone for the graft with the higher VAF (**Figure 10F**) and decline of the CHIP clone for the graft with the lower VAF (**Figure 10E**). The reason for this observation is that in the first case the inflammatory mediators secreted by the CHIP clone are sufficient to trigger the mutant cell expansion, whereas in the second case they are below the threshold required to confer a sufficient growth advantage to the mutated cells.

These theoretical examples demonstrate that the host environment can impact on clonal dynamics after HSCT and that a critical VAF might be required to trigger clonal expansion in the host. As long as there is no quantitative knowledge on the response of the mutated cells to the endogenous growth factors it is difficult to quantify or predict which exact number of mutated cells is required for engraftment and expansion of a CHIP clone in a specific individual.

## Discussion

In this work we have developed quantitative computational models of allogenic HSCT. The focus is on the post-transplantation dynamics of donor-derived clones. The models account for relevant patho-mechanisms of CHIP including an aberrant response of mutated cells to feedback signals, a mutation-induced persistent growth advantage, and self-sustaining chronic inflammation. The models can reproduce clonal growth patterns which have been observed in clinical data, **Figure 5**. We simulate virtual clinical trials to study how key parameters of the HSCT, namely the number of stem and progenitor cells transplanted per kg of body weight and the administration of growth factors after transplantation, impact on the expansion of mutated cells.

This work leads to new insights in the mechanisms underlying the expansion of donor-derived clones after allogeneic HSCT. We have focused on the mechanistic underpinning of two frequently observed clonal growth patterns to which we refer as *saturated expansion* and *persistent expansion*. In the case of saturated expansion, donor-derived clones show the fastest expansion in the first months after transplantation, after that the clonal expansion slows down and eventually ceases before the maximal possible VAF is reached. In our simulations such dynamics can be explained by preferential homing of mutated HSCs to the host’s marrow, by over-representation of mutated cells in the graft, or by aberrant response of mutated stem and progenitor cells to endogenous growth factors. Another possible explanation for saturated expansion could be an incomplete immune control of the mutated cells. Potentially, in the donor the size of the mutated clone is kept constant by T-cell immunity and delayed T-cell engraftment after transplantation may lead to a temporal expansion of the mutated clone. Since little is known about the induction of adaptive immune response by CHIP-typical mutations, it remains difficult to quantify the potential effects of such a mechanism.

In the case of persistent expansion donor-derived clones expand at constant or increasing rates over multiple years after the transplantation before they might eventually approach the maximal possible VAF. According to our simulations these dynamics can be triggered by mutation-induced changes of mutant cell kinetics and by inflammation-triggered mutated cell growth. One important difference between both mechanisms is that the involvement of inflammation is a potential therapeutic target and that an anti-inflammatory host environment might counteract the expansion of donor-derived clones. Which of the two mechanisms is more important in a clinical setting has to be clarified by further studies.

The virtual clinical trials which we have performed lead to the following testable hypotheses:

1. The expansion of donor-derived clones in response to HSCT decreases if the number of transplanted cells per kg of body weight increases, **Figure 7**.
2. The number of transplanted HSCs per kg of body weight has a higher impact on the expansion of the donor-related clones than the number of transplanted non-stem progenitors, **Figure 8**.
3. Post-transplantation cytokine support does not impact on the expansion of donor-derived clones, **Figure 7**.
4. The absolute increase of the donor VAF in the year before donation is correlated with the absolute increase of the VAF in the recipient during the first 100 days after transplantation, **Figure 9**.

These hypotheses can potentially be investigated by clinical trials or by analysis of retrospective data. Inflammatory CHIP complications and the risk that donor-derived clones evolve into overt malignancy increase with the size of the donor-derived clone. Therefore, it is clinically relevant to better understand and predict the dynamics of donor-derived clones after transplantation.

In our simulations we observe that the longer and stronger HSCs are stimulated by feedback signals in the post-transplantation period, the more prominent is the expansion of the mutated clones. The smaller the number of transplanted HSCs, the longer the recovery of the HSC population will take and the higher the donor VAF will be. A high number of wildtype cells in the transplant or a selective stimulation of wildtype HSC self-renewal and proliferation after transplantation might, therefore, reduce the expansion of mutated cells.

Our model suggest that the interplay of clonal expansion and chronic inflammation can lead to complex dynamics. Especially, whether a clone expands or declines after transplantation may also depend on the host environment, e.g., the host’s systemic inflammatory burden (**Figure 10**). Such determinants can act in favor of the host when they lead to the decline of a donor-derived clone or they can have detrimental effects by triggering expansion of a clone which has been stable in the donor. As long as the responsible inflammatory mediators remain unknown, such effects cannot be accurately predicted. Population studies show a high inter-individual variation of VAF dynamics, even if the observed clones harbor mutations in the same gene (86, 96). This finding supports the concept that clonal dynamics depend on multiple factors and not only on the gene which is mutated. A reliable modeling of mutation-specific effects, therefore, requires data sets which provide insights in a larger population of patients. Furthermore, a systematic analysis of potential disease driving mechanisms and confounders is necessary.

As all models, the models developed in this work are based on simplifying assumptions. The first assumption is that the mutated and healthy cell counts are sufficiently high to neglect stochastic effects. Since we are mostly interested in clonal expansion and not in extinction dynamics, this assumption is fulfilled. We assume that the donors’ VAF is above 0.005 which results in more than 1000 mutated HSCs in the transplant.

The considered model is formulated on the level of cell kinetics. The assumed dependencies of kinetic parameters (proliferation rates, probability of differentiation versus self-renewal) on regulatory signals are phenomenological. They have been chosen based on experimental insights, clinical observations and dynamic properties, such as robustness of the homeostatic state with respect to perturbations and timely recovery after transplantation. We do not explicitly model the detailed cellular and molecular interactions determining the cell kinetics. This choice has been made to keep the model as simple as possible and to reduce the number of unknown parameters. Stem cell niches play a key role in integrating various signals (69) and in regulating HSC properties. Since, however, the specific molecular cues are not well characterized and difficult to quantify in humans, we do not model them in detail. Instead we assume that the self-renewal probability of certain clones is an increasing or decreasing function of the inflammatory burden. Analogous simplifications are made with respect to the cytokine feedbacks. Due to their redundancy and pleiotropy (97), the effects of specific cytokines are challenging to decipher and to parameterize.

A phenomenological way to model the niche is to assume that stem cell properties depend on the current number of stem cells (70). Such a feedback can be interpreted as a local signal which is independent of systemic cues, such as mature blood cells or cytokines in the blood stream. We have considered two modifications of our proposed model which account for local signals. This did not change the qualitative dynamics of the scenarios simulated in this work (**Supplementary Figures 8–13**).

According to our simulations it is important that HSC self-renewal and the number of divisions progenitors perform before terminal differentiation increase during the reconstitution. This is in line with previous works (38, 40, 41, 98). An increase of proliferation after transplantation further accelerates the engraftment. Whether the feedback regulations mediating these changes depend on mature cell counts or immature cell counts has only a limited impact on the dynamics of the phenomena we have investigated.

The model parametrization is based on different types of data, such as equilibrium cell counts (41, 74), estimated proliferation rates (35), changes of cell kinetics in response to cytokine supplementation (78) and dynamic properties. Nevertheless, some quantities are difficult to measure or to estimate. This applies to the *in vivo* self-renewal probabilities of human hematopoietic cells, which we parameterize by calibrating the model to blood cell dynamics after transplantation. We choose this approach since self-renewal is a key determinant of the engraftment kinetics (38, 40, 41, 98). The cellular composition of the transplant, especially the content of primitive HSCs, and its dependence on the mobilization scheme (99, 100) is hard to quantify. The exact number of progenitor compartments and their precise kinetic parameters are also unknown. However, the model dynamics are very similar for different plausible choices.

Another quantity which is difficult to estimate in the human system is the fraction of transplanted cells which successfully homes to the marrow and contributes to blood cell formation versus the fraction of transplanted cells which is cleared without contributing. If the inter-individual variation of this quantity is high, it might hamper the observation of straightforward relationships between the transplanted cell dose and the expansion of donor-derived clones. Furthermore, donor and recipient variables not considered in the model might impact on the magnitude of the observed effects.

The current work only considers the dynamics of donor-derived clones after transplantation. The acquisition of additional mutations and the transformation into donor-derived malignancy are not modeled. The acquisition of *de novo* mutations is a stochastic event and, therefore, difficult to predict. Accounting for stochastic events significantly increases the complexity and computational costs of the model (51, 58) and is beyond the scope of the present work. The risk for donor-derived malignancy might increase with the variant allele frequency, because the more pre-malignant cells exist, the higher the probability that at least on of them acquires a transforming mutation. Computer simulations suggest that the self-renewal probability of malignant stem cells increases during disease evolution (44, 48, 58). A concomitant increase of proliferation rates accelerates disease progression. A probable scenario for the development of malignancy is the expansion of donor- derived clones followed by the acquisition of new mutations which increase the stem cell self-renewal probability and proliferation rate.

If clinically confirmed, our results suggest that an increased dose of transplanted HSC can reduce the expansion of donor-derived clones. Such a recommendation could be well introduced in clinical practice, since there is evidence that high transplant doses are beneficial in various respects (41, 101, 102). Which minimal HSC dose should be recommended has to be specified in clinical trials. An increase of the transplant dose potentially comes with an increase of the number of non-stem progenitors which might be skewed towards inflammation. Whether the engraftment of these cells, even if temporary, might have negative effects for the host has to be studied in clinical trials.

In summary, the quantitative models developed in this work suggest that the dynamics of donor-derived clones after allogenic HSCT emerge from the interplay of different mechanisms such as alternated response of mutated cells to feedback signals, direct effects of mutations on cell kinetics and chronic inflammation. The number of HSCs transplanted per kg of body weight might impact on the expansion of donor-derived clones and warrants further clinical investigation.

## Data Availability

The original contributions presented in the study are included in the article/**Supplementary Material**. Further inquiries can be directed to the corresponding author.
