# Supplementary material for: Stem cell graft dose and composition could impact on the expansion of donor-derived clones after allogeneic hematopoietic stem cell transplantation – a virtual clinical trial
Source: Front Immunol. 2024 Dec 16;15:1321336. doi: 10.3389/fimmu.2024.1321336 (PMC11682905; doi:10.3389/fimmu.2024.1321336)
Supplement: Supplementary file 1 [file DataSheet1.pdf]

Supplementary Information: Stem cell graft dose and composition could impact on the expansion of donor-derived clones after allogeneic hematopoietic stem cell transplantation - a virtual clinical trial

T. Stiehl

Contents

1 Model Derivation **2**
1.1 Model of granulopoiesis . . . 2
1.1.1 Cell population dynamics . . . 2
1.1.2 Feedbacks . . . 3
1.2 Model of CHIP . . . 4
1.2.1 Compartment structure . . . 4
1.2.2 Response of mutated cells to feedback signals . . . 4
1.2.3 CHIP-Model . . . 6
1.3 Growth factor support . . . 8
1.4 Niche effects . . . 10
1.4.1 Modification 1: HSC self-renewal is regulated by a local feedback. . . . 10
1.4.2 Modification 2: HSC self-renewal and proliferation are regulated by a local feedback. . . 11
1.5 CHIP-related chronic inflammation: Model 1 . . . 11
1.6 CHIP-related chronic inflammation: Model 2 . . . 13
1.7 Model with two clones . . . 14

2 Parameterization **16**
2.1 Blood cell formation . . . 16
2.2 HSPC grafts . . . 19
2.3 Cytokine support . . . 19
2.4 In silico trials . . . 20
2.4.1 Wildtype cell parameters, equilibria and feedback response . . . 20
2.4.2 Mutated cell parameters for saturated expansion . . . 21
2.4.3 Mutated cell parameters for persistent expansion . . . 22

3 Parameter sets used in the figures **22**

4 Additional Figures **31**

1 Model Derivation

1.1 Model of granulopoiesis

1.1.1 Cell population dynamics

The structure of the model is summarized in SFig. 1 (A). Denote the number of hematopoietic stem cells per kg of body weight at time t as $c_1(t)$, the stem cell proliferation rate as $p_1(t)$ and the self-renewal probability (fraction of self-renewal) as $a_1(t)$.

Then, the flux of HSCs to mitosis at time t is given by $c_1(t)p_1(t)$. Neglecting the duration of mitosis the influx of daughter cells at time t is given by $2p_1(t)c_1(t)$. The fraction $a_1(t)$ of the daughter cells belongs to the stem cell compartment (self-renewal) and the fraction $1 - a_1(t)$ differentiates into progenitors.

This results in the following equation for HSCs [25]:

$$\frac{d}{dt}c_1(t) = (2a_1(t) - 1)p_1(t)c_1(t) \tag{1}$$

The time dependence of p_1 and a_1 will be specified in Section 1.1.2.

We consider $N > 0$ subsequent differentiation stages between the stem cell and the mature cell stage. These stages correspond to progenitor and precursor cells. The number of progenitor/precursor cells of stage i at time t per kg of body weight is denoted by $c_2^i(t)$ $(1 \leq i \leq N)$ and their respective proliferation rates and self-renewal probabilities as $p_2^i(t)$ and $a_2^i(t)$. Progenitor and precursor cell population dynamics are given by the following equations:

$$\begin{aligned}
\frac{d}{dt}c_2^1(t) &= 2(1 - a_1(t))p_1(t)c_1(t) + (2a_2^1(t) - 1)p_2^1(t)c_2^1(t) \\
\ldots &= \ldots \\
\frac{d}{dt}c_2^i(t) &= 2(1 - a_2^{i-1}(t))p_2^{i-1}(t)c_2^{i-1}(t) + (2a_2^i(t) - 1)p_2^i(t)c_2^i(t) \qquad (2 \leq i \leq N-1) \\
\ldots &= \ldots \\
\frac{d}{dt}c_2^N(t) &= 2(1 - a_2^{N-1}(t))p_2^{N-1}(t)c_2^{N-1}(t) + (2a_2^N(t) - 1)p_2^N(t)c_2^N(t)
\end{aligned} \tag{2}$$

The term $2(1 - a_1(t))p_1(t)c_1(t)$ describes the flux from the stem cell compartment to the most immature progenitor compartment due to differentiation. Analogously the term $2(1 - a_2^{i-1}(t))p_2^{i-1}(t)c_2^{i-1}(t)$ describes the flux from progenitor compartment $i - 1$ to progenitor compartment i due to differentiation. The time dependence of p_2^i and a_2^i will be specified in Section 1.1.2.

We denote by $c_3(t)$ the number of neutrophil granulocytes (PMN) per kg of body weight at time t. We assume that mature neutrophils are cleared at rate $d_3 > 0$. Since mature neutrophils do not divide, we obtain the following equation:

$$\frac{d}{dt}c_3(t) = 2(1 - a_2^N(t))p_2^N(t)c_2^N(t) - d_3c_3(t). \tag{3}$$

1.1.2 Feedbacks

The hematopoietic cells are subjected to feedback regulations to adapt their dynamics to the current need of the organism. A major feedback signal of neutrophil formation is G-CSF, which is produced at (approximately) constant rates and eliminated by mature neutrophils [21] and by cell-independent mechanisms. Denote the G-CSF concentration at time t by $S(t)$. Its time evolution is given by

$$\frac{d}{dt}S(t) = \alpha - \beta S(t) - \gamma c_3(t)S(t).$$

Since growth factor kinetics evolve fast compared to cell kinetics [10, 18, 23, 37], we apply a quasi-steady state approximation as in [24, 25] and obtain

$$S(t) = \frac{\alpha}{\beta + \gamma c_3(t)}.$$

If we divide $S(t)$ by its maximum α/β we obtain the signal $s(t)$ which assumes values in $(0, 1]$ and is given by a first order Hill function:

$$s(t) = \frac{1}{1 + kc_3(t)}. \tag{4}$$

Hill functions are widely used to model feedbacks in the hematopoietic system [8, 12, 13, 25, 32–36].

It is known [38] that proliferation rates increase if there is a shortage of mature cells. Therefore, we set

$$p_1(t) = \min(1, p_1^{max}s_0(t)) \tag{5}$$

$$p_2^i(t) = \min(1, p_2^{i,max}s_1(t)) \qquad (1 \leq i \leq N) \tag{6}$$

with

$$s_0(t) = \frac{1}{1 + k_0c_3(t)} \tag{7}$$

$$s_1(t) = \frac{1}{1 + k_1c_3(t)} \tag{8}$$

and $p_1^{max},\ p_2^{i,max} > 0$. We use the min function to avoid that cells divide more often than once per day [11], which is considered an upper limit of division frequencies for eukaryotic cells. The ratio of the proliferation rate under maximal stimulation and the steady state proliferation rate depends on k_0 and k_1 and the equilib-

rium cell counts. We introduce two signals s_0 and s_1 to allow this ratio to be different for HSCs and HPCs. According to experimental observations progenitors increase their proliferation rates approximately 4-fold under stress conditions [38]. Since a 4-fold increase of HSC proliferation would not be sufficient to explain the recovery of HSC counts after AML therapy observed in [42, 46], we assume that HSC proliferation rate can increase more than 4-fold.

Similarly, there is evidence that HSC self-renewal increases during hematopoietic stress and that progenitors perform more divisions before terminal differentiation [25, 33, 34]. Therefore, we set

$$a_1(t) = a_1^{max} s_2(t) \tag{9}$$

$$a_2^i(t) = a_2^{i,max} s_2(t) \qquad (1 \leq i \leq N) \tag{10}$$

with

$$s_2(t) = \frac{1}{1 + k_2 c_3(t)} \tag{11}$$

and $1 > a_1^{max} > 0.5$, $0.5 > a_2^{i,max} > 0$. We introduce s_2 to account for the fact that proliferation rates and self-renewal probabilities may exhibit different relative changes upon stimulation. To ensure that non-stem cells differentiate after a finite number of divisions, we impose $a_2^{i,max} < 0.5$.

1.2 Model of CHIP

1.2.1 Compartment structure

We model clonal hematopoiesis by assuming competition of wildtype and mutated cells. At the moment we consider only one mutated clone. Mutated cells follow the same compartmental organization as wildtype cells and are subjected to the same processes as wildtype cells, namely proliferation, self-renewal, differentiation, clearance. All quantities related to mutated cells are denoted by ^{M}. The model is visualized in SFig. 1 (B).

1.2.2 Response of mutated cells to feedback signals

We consider the possibility that mutated cells respond more sensitively to perturbations of the feedback signals in the vicinity of their homeostatic values. Therefore, the linear dependencies of wildtype cell properties on feedback signals are replaced by different functional relations. We set

$$f(x, \sigma, \bar{s}) = \min(\max(0, (x - \bar{s})\sigma + \bar{s}), 1) \qquad \text{with } 0 < \bar{s} < 1, \ \sigma > 0. \tag{12}$$

We note that $0 \leq f(s, \sigma, \bar{s}) \leq 1$ and that $f(\bar{s}, \sigma, \bar{s}) = \bar{s}$. Furthermore $f(x, 1, \bar{s}) = x$ if $0 \leq x \leq 1$. The shape of f is depicted in SFig. 2

Let $\bar{s}_0$, $\bar{s}_1$ and $\bar{s}_2$ denote the values of the feedback signals s_0, s_1, s_2 in the homeostatic equilibrium. We set

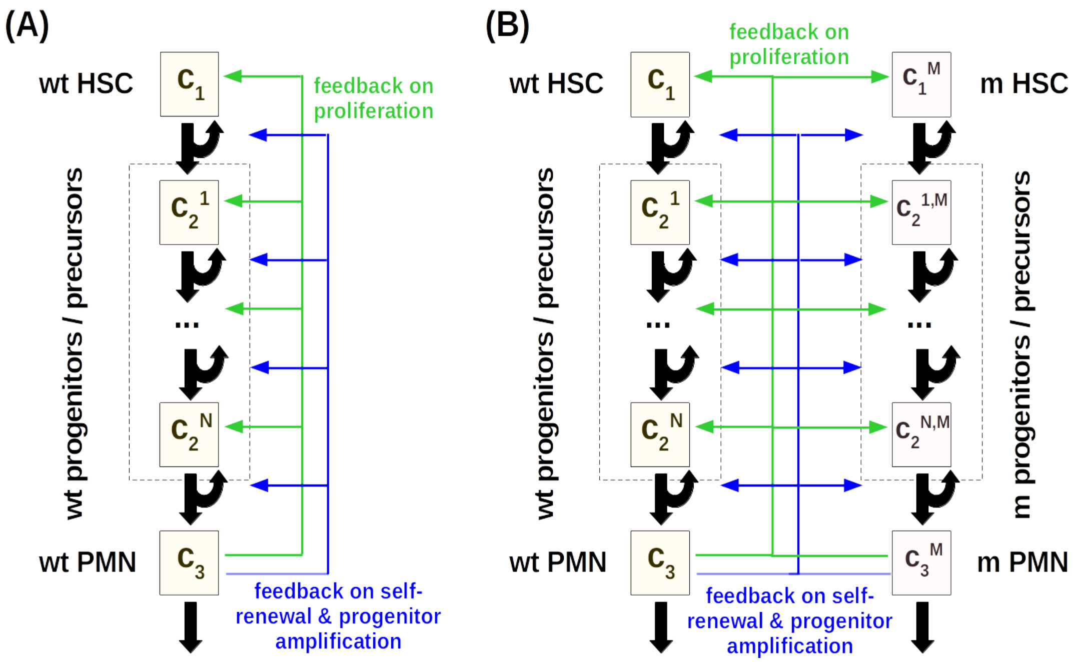

Supplementary Figure 1: **Overview of the model of blood cell formation.** (A) Submodel of healthy hematopoiesis. Proliferation, self-renewal and progenitor amplification depend on feedback signals. HSC: c_1, progenitors/precursors: c_2^1, ..., c_2^N, mature neutrophils (PMN = polymorphnuclear neutrophils): c_3. (B) Competition of wildtype and mutated cells, which depend on the same feedback mechanisms. Mutated HSCs: c_1^M, mutated progenitors/precursors: $c_2^{1,M}$, ..., $c_2^{N,M}$, mutated mature neutrophils: c_3^M, wt: wildtype, m: mutated.

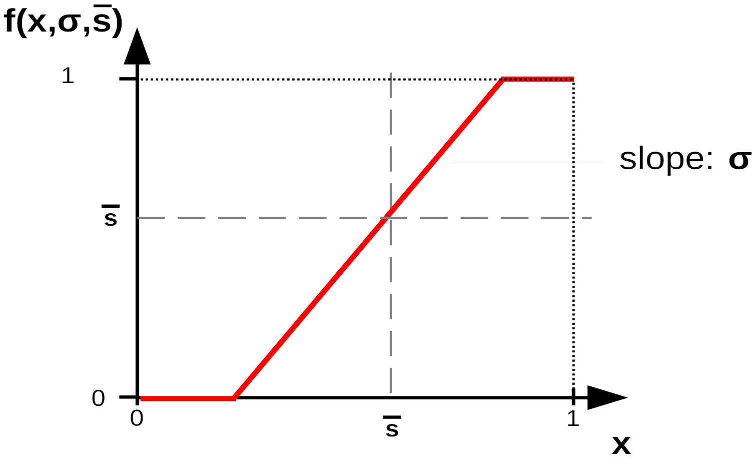

Supplementary Figure 2: **Shape of the function f.**

$$p_1^M(t) = \min\left(1, p_1^{max,M} f(s_0(t), \sigma_{p_1}, \bar{s}_0)\right) \tag{13}$$
$$p_2^{i,M(t)} = \min\left(1, p_2^{i,max,M} f(s_1(t), \sigma_{p_2}, \bar{s}_1)\right) \qquad (1 \leq i \leq N) \tag{14}$$
$$a_1^M(t) = a_1^{max,M} f(s_2(t), \sigma_{a_1}, \bar{s}_2) \tag{15}$$
$$a_2^{i,M(t)} = a_2^{i,max,M} f(s_2(t), \sigma_{a_2}, \bar{s}_2) \qquad (1 \leq i \leq N). \tag{16}$$

This implies that in the homeostatic equilibrium the mutated cells and the wildtype cells are equally stimulated by the feedback signals since $\bar{s}_i = f(\bar{s}_i, \sigma, \bar{s}_i)$. However, if the feedback signals deviate from their homeostatic values the stimulation of the mutated cells differs from the stimulation of the wildtype cells.

Especially if $p_1^{max,M} = p_1^{max}$, $p_2^{i,max,M} = p_2^{i,max}$, $a_1^{max,M} = a_1^{max}$ and $a_2^{i,max,M} = a_2^{i,max}$, $d_3 = d_3^M$ mutated and wildtype have identical properties under equilibrium conditions but show different responses if the system is perturbed. This setting allows to account for scenarios where mutated cells have a competitive advantage under hematopoietic stress or chronic inflammation.

1.2.3 CHIP-Model

Accounting for the assumptions introduced above the full model is as follows.

$$
\begin{aligned}
\frac{d}{dt}c_1(t) &= (2a_1(t)-1)p_1(t)c_1(t) \\
\frac{d}{dt}c_2^1(t) &= 2(1-a_1(t))p_1(t)c_1(t) + (2a_2^1(t)-1)p_2^1(t)c_2^1(t) \\
\ldots &= \ldots \\
\frac{d}{dt}c_2^i(t) &= 2(1-a_2^{i-1}(t))p_2^{i-1}(t)c_2^{i-1}(t) + (2a_2^i(t)-1)p_2^i(t)c_2^i(t) && (2 \le i \le N-1) \\
\ldots &= \ldots \\
\frac{d}{dt}c_2^N(t) &= 2(1-a_2^{N-1}(t))p_2^{N-1}(t)c_2^{N-1}(t) + (2a_2^N(t)-1)p_2^N(t)c_2^N(t) \\
\frac{d}{dt}c_3(t) &= 2(1-a_2^N(t))p_2^N(t)c_2^N(t) - d_3c_3(t) \\
\frac{d}{dt}c_1^M(t) &= (2a_1^M(t)-1)p_1^M(t)c_1^M(t) \\
\frac{d}{dt}c_2^{1,M}(t) &= 2(1-a_1^M(t))p_1^M(t)c_1^M(t) + (2a_2^{1,M}(t)-1)p_2^{1,M}(t)c_2^{1,M}(t) \\
\ldots &= \ldots \\
\frac{d}{dt}c_2^{i,M}(t) &= 2(1-a_2^{i-1,M}(t))p_2^{i-1,M}(t)c_2^{i-1,M}(t) + (2a_2^{i,M}(t)-1)p_2^{i,M}(t)c_2^{i,M}(t) && (2 \le i \le N-1) \\
\ldots &= \ldots \\
\frac{d}{dt}c_2^{N,M}(t) &= 2(1-a_2^{N-1,M}(t))p_2^{N-1,M}(t)c_2^{N-1,M}(t) + (2a_2^{N,M}(t)-1)p_2^{N,M}(t)c_2^{N,M}(t) \\
\frac{d}{dt}c_3^M(t) &= 2(1-a_2^{N,M}(t))p_2^{N,M}(t)c_2^{N,M}(t) - d_3^Mc_3^M(t)
\end{aligned}
$$

$$
\begin{aligned}
p_1(t) &= \min(1, p_1^{max} s_0(t)) \\
p_2^i(t) &= \min(1, p_2^{i,max} s_1(t)) && (1 \le i \le N) \\
p_1^M(t) &= \min\left(1, p_1^{max,M} f(s_0(t), \sigma_{p_1}, \bar{s}_0)\right) \\
p_2^{i,M}(t) &= \min\left(1, p_2^{i,max,M} f(s_1(t), \sigma_{p_2}, \bar{s}_1)\right) && (1 \le i \le N) \\
a_1(t) &= a_1^{max} s_2(t) \\
a_2^i(t) &= a_2^{i,max} s_2(t) && (1 \le i \le N) \\
a_1^M(t) &= a_1^{max,M} f\left(s_2(t), \sigma_{a_1}, \frac{1}{2a_1^{max}}\right) \\
a_2^{i,M}(t) &= a_2^{i,max,M} f\left(s_2(t), \sigma_{a_2}, \frac{1}{2a_1^{max}}\right) && (1 \le i \le N) \\
s_0(t) &= \frac{1}{1 + k_0(c_3(t) + c_3^M(t))} \\
s_1(t) &= \frac{1}{1 + k_1(c_3(t) + c_3^M(t))} \\
s_2(t) &= \frac{1}{1 + k_2(c_3(t) + c_3^M(t))} \\
\bar{s}_0 &= \frac{k_2}{k_2 + k_0(2a_1^{max} - 1)} \\
\bar{s}_1 &= \frac{k_2}{k_2 + k_1(2a_1^{max} - 1)}
\end{aligned}
\tag{17}
$$

supplemented by non-negative initial conditions.

1.3 Growth factor support

We use a two-compartment submodel to account for growth factor support. The model is inspired by the kinetics of pegfilgrastim which is injected subcutaneously. The action of the drug is assumed to be proportional to its availability in the plasma. We model the injection as an instantaneous event. The drug is assumed to leave the subcutaneous depot proportional to its concentration. The concentration of drug in the subcutaneous compartment is denoted as g_{depot}, the transition rate to the plasma by k_g, the initial amount by g_0.

$$
\frac{d}{dt} g_{depot} = -k_g \cdot g_{depot} \tag{18}
$$

$$
g_{depot}(t_0) = g_0 \tag{19}
$$

We assume that the drug is cleared from the plasma proportional to its concentration at rate d_g. The constant κ_{dil} accounts for the dilution when the drug transits from the first to the second compartment. This leads to the following equation

$$\frac{d}{dt}g_{blood} = k_g \cdot g_{depot} \cdot \kappa_{dil} - d_g \cdot g_{blood} \tag{20}$$

$$g_{blood}(t_0) = 0. \tag{21}$$

The latter equation simplifies to

$$\frac{d}{dt}g_{blood} = k_g \cdot \kappa_{dil} \cdot g_0 \cdot e^{-k_g(t-t_0)} - d_g \cdot g_{blood} \tag{22}$$

$$g_{blood}(t_0) = 0. \tag{23}$$

We assume that the drug effect on HSCs and HPCs is proportional to the plasma concentration and given by $\eta_g \cdot g_{blood}$, which leads to

$$\frac{d}{dt}(\eta_g \cdot g_{blood}) = k_g \cdot \eta_g \cdot \kappa_{dil} \cdot g_0 \cdot e^{-k_g(t-t_0)} - d_g \cdot g_{blood} \cdot \eta_g \tag{24}$$

$$g_{blood}(t_0) = 0. \tag{25}$$

We aggregate parameters and rewrite the ODE for $g_{effect} := \eta_g \cdot g_{blood}$ as

$$\frac{d}{dt}g_{effect} = G_0 \cdot k_g \cdot e^{-k_g(t-t_0)} - D_g \cdot g_{effect} \tag{26}$$

$$g_{effect}(t_0) = 0 \tag{27}$$

and adjust the parameters G_0, k_g, D_g.

To simulate cytokine support we set:

$$s_0(t) = \min\left(\frac{1}{1 + k_0(c_3(t) + c_3^M(t))} + g_{effect}, \hat{S}\right)$$

$$s_1(t) = \min\left(\frac{1}{1 + k_1(c_3(t) + c_3^M(t))} + g_{effect}, \hat{S}\right)$$

$$s_2(t) = \min\left(\frac{1}{1 + k_2(c_3(t) + c_3^M(t))} + g_{effect}, \hat{S}\right)$$

$$f(x, \sigma, \bar{s}) = \min\left(\max(0, (x - \bar{s})\sigma + \bar{s}), \hat{S}\right),$$

where x stands for $s_0(t)$, $s_1(t)$ or $s_2(t)$, and $\bar{s}$ for the equilibrium values of s_0, s_1, s_2, respectively. This implies that during external cytokine support the feedback signal can be larger than under physiological conditions, however the effect of external cytokine stimulation cannot become arbitrarily large. The maximal value of the feedback signals which can be sensed by the cells is equal to $\hat{S}$. The physiological motivation for such an upper bound is the finite availability of cytokine receptors on cell surfaces.

We assure that $\hat{S}$ fulfills $a_1^{max} \cdot \hat{S} < 1$, $a_1^{max,M} \cdot \hat{S} < 1$, $a_2^{i,max} \cdot \hat{S} < 0.5$ and $a_2^{i,max,M} \cdot \hat{S} < 0.5$.

To keep the number of parameters as small as possible, the sub-model of cytokine support is relatively simple. However, due to the large interindividual differences in pegfilgrastim pharmacokinetics [2], an accurate prediction remains challenging and is beyond the scope of the conceptual models considered here.

1.4 Niche effects

Local signals from the micro-environment (stem cell niche) crucially impact on HSC regulation [26]. At the same time it is known that also systemic signals affect HSC kinetics [16, 30]. In the model introduced in the previous section (equations (17)) HSCs are regulated by systemic signals which depend on the mature cell counts. We consider two modifications of the model from the previous section to investigate how the dynamics change if we assume that HSC kinetics depend on local signals. A simple phenomenological way to account for the stem cell niche is to assume that HSC properties depend on a feedback signal which is given by a function of HSC counts. The interpretation is that, depending on the current HSC count, the niche produces signals which regulate HSC expansion.

1.4.1 Modification 1: HSC self-renewal is regulated by a local feedback.

In this version of the model we assume that HSC self-renewal is regulated by a local feedback (interpreted as the niche) and HSC proliferation by a systemic feedback. This is achieved by setting

$$\begin{aligned}
\tilde{s}_2(t) &= \frac{1}{1 + \tilde{k}_2(c_1(t) + c_1^M(t))} \\
a_1(t) &= a_1^{max} \tilde{s}_2(t) \\
a_1^M(t) &= a_1^{max,M} f\left(\tilde{s}_2(t), \sigma_{a_1}, \frac{1}{2a_1^{max}}\right)
\end{aligned}$$

in absence of cytokine support (with $f(x, \sigma, \bar{s}) = \min(\max(0, (x - \bar{s})\sigma + \bar{s}), 1))$ and

$$\begin{aligned}
\tilde{s}_2(t) &= \min\left(\frac{1}{1 + \tilde{k}_2(c_1(t) + c_1^M(t))} + g_{effect}, \hat{S}\right) \\
a_1(t) &= a_1^{max} \tilde{s}_2(t) \\
a_1^M(t) &= a_1^{max,M} f\left(\tilde{s}_2(t), \sigma_{a_1}, \frac{1}{2a_1^{max}}\right)
\end{aligned}$$

in presence of cytokine support (with $f(x, \sigma, \bar{s}) = \min(\max(0, (x - \bar{s})\sigma + \bar{s}), \hat{S}))$. The quantities $\hat{S}$ and g_{effect} have been introduced in Section 1.3.

1.4.2 Modification 2: HSC self-renewal and proliferation are regulated by a local feedback.

In this version of the model we assume that HSC self-renewal and proliferation are regulated by a local feedback (interpreted as the niche). This is achieved by setting

$$\begin{aligned}
\tilde{s}_2(t) &= \frac{1}{1+\tilde{k}_2(c_1(t)+c_1^M(t))} \\
a_1(t) &= a_1^{max}\tilde{s}_2(t) \\
a_1^M(t) &= a_1^{max,M} f\left(\tilde{s}_2(t), \sigma_{a_1}, \frac{1}{2a_1^{max}}\right) \\
\tilde{s}_0(t) &= \frac{1}{1+\tilde{k}_0(c_1(t)+c_1^M(t))} \\
p_1(t) &= \min(1, p_1^{max}\tilde{s}_0(t)) \\
p_1^M(t) &= \min\left(1, p_1^{max,M} f(\tilde{s}_0(t), \sigma_{p_1}, \bar{\tilde{s}}_0)\right)
\end{aligned}$$

in absence of cytokine support (with $f(x, \sigma, \bar{s}) = \min(\max(0, (x - \bar{s})\sigma + \bar{s}), 1))$ and

$$\begin{aligned}
\tilde{s}_2(t) &= \min\left(\frac{1}{1+\tilde{k}_2(c_1(t)+c_1^M(t))} + g_{effect}, \hat{S}\right) \\
a_1(t) &= a_1^{max}\tilde{s}_2(t) \\
a_1^M(t) &= a_1^{max,M} f\left(\tilde{s}_2(t), \sigma_{a_1}, \frac{1}{2a_1^{max}}\right) \\
\tilde{s}_0(t) &= \min\left(\frac{1}{1+\tilde{k}_0(c_1(t)+c_1^M(t))} + g_{effect}, \hat{S}\right) \\
p_1(t) &= \min(1, p_1^{max}\tilde{s}_0(t)) \\
p_1^M(t) &= \min\left(1, p_1^{max,M} f(\tilde{s}_0(t), \sigma_{p_1}, \bar{\tilde{s}}_0)\right)
\end{aligned}$$

in presence of cytokine support (with $f(x, \sigma, \bar{s}) = \min(\max(0, (x-\bar{s})\sigma+\bar{s}), \hat{S}))$. The quantities $\hat{S}$ and g_{effect} have been introduced in Section 1.3. The positive constant $\bar{\tilde{s}}_0$ corresponds to the value of $\tilde{s}_0$ in the healthy equilibrium, i.e., $\bar{\tilde{s}}_0 = \frac{\tilde{k}_2}{\tilde{k}_2+\tilde{k}_0(2a_1^{max}-1)}$.

1.5 CHIP-related chronic inflammation: Model 1

In this version of the model we assume that mediators of chronic inflammation stimulate the expansion of mutated cells. Animal models and cell line experiments suggest that mutant cells might increase the systemic inflammatory burden. One mechanism for that is that white cells carrying CHIP mutations exhibit aberrant pro-inflammatory activity [7, 9, 15, 29, 41, 43, 45].

We model this mechanism by assuming that mutated mature cells, c_3^M, produce inflammatory mediators

at a constant rate δ and that these mediators are degraded proportionally to their concentration at rate ν. Denote by $\chi(t)$ the concentration of the inflammatory mediators at time t. Then it holds

$$\frac{d}{dt}\chi(t) = \delta c_3^M(t) - \nu\chi(t).$$

Cytokines usually evolve on a fast time scale, therefore, we make a quasi-steady state approximation. This results in

$$\chi(t) = \delta c_3^M(t)/\nu =: \tilde{\delta} c_3^M(t).$$

There is evidence that the inflammatory environment confers a competitive advantage to the mutated cells [1, 4]. We model this as an increase of proliferation rate and / or self-renewal fraction of mutated cells.

We assume that the impact of the inflammatory cytokines on the mutated cells saturates for high mediator concentrations and is give by a Hill function.

$$s_{infl}(t) = \alpha_{infl}\frac{\chi(t) + \iota}{1 + k_{infl}(\chi(t) + \iota)},$$

where $0 \leq \iota$ is a constant systemic inflammatory load originating from CHIP-independent sources such as atherosclerosis or autoimmune disease. We test different versions of the model. Depending on the version the inflammatory mediators affect only mutated stem cells, only mutated progenitor cells or both.

When mutated stem cell self-renewal is affected, we set

$$a_1^M(t) = a_1^{max,M}\left(\frac{1}{1 + k_2(c_3(t) + c_3^M(t))} + s_{infl}(t)\right) \tag{28}$$

When mutated progenitor cell amplification is affected, we set

$$a_2^{i,M}(t) = a_2^{i,max,M}\left(\frac{1}{1 + k_2(c_3(t) + c_3^M(t))} + s_{infl}(t)\right) \qquad (1 \leq i \leq N) \tag{29}$$

When mutated stem cell proliferation is affected, we set

$$p_1^M(t) = \min\left(p_1^{max,M}\left(\frac{1}{1 + k_0(c_3(t) + c_3^M(t))} + s_{infl}(t)\right), 1\right) \tag{30}$$

When mutated progenitor cell proliferation is affected, we set

$$p_2^{i,M}(t) = \min\left(p_2^{i,max,M}\left(\frac{1}{1 + k_1(c_3(t) + c_3^M(t))} + s_{infl}(t)\right), 1\right) \qquad (1 \leq i \leq N) \tag{31}$$

We assure that $a_1^{max}\left(\frac{\alpha_{infl}}{k_{infl}} + 1\right) < 1$, $a_1^{max,M}\left(\frac{\alpha_{infl}}{k_{infl}} + 1\right) < 1$, $a_2^{i,max}\left(\frac{\alpha_{infl}}{k_{infl}} + 1\right) < 0.5$ and $a_2^{i,max,M}\left(\frac{\alpha_{infl}}{k_{infl}} + 1\right) < 0.5$.

1.6 CHIP-related chronic inflammation: Model 2

In this version of the model we assume that mediators of chronic inflammation impair the function of wildtype HSCs. This assumption is based on observations in animal experiments which suggest that chronic inflammation drives wildtype cells into exhaustion, whereas mutated cells are resistant to the impact of chronic inflammation [3].

The production of inflammatory mediators by mutated cells is modeled as in Section 1.5, i.e., the mediator concentration at time t is given by

$$\chi(t) = \delta c_3^M(t)/\nu =: \tilde{\delta} c_3^M(t).$$

We model HSC exhaustion by a reduction of the self-renewal probability. This corresponds to a reduced stemness in the sense that HSCs tend to get lost due to differentiation. A reduced self-renewal probability results in a reduced repopulating capacity and a competitive disadvantage with eventual out-competition. The reduction in wildtype HSC self-renewal is modeled by assuming that the maximal possible self-renewal probability is a function of the inflammatory burden. We set

$$a_1(t) = a_1^{max} \cdot \left(1 - \zeta \frac{\tilde{k}_{infl}(\iota + \chi(t))}{1 + \tilde{k}_{infl}(\iota + \chi(t))}\right) \cdot \frac{1}{1 + k_2(c_3(t) + c_3^M(t))}, \tag{32}$$

where ι is a systemic background inflammation which is independent of CHIP and $\zeta \in (0, 1)$ determines by how much the self-renewal probability can be reduced in response to chronic inflammation. We assume $\tilde{k}_{infl}$ to be a positive constant.

This expression is motivated as follows: The term $\frac{1}{1+k_2(c_3(t)+c_3^M(t))}$ corresponds to the physiological feedback signal which regulates HSC self-renewal. The term $a_1^{max} \cdot \left(1 - \zeta \frac{\tilde{k}_{infl}(\iota+\chi(t))}{1+\tilde{k}_{infl}(\iota+\chi(t))}\right)$ corresponds to the self-renewal probability under maximal physiological stimulation, i.e., when the physiological feedback signal assumes the value 1. Consequently, this term implies that chronic inflammation reduces the HSC self-renewal probability.

When HPC self-renewal, i.e., the number of divisions HPCs perform before further differentiation, is reduced, we set

$$a_2^i(t) = a_2^{i,max} \cdot \left(1 - \zeta \frac{\tilde{k}_{infl}(\iota + \chi(t))}{1 + \tilde{k}_{infl}(\iota + \chi(t))}\right) \cdot \frac{1}{1 + k_2(c_3(t) + c_3^M(t))}. \tag{33}$$

If we simulate an increase of wildtype HSC proliferation in the presence of chronic inflammation we set

$$p_1(t) = \min\left(p_1^{max}\left(\frac{1}{1 + k_0(c_3(t) + c_3^M(t))} + \tilde{\zeta} \frac{\tilde{k}_{infl}(\iota + \chi(t))}{1 + \tilde{k}_{infl}(\iota + \chi(t))}\right), 1\right). \tag{34}$$

If we simulate an increase of wildtype HPC proliferation in the presence of chronic inflammation we set

$$p_2^i(t) = \min\left(p_2^{i,max}\left(\frac{1}{1+k_1(c_3(t)+c_3^M(t))} + \tilde{\zeta}\frac{\tilde{k}_{infl}(\iota+\chi(t))}{1+\tilde{k}_{infl}(\iota+\chi(t))}\right), 1\right). \tag{35}$$

By $\tilde{\zeta}$ we denote a positive constant.

1.7 Model with two clones

The proposed models can easily be generalized to account for multiple clones. We denote by $c_1(t), c_2^1(t), ..., c_2^N(t), c_3(t)$ the amount of wildtype HSC, HPC and mature cells, by $c_1^{M_1}(t), c_2^{1,M_1}(t), ..., c_2^{N,M_1}(t), c_3^{M_1}(t)$ the amount of HSC, HPC and mature cells belonging to clone 1 and by $c_1^{M_2}(t), c_2^{1,M_2}(t), ..., c_2^{N,M_2}(t), c_3^{M_2}(t)$ the respective cell counts belonging to clone 2. The extension of the model provided in the system of equations (17) for two mutated clones is given by

$$
\begin{aligned}
\frac{d}{dt}c_1(t) &= (2a_1(t)-1)p_1(t)c_1(t) \\
\frac{d}{dt}c_2^1(t) &= 2(1-a_1(t))p_1(t)c_1(t) + (2a_2^1(t)-1)p_2^1(t)c_2^1(t) \\
\dots &= \dots \\
\frac{d}{dt}c_2^i(t) &= 2(1-a_2^{i-1}(t))p_2^{i-1}(t)c_2^{i-1}(t) + (2a_2^i(t)-1)p_2^i(t)c_2^i(t) && (2 \le i \le N-1) \\
\dots &= \dots \\
\frac{d}{dt}c_2^N(t) &= 2(1-a_2^{N-1}(t))p_2^{N-1}(t)c_2^{N-1}(t) + (2a_2^N(t)-1)p_2^N(t)c_2^N(t) \\
\frac{d}{dt}c_3(t) &= 2(1-a_2^N(t))p_2^N(t)c_2^N(t) - d_3c_3(t) \\
\frac{d}{dt}c_1^{M_1}(t) &= (2a_1^{M_1}(t)-1)p_1^{M_1}(t)c_1^{M_1}(t) \\
\frac{d}{dt}c_2^{1,M_1}(t) &= 2(1-a_1^{M_1}(t))p_1^{M_1}(t)c_1^{M_1}(t) + (2a_2^{1,M_1}(t)-1)p_2^{1,M_1}(t)c_2^{1,M_1}(t) \\
\dots &= \dots \\
\frac{d}{dt}c_2^{i,M_1}(t) &= 2(1-a_2^{i-1,M_1}(t))p_2^{i-1,M_1}(t)c_2^{i-1,M_1}(t) + (2a_2^{i,M_1}(t)-1)p_2^{i,M_1}(t)c_2^{i,M_1}(t) && (2 \le i \le N-1) \\
\dots &= \dots \\
\frac{d}{dt}c_2^{N,M_1}(t) &= 2(1-a_2^{N-1,M_1}(t))p_2^{N-1,M_1}(t)c_2^{N-1,M_1}(t) + (2a_2^{N,M_1}(t)-1)p_2^{N,M_1}(t)c_2^{N,M_1}(t) \\
\frac{d}{dt}c_3^{M_1}(t) &= 2(1-a_2^{N,M_1}(t))p_2^{N,M_1}(t)c_2^{N,M_1}(t) - d_3^{M_1}c_3^{M_1}(t) \\
\frac{d}{dt}c_1^{M_2}(t) &= (2a_1^{M_2}(t)-1)p_1^{M_2}(t)c_1^{M_2}(t) \\
\frac{d}{dt}c_2^{1,M_2}(t) &= 2(1-a_1^{M_2}(t))p_1^{M_2}(t)c_1^{M_2}(t) + (2a_2^{1,M_2}(t)-1)p_2^{1,M_2}(t)c_2^{1,M_2}(t) \\
\dots &= \dots \\
\frac{d}{dt}c_2^{i,M_2}(t) &= 2(1-a_2^{i-1,M_2}(t))p_2^{i-1,M_2}(t)c_2^{i-1,M_2}(t) + (2a_2^{i,M_2}(t)-1)p_2^{i,M_2}(t)c_2^{i,M_2}(t) && (2 \le i \le N-1) \\
\dots &= \dots \\
\frac{d}{dt}c_2^{N,M_2}(t) &= 2(1-a_2^{N-1,M_2}(t))p_2^{N-1,M_2}(t)c_2^{N-1,M_2}(t) + (2a_2^{N,M_2}(t)-1)p_2^{N,M_2}(t)c_2^{N,M_2}(t) \\
\frac{d}{dt}c_3^{M_2}(t) &= 2(1-a_2^{N,M_2}(t))p_2^{N,M_2}(t)c_2^{N,M_2}(t) - d_3^{M_2}c_3^{M_2}(t)
\end{aligned}
$$

$$
\begin{aligned}
p_1(t) &= \min(1, p_1^{max} s_0(t)) \\
p_2^i(t) &= \min(1, p_2^{i,max} s_1(t)) && (1 \le i \le N) \\
p_1^{M_1}(t) &= \min\left(1, p_1^{max,M_1} f(s_0(t), \sigma_{p_1}^{M_1}, \bar{s}_0)\right) \\
p_2^{i,M_1}(t) &= \min\left(1, p_2^{i,max,M_1} f(s_1(t), \sigma_{p_2}^{M_1}, \bar{s}_1)\right) && (1 \le i \le N) \\
p_1^{M_2}(t) &= \min\left(1, p_1^{max,M_2} f(s_0(t), \sigma_{p_1}^{M_2} \bar{s}_0)\right) \\
p_2^{i,M_2}(t) &= \min\left(1, p_2^{i,max,M_2} f(s_1(t), \sigma_{p_2}^{M_2}, \bar{s}_1)\right) && (1 \le i \le N) \\
a_1(t) &= a_1^{max} s_2(t) \\
a_2^i(t) &= a_2^{i,max} s_2(t) && (1 \le i \le N) \\
a_1^{M_1}(t) &= a_1^{max,M_1} f\left(s_2(t), \sigma_{a_1}^{M_1}, \frac{1}{2a_1^{max}}\right) \\
a_2^{i,M_1}(t) &= a_2^{i,max,M_1} f\left(s_2(t), \sigma_{a_2}^{M_1}, \frac{1}{2a_1^{max}}\right) && (1 \le i \le N) \\
a_1^{M_2}(t) &= a_1^{max,M_2} f\left(s_2(t), \sigma_{a_1}^{M_2}, \frac{1}{2a_1^{max}}\right) \\
a_2^{i,M_2}(t) &= a_2^{i,max,M_2} f\left(s_2(t), \sigma_{a_2}^{M_2}, \frac{1}{2a_1^{max}}\right) && (1 \le i \le N) \\
s_0(t) &= \frac{1}{1 + k_0(c_3(t) + c_3^{M_1}(t) + c_3^{M_2}(t))} \\
s_1(t) &= \frac{1}{1 + k_1(c_3(t) + c_3^{M_1}(t) + c_3^{M_2}(t))} \\
s_2(t) &= \frac{1}{1 + k_2(c_3(t) + c_3^{M_1}(t) + c_3^{M_2}(t))} \\
\bar{s}_0 &= \frac{k_2}{k_2 + k_0(2a_1^{max} - 1)} \\
\bar{s}_1 &= \frac{k_2}{k_2 + k_1(2a_1^{max} - 1)}
\end{aligned}
\tag{36}
$$

Parameters defining properties of clone 1 have superscript ^{M_1}, parameters defining properties of clone 2 have superscript ^{M_2}.

2 Parameterization

2.1 Blood cell formation

For the transformation of units we assume a body weigth of 80 kg and a blood volume of 5 liters. We denote quantities in the physiologial equilibrium by a bar, e.g., $\bar{c}_1$ refers to the HSC count in healthy homeostasis. Lee-Six et al. [22] report that 50000 - 200000 HSCs are actively contributing to white blood cell formation at any point in time in humans, 100000 HSC correspond to 12500 HSCs per kg of body weight. Therefore,

we set

$$\bar{c}_1 = 12500/kg.$$

We set

$$\bar{c}_3 = 31250000/kg,$$

corresponding to 5 neutrophils per nanoliter of blood, a value within the reference range [28].

It has been reported that HSCs divide between once per 23 weeks and once per 67 weeks [31] or between once per 25 and once per 50 weeks [6]. We assume that stem cells divide twice per year, which is in line with these findings, i.e.,

$$\bar{p}_1 = 0.0054/day.$$

In agreement with [5] we set

$$d_3 = 2.3/day.$$

The daily loss of neutrophils from periperal blood ξ_3 is then given by

$$\xi_3 = d_3\bar{c}_3 = 718750000/day/kg.$$

In equilibrium this equals the daily influx of neutrophils from the most mature progenitor compartment.

In healthy equilibrium it holds $\bar{a}_1 = 0.5$, therefore, the influx into the most immmature progenitor compartment c_2^1, denoted by ξ_1, is given by

$$\xi_1 = 2(1-\bar{a}_1)\bar{p}_1\bar{c}_1 = \bar{p}_1\bar{c}_1 = 67.5/day/kg.$$

Therefore, the amplification factor η achieved due to progenitor division is given by

$$\eta = \frac{\xi_3}{\xi_1} \approx 1.06 \cdot 10^7 \approx 2^{23.3},$$

implying that cells perform in average 23 to 24 divisions in the progenitor compartments. The last 4-8 of these divisions are performed from the myeloblast stage and onward [19].

We want to divide the progenitor compartment into subcompartments to account for the fact that proliferation rates increase with increasing maturity. For this we choose

$$N = 15.$$

We assume that cells divide in average the same number of times in each subcompartment. This implies that cell numbers get amplified by a factor of approximately 3 times ($2.94 = \eta^{1/N}$) in each subcompartment. Other choices of N lead to very similar model dynamics. For this choice of N the minimal number of divisions cells perform in the progenitor compartments (i.e., $a_2^i = 0$) equals 15, which is plausible since after 15 divisions, according to the calculations above, cells adopt a myeloblast phenotype under equilibrium conditions.

Let κ be the influx to one of the progenitor compartments c and $\bar{a}$ the steady state self-renewal probability

and $\bar{p}$ the steady state proliferation rate in this compartment. Then it holds

$$0 = \frac{d}{dt}c = \kappa + (2\bar{a} - 1)\bar{p}\bar{c} \Leftrightarrow \bar{c} = \frac{\kappa}{(1 - 2\bar{a})\bar{p}}$$

The steady state outflux λ of the compartment c is then given by $\lambda = 2(1 - \bar{a})\bar{p}\bar{c}$. The ratio of outflux to influx is then given by

$$\frac{\lambda}{\kappa} = \frac{2(1 - \bar{a})}{(1 - 2\bar{a})}.$$

This equation relates the amplification of the cell number in the given compartment to the self-renewal probability $\bar{a}$. Setting $\frac{2(1-\bar{a})}{(1-2\bar{a})} = \eta^{1/N}$ implies $\bar{a} = 0.24239$. Since in steady $\bar{s}_2 = 1/(2a_1^{max})$, we set

$$a_2^{i,max} = 0.24239/\bar{s}_2.$$

Furthermore, it holds

$$k_2 = \frac{2a_1 - 1}{\bar{c}_3}.$$

The most mature neutrophil precursors divide approximately once per day [14]. We assume that proliferation rates increase in equal steps with increasing maturation, i.e.,

$$\bar{p}_2^i = \bar{p}_1 + i\Delta p \qquad (1 \le i \le N), \Delta p := \frac{1/day - \bar{p}_1}{N}.$$

There is evidence that progenitor proliferation rates can increase approximately by a factor of 4 due to cytokine stimulation [38], implying $\bar{s}_1 = 0.25$ and, therefore,

$$k_1 = 3/\bar{c}_3 = 9.6 \cdot 10^{-9} kg,$$

and

$$p_2^{i,max} = 4\bar{p}_2^i.$$

Since a 4-fold increase of HSC proliferation would not be sufficient to explain the recovery of HSC counts after AML therapy observed in [42], we set

$$p_1^{max} = 8\bar{p}_1,$$

which implies $\bar{s}_0 = \frac{1}{8}$ and

$$k_0 = \frac{7}{\bar{c}_3} = 2.24 \cdot 10^{-8} kg.$$

To avoid that cells divide more often than once per day [11], which is considered an upper limit of division frequencies for eukaryotic cells, we set

$$p_2^i(t) = \min(1, p_2^{i,max} s_1(t)).$$

To find a realistic value for a_1^{max}, we simulate a HSCT using a graft corresponding to $4.7 \cdot 10^6$ CD34 $^+$ cells per kg of body weight. The HSC frequency in the graft is difficult to quantify. Assuming that $\approx 10\%$ of LTC-ICs are HSCs, we consider 4000 HSCs per kg of body weight, in analogy to [34]. The graft composition

is as defined in Section 2.2. For

$$a_1^{max} = 0.65,$$

we obtain realistic neutrophil engraftment dynamics, see Fig. 1 (B) of the main text. The time evolution of all subpopulations is shown in SFig 4 and SFig 5.
This results in the following parameters:

$$p_1^{max} = 0.0432/day,$$

$$p_2^{max} = [0.29, 0.55, 0.82, 1.08, 1.35, 1.61, 1.88, 2.14, 2.41, 2.67, 2.94, 3.20, 3.47, 3.73, 4.00]^T/day,$$

$$k_2 = 9.6 \cdot 10^{-10} kg,$$
$$a_2^{i,max} = 0.3151.$$

2.2 HSPC grafts

Based on [34] we assume that a $CD34^+$ PBSC graft of $5 \cdot 10^6$ cells per kg of body weight contains 4000 primitive HSCs per kg of body weight and $9.7 \cdot 10^5$ HPC (frequency of CFU G: 43.2/1000, frequency of CFU GM: 150/1000) per kg of body weight contributing to neutrophil formation.

We assume that the HPCs are are distributed over the compartments according to their steady state ratios. This results in the following initial condition for transplantation:

$$c_0 = h_{HPC} \cdot [0.0400 \cdot h_{HSC}/h_{HPC}, 0.0005, 0.0007, 0.0014, 0.0032, 0.0075, 0.0185, 0.0468, 0.1207, 0.3164, 0.8392, 2.2485, 6.0742, 0, 0, 0, 0]^T \cdot 10^5/kg,$$

where $0 < h_{HSC} \leq 1$ is the faction of HSCs homing to the bone marrow and $0 < h_{HPC} \leq 1$ is the faction of HPCs homing to the bone marrow. The counts of $CD34^-$ cell types are set to zero. We observe that the transplant is enriched for primitive HSCs.

2.3 Cytokine support

For $k_g = 0.65/day$ and $D_g = 0.4/day$ the maximum plasma concentration is reached after approx. 2 days which is in the reported range of 16 to 120 hours [2], see Fig. 3. Furthermore, the half-maximal concentration is measured 3-4 days after the maximum. This is in line with observations in individuals after chemotherapy [44]. Cytokine support is mostly provided within the first 7 days after transplantation [20]. We assume the injection to take place 3 days after transplantation. For $G_0 = 0.6$ we observe realistic accelerations of neutrophil engraftment of 3 days (Fig. 1 of the main text, SFig 5) as described in large patient cohorts receiving G-CSF [20]. Pegfilgrastim seems to have a comparable effect to G-CSF at least in an autologous setting [39].

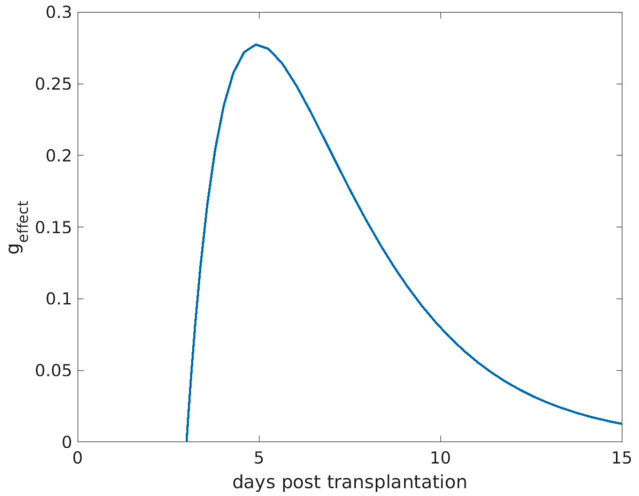

Supplementary Figure 3: **Kinetics of externally administered growth factors.** The quantity g_{effect} corresponds to the increment of the feedback signals due to pegfilgrastim administration. The cytokine is administered at day 3 post transplantation.

2.4 In silico trials

2.4.1 Wildtype cell parameters, equilibria and feedback response

Parameters of individual virtual patients are obtained by random perturbation of the parameters specified in Section 2.1. The random perturbations were drawn from uniform distributions. By " ˆ " we denote the perturbed parameters. HSC and HPC proliferation rates, stem cell self-renewal probabilities, mature cell counts, stem cell counts and feedback responses are perturbed in the range ±10%. The other quantities are calculated based on these perturbed parameters.

$$
\begin{aligned}
\hat{\bar{c}}_3 &\in \bar{c}_3 \pm 10\% \\
\hat{\bar{c}}_1 &\in \bar{c}_1 \pm 10\% \\
\hat{a}_1^{max} &\in a_1^{max} \pm 10\% \\
\alpha &\in [0.9, 1.1] \\
\hat{\bar{p}}_1 &= \bar{p}_1 \cdot \alpha \\
\hat{\bar{p}}_2 &= [\bar{p}_2^1, ..., \bar{p}_2^N]^T \cdot \alpha \\
\hat{k}_2 &= (2\hat{a}_1^{max} - 1)/\hat{\bar{c}}_3 \\
\hat{d}_3 &\in d_3 \pm 10\% \\
\hat{a}_2^{max,i} & \text{calculated as in Section 2.1 based on } \hat{d}_3,\ \hat{\bar{c}}_3,\ \hat{\bar{p}}_1,\ \hat{\bar{c}}_1,\ \hat{k}_2 \\
\beta &\in [0.9, 1.1] \cdot 4 \\
\hat{p}_2^{i,max} &= \beta \cdot \hat{\bar{p}}_2^i && (1 \leq i \leq N) \\
\gamma &\in [0.9, 1.1] \cdot 8 \\
\hat{p}_1^{max} &= \gamma \cdot \hat{\bar{p}}_1 \\
\hat{k}_1 &= (\beta - 1)/\hat{\bar{c}}_3 \\
\hat{k}_0 &= (\gamma - 1)/\hat{\bar{c}}_3
\end{aligned}
$$

Kinetics of growth factor support are not perturbed and parameterized as in Section 2.3. If applied, growth factor support starts at day 3 after transplantation. We set $\hat{S} = 1.3$.

2.4.2 Mutated cell parameters for saturated expansion

$$
\begin{aligned}
\hat{\sigma}_{a_1} &\in [1, 1.8] \\
\hat{\sigma}_{a_2} &\in [1, 1.8] \\
\hat{\sigma}_{p_1} &\in [1, 1.8] \\
\hat{\sigma}_{p_2} &\in [1, 1.8] \\
\delta &\in [1, 1.5] \\
\hat{p}_1^{max,M} &= \delta \cdot \hat{p}_1^{max} \\
\hat{p}_2^{max,M} &= \delta \cdot \hat{p}_2^{max} \\
\hat{d}_3^M &\in [0.9, 1.1] \cdot \hat{d}_3
\end{aligned}
$$

The range $[1, 1.8]$ was chosen to observe approximately a doubling of donor VAFs for the smallest transplant dose. This is close to the lower boundary of the fold-expansions observed in literature. We made this choice not to overestimate the effects. In some patients the effects might be larger.

Kinetics of growth factor support are not perturbed and parameterized as in Section 2.3. If applied,

growth factor support starts at day 3 after transplantation. We set $\hat{S} = 1.3$. The donor VAF was randomly chosen between 0.005 and 0.125.

2.4.3 Mutated cell parameters for persistent expansion

$$
\begin{aligned}
\hat{\sigma}_{a_1} &\in [1, 1.8] \\
\hat{\sigma}_{a_2} &\in [1, 1.8] \\
\hat{\sigma}_{p_1} &\in [1, 1.8] \\
\hat{\sigma}_{p_2} &\in [1, 1.8] \\
\delta &\in [1, 1.5] \\
\hat{p}_1^{max,M} &= \delta \cdot \hat{p}_1^{max} \\
\hat{p}_2^{max,M} &= \delta \cdot \hat{p}_2^{max} \\
\hat{a}_1^{max,M} &= [1, 1.2] \cdot \hat{a}_1^{max} \\
\hat{a}_2^{max,M} &= [1, 1.2] \cdot \hat{a}_2^{max} \\
\hat{d}_3^{M} &\in [0.9, 1.1] \cdot \hat{d}_3
\end{aligned}
$$

Kinetics of growth factor support are not perturbed and parameterized as in Section 2.3. If applied, growth factor support starts at day 3 after transplantation. We set $\hat{S} = 1.3$. The donor VAF was randomly chosen between 0.005 and 0.125.

3 Parameter sets used in the figures

Figure 1 (B), SFig 4 and SFig 5

Cell parameters as in Section 2.1. Initial condition for transplantation as in Section 2.2 with $h_{HSC} = h_{HPC} = 0.5$. Start of growth factor support at day 3 after transplantation. Parameters of cytokine support are as specified in Section 2.3 with $\hat{S} = 1.3$. The transplanted cell dose is set to $4.7 \cdot 10^6$ $CD34^+$ cells per kg of body weight, as reported in [40]. This corresponds to the initial condition for transplantation from Section 2.2 multiplied by 0.94.

Figure 1 (C), (D)

Parameters as specified in Section 2.4.1. In addition we set $\hat{h}_{HSC} \in [0.175; 0.825]$ and $\hat{h}_{HPC} = \hat{h}_{HSC}$ The transplanted cell dose is set to $4.7 \cdot 10^6$ $CD34^+$ cells per kg of body weight, as reported in [40]. This corresponds to the initial condition for transplantation from Section 2.2 multiplied by 0.94. Parameters of cytokine support are as specified in Section 2.3 with $\hat{S} = 1.3$.

Figure 3 (B), (C); Figure 6 (C)

Wildtype cell parameters as in Section 2.1. Maximal proliferation rates, clearance rate and maximal self-renewal probabilities of wildtype and mutated cells are identical, i.e., $p_1^{max} = p_1^{max,M}$, $a_1^{max} = a_1^{max,M}$,

$p_2^{i,max} = p_2^{i,max,M}$, $a_2^{i,max} = a_2^{i,max,M}$, $d_3 = d_3^M$. The transplanted cell dose was set to $3.6 \cdot 10^6$ CD34$^+$ cells per kg of body weight, corresponding to $7 \cdot 10^5$ cells per kg of body weight contributing to granulopoiesis and the following graft:
[4000; 34; 53; 105; 232; 549; 1348; 3404; 8773; 22959; 60824; 162737; 438983; 0; 0; 0; 0].
Furthermore, $h_{HSC} = h_{HPC} = 1$. The VAF in the transplant is 0.1 for each cell type (20% of cells are mutated). The feedback response of mutated cells is as follows:

- blue line: $\sigma_{a_1} = \sigma_{p_1} = \sigma_{a_2} = \sigma_{p_2} = 4$. These parameters are also used in Figure 6 (C).
- red line: $\sigma_{a_1} = \sigma_{p_1} = 4$, $\sigma_{a_2} = \sigma_{p_2} = 1$
- yellow line: $\sigma_{a_1} = \sigma_{p_1} = 1$, $\sigma_{a_2} = \sigma_{p_2} = 4$
- purple line: $\sigma_{a_1} = \sigma_{p_1} = \sigma_{a_2} = 1$, $\sigma_{p_2} = 4$

Figure 3 (E), (F)

Wildtype cell parameters as in Section 2.1. Maximal proliferation rates, clearance rate and maximal self-renewal probabilities of wildtype and mutated cells are identical, i.e., $p_1^{max} = p_1^{max,M}$, $a_1^{max} = a_1^{max,M}$, $p_2^{i,max} = p_2^{i,max,M}$, $a_2^{i,max} = a_2^{i,max,M}$, $d_3 = d_3^M$. The transplant composition is as in Figure 3 (B), (C). Furthermore, $\sigma_{a_1} = \sigma_{p_1} = \sigma_{a_2} = \sigma_{p_2} = 1$. The VAF in the transplant is 0.25 for each cell type (50% of cells are mutated). The homing capacities are chosen as follows:

- blue line: $h_{HSC} = h_{HPC} = 0.5$ for wildtype, $h_{HSC} = h_{HPC} = 0.5$ for mutated
- red line: $h_{HSC} = h_{HPC} = 0.5$ for wildtype, $h_{HSC} = h_{HPC} = 0.8$ for mutated
- yellow line: $h_{HSC} = h_{HPC} = 0.8$ for wildtype, $h_{HSC} = h_{HPC} = 0.5$ for mutated
- green line: $h_{HSC} = h_{HPC} = 0.5$ for wildtype, $h_{HSC} = 0.7,\ h_{HPC} = 0.9$ for mutated
- purple line: $h_{HSC} = h_{HPC} = 0.5$ for wildtype, $h_{HSC} = 0.9,\ h_{HPC} = 0.7$ for mutated

Figure 4 (B), (C); Figure 6 (A)

Wildtype cell parameters as in Section 2.1. The transplanted cell dose is set to $3.6 \cdot 10^6$ CD34$^+$ cells per kg of body weight, corresponding to $\approx 7 \cdot 10^5$ cells per kg of body weight contributing to granulopoiesis (Section 2.2) and ≈ 4000 HSCs per kg of body weight, as for Fig. 3 (B), (C). At the time of donation the donor PB VAF equals 0.1. The graft composition is assumed to reflect the marrow composition of the donor: The ratio of mutated to wildtype HSCs in the graft is assumed to equal the ratio of mutated to wildtype HSCs in the donor's marrow. Analogously, the ratio of mutated to wildtype HPCs in the graft is assumed to equal the ratio of mutated to wildtype HPCs in the donor's marrow. In situations where mutated HSCs have a growth advantage under homeostatic conditions the clonal dynamics in the donor are simulated starting from wildtype cell homeostasis and one mutated HSC and run until the PB VAF is 0.1. In all other cases the mutated HSC number in the donor is chosen such that under homeostatic conditions the donor VAF equals 0.1. The progenitor cell doses in the grafts are obtained by $c_2^{i,graft} = \frac{c_2^i(t_d)}{\sum_{i=1}^{12} c_2^i(t_d) + \sum_{i=1}^{12} c_2^{i,M}(t_d)} \cdot D$ $(1 \leq i \leq 12)$, where t_d is the time of donation, $c_2^i(t_d)$ the abundance of the

ith wildtype progenitor compartment in the donor's marrow and D the total dose of transplanted cells. Analogously, we set $c_2^{i,M,graft} = \frac{c_2^{i,M}(t_d)}{\sum_{i=1}^{12} c_2^i(t_d)+\sum_{i=1}^{12} c_2^{i,M}(t_d)} \cdot D$ $(1 \leq i \leq 12)$. We set $h_{HSC} = h_{HPC} = 1$ and $\sigma_{a_1} = \sigma_{p_1} = \sigma_{a_2} = \sigma_{p_2} = 1$. Mutated cell parameters are as follows:

- blue line: $1.15 \cdot p_1^{max} = p_1^{max,M}$, $1.15 \cdot a_1^{max} = a_1^{max,M}$, $1.15 \cdot p_2^{i,max} = p_2^{i,max,M}$, $1.15 \cdot a_2^{i,max} = a_2^{i,max,M}$, $d_3 = d_3^M$. These parameters are also used in Figure 6 (A).
- red line: $1.15 \cdot p_1^{max} = p_1^{max,M}$, $1.15 \cdot a_1^{max} = a_1^{max,M}$, $p_2^{i,max} = p_2^{i,max,M}$, $a_2^{i,max} = a_2^{i,max,M}$, $d_3 = d_3^M$
- yellow line: $p_1^{max} = p_1^{max,M}$, $a_1^{max} = a_1^{max,M}$, $1.15 \cdot p_2^{i,max} = p_2^{i,max,M}$, $1.15 \cdot a_2^{i,max} = a_2^{i,max,M}$, $d_3 = d_3^M$
- purple line: $p_1^{max} = p_1^{max,M}$, $a_1^{max} = a_1^{max,M}$, $1.15 \cdot p_2^{i,max} = p_2^{i,max,M}$, $a_2^{i,max} = a_2^{i,max,M}$, $d_3 = d_3^M$

Figure 4 (E), (F); Figure 6 (B)

We consider the model from Section 1.5. Wildtype cell parameters as in Section 2.1. Maximal proliferation rates, clearance rate and maximal self-renewal probabilities of wildtype and mutated cells are identical, i.e., $p_1^{max} = p_1^{max,M}$, $a_1^{max} = a_1^{max,M}$, $p_2^{i,max} = p_2^{i,max,M}$, $a_2^{i,max} = a_2^{i,max,M}$, $d_3 = d_3^M$. The transplant composition is as in Figure 4 (B), (C), $h_{HSC} = h_{HPC} = 1$ and $\sigma_{a_1} = \sigma_{p_1} = \sigma_{a_2} = \sigma_{p_2} = 1$. We set $\iota = 0$; $k_{infl} = 10^{-3}$, $\alpha_{infl} = 10^{-4}$, $\tilde{\delta} = 0.1$. The donor PB VAF at the time of donation equals 0.1. In situations where mutated HSCs have a growth advantage under homeostatic conditions the clonal dynamics in the donor are simulated starting from wildtype cell homeostasis and one mutated HSC and run until the PB VAF is 0.1. In all other cases the mutated HSC number in the donor is chosen such that under homeostatic conditions the donor VAF equals 0.1.

- blue line: feedback on mutated HSC self-renewal as in equation (28), feedback on mutated HPC self-renewal as in equation (29), feedback on mutated HSC proliferation as in equation (30), feedback on mutated HPC proliferation as in equation (31). These parameters are also used in Figure 6 (B).
- red line: feedback on mutated HSC self-renewal as in equation (28), feedback on mutated HSC proliferation as in equation (30), all other feedbacks as in system (17)
- yellow line: feedback on mutated HPC self-renewal as in equation (29), feedback on mutated HPC proliferation as in equation (31), all other feedbacks as in system (17)
- purple line: feedback on mutated HPC proliferation as in equation (31), all other feedbacks as in system (17)

Figure 4 (H), (I), Supplemental Figure 6

We consider the model from Section 1.6. Wildtype cell parameters are as in Section 2.1. Maximal proliferation rates, clearance rate and maximal self-renewal probabilities of wildtype and mutated cells are identical, i.e., $p_1^{max} = p_1^{max,M}$, $a_1^{max} = a_1^{max,M}$, $p_2^{i,max} = p_2^{i,max,M}$, $a_2^{i,max} = a_2^{i,max,M}$, $d_3 = d_3^M$. The transplant composition is as in Figure 4 (B), (C), $h_{HSC} = h_{HPC} = 1$ and $\sigma_{a_1} = \sigma_{p_1} = \sigma_{a_2} = \sigma_{p_2} = 1$. We set $\iota = 0$; $\tilde{k}_{infl} = 10^{-3}$, $\zeta = 0.1$, $\tilde{\zeta} = 2$, $\tilde{\delta} = 0.1$. The donor PB VAF at the time of donation equals 0.1. In situations where mutated HSCs have a growth advantage under homeostatic conditions, the clonal dynamics in the

donor are simulated starting from wildtype cell homeostasis and one mutated HSC per kg of body weight and run until the PB VAF is 0.1. In all other cases the mutated HSC number in the donor is chosen such that under homeostatic conditions the donor VAF equals 0.1.

Figure 4 (G), (H)

- blue line: feedback on wildtype HSC self-renewal as in equation (32), feedback on wildtype HPC self-renewal as in equation (33), all other feedbacks as in system (17).
- red line: feedback on wildtype HSC self-renewal as in equation (32), all other feedbacks as in system (17).
- yellow line: feedback on wildtype on wildtype HPC self-renewal as in equation (33), all other feedbacks as in system (17).

Supplemental Figure 6

- blue line: feedback on wildtype HSC self-renewal as in equation (32), feedback on wildtype HPC self-renewal as in equation (33), feedback on wildtype HSC proliferation as in equation (34), feedback on wildtype HPC proliferation as in equation (35).
- red line: feedback on wildtype HSC self-renewal as in equation (32), feedback on wildtype HSC proliferation as in equation (34), all other feedbacks as in system (17).
- yellow line: feedback on wildtype on wildtype HPC self-renewal as in equation (33), feedback on wildtype HPC proliferation as in equation (35), all other feedbacks as in system (17).

Figure 5 (A)

Wildtype cell parameters as in Section 2.1. Maximal proliferation rates, clearance rate and maximal self-renewal probabilities of wildtype and mutated cells are identical, i.e., $p_1^{max} = p_1^{max,M}$, $a_1^{max} = a_1^{max,M}$, $p_2^{i,max} = p_2^{i,max,M}$, $a_2^{i,max} = a_2^{i,max,M}$, $d_3 = d_3^M$. $\sigma_{a_1} = \sigma_{a_2} = 1.91$, $\sigma_{p_1} = 1.8$, $\sigma_{p_2} = 1.26$. The variant allele frequency of each cell type in the graft is 0.063, the graft is as in Figure 3 (C), $h_{HSC} = h_{HPC} = 0.5$.

Figure 5 (B)

Wildtype cell parameters as in Section 2.1. Maximal proliferation rates, clearance rate and maximal self-renewal probabilities of mutated cells are $1.4 \cdot p_1^{max} = p_1^{max,M}$, $a_1^{max} = a_1^{max,M}$, $2.3 \cdot p_2^{i,max} = p_2^{i,max,M}$, $1.11 \cdot a_2^{i,max} = a_2^{i,max,M}$, $d_3 = d_3^M$. $\sigma_{a_1} = \sigma_{a_2} = \sigma_{p_1} = 2.1$, $\sigma_{p_2} = 1$. The donor's PB VAF equals 0.088 at the time of donation. The transplanted cell dose is set to $3.6 \cdot 10^6$ $CD34^+$ cells per kg of body weight, corresponding to $7 \cdot 10^5$ cells per kg of body weight contributing to granulopoiesis, as in Fig. 3 (B), (C). We set $h_{HSC} = h_{HPC} = 0.31$ for wildtype cells and $h_{HSC} = h_{HPC} = 0.85$ for mutated cells. The transplant composition reflects the steady state ratios between the compartments. This leads to the following graft. Wildtype cells:
[3805.78; 30.45; 46.53; 92.44; 205.26; 484.85; 1191.45; 3009.15; 7754.64; 20294.61; 53764.76; 143849.73; 388035.52;

0; 0; 0; 0]
Mutated cells:
[194.22; 1.06; 1.74; 3.71; 8.86; 22.54; 59.6; 174.94; 553.69; 1752.43; 5546.48; 17554.69; 55560.88; 0; 0; 0; 0]

Figure 5 (C)

Wildtype cell parameters as in Section 2.1. Maximal proliferation rates, clearance rate and maximal self-renewal probabilities of wildtype and mutated cells are identical, i.e., $p_1^{max} = p_1^{max,M}$, $a_1^{max} = a_1^{max,M}$, $p_2^{i,max} = p_2^{i,max,M}$, $a_2^{i,max} = a_2^{i,max,M}$, $d_3 = d_3^M$. $\sigma_{a_1} = \sigma_{a_2} = \sigma_{p_1} = \sigma_{p_2} = 1$. The variant allele frequency of each cell type in the graft is 0.088, the graft cell counts are as in Figure 3 (C), $h_{HSC} = h_{HPC} = 0.08$ for wildtype cells and $h_{HSC} = h_{HPC} = 0.95$ for mutated cells.

Figure 5 (D)

Wildtype cell parameters as in Section 2.1. Maximal proliferation rates, clearance rate and maximal self-renewal probabilities of mutated cells are $p_1^{max} = p_1^{max,M}$, $a_1^{max} = a_1^{max,M}$, $1.1 \cdot p_2^{i,max} = p_2^{i,max,M}$, $a_2^{i,max} = a_2^{i,max,M}$, $d_3 = d_3^M$, $\sigma_{a_1} = 1$, $\sigma_{a_2} = 1.1$, $\sigma_{p_1} = 1$, $\sigma_{p_2} = 1.7$. The donor's PB VAF equals 0.001 at the time of donation. The transplanted cell dose is set to $3.6 \cdot 10^6$ $CD34^+$ cells per kg of body weight, corresponding to $7 \cdot 10^5$ cells per kg of body weight contributing to granulopoiesis, as in Fig. 3 (B), (C). We set $h_{HSC} = h_{HPC} = 0.55$. The transplant composition reflects the steady state ratios between the compartments. This leads to the following graft. Wildtype cells:
[3920, 33.83, 51.69, 102.67, 227.98, 538.52, 1323.33, 3342.23, 8613, 22541.01, 59715.96, 159772.38, 430986.97, 0, 0, 0, 0]
Mutated cells:
[80, 0.63, 0.96, 1.9, 4.23, 9.99, 24.55, 62.01, 159.8, 418.2, 1107.9, 2964.24, 7996.05, 0, 0, 0, 0]

Figure 5 (E)

Wildtype cell parameters as in Section 2.1. Maximal proliferation rates, clearance rate and maximal self-renewal probabilities of mutated cells are $p_1^{max} = p_1^{max,M}$, $1.4 \cdot a_1^{max} = a_1^{max,M}$, $p_2^{i,max} = p_2^{i,max,M}$, $a_2^{i,max} = a_2^{i,max,M}$, $d_3 = d_3^M$, $\sigma_{a_1} = \sigma_{a_2} = 1.38$, $\sigma_{p_1} = 1.07$, $\sigma_{p_2} = 1$. The donor's PB VAF equals 0.06 at the time of donation. The transplanted cell dose is set to $3.6 \cdot 10^6$ $CD34^+$ cells per kg of body weight, corresponding to $7 \cdot 10^5$ cells per kg of body weight contributing to granulopoiesis and calculated as in Fig. 4 (B), (C). We set $h_{HSC} = h_{HPC} = 0.3$.

The graft composition is assumed to reflect the marrow composition of the donor: The ratio of mutated to wildtype HSCs in the graft is assumed to equal the ratio of mutated to wildtype HSCs in the donor's marrow. Analogously, the ratio of mutated to wildtype HPCs in the graft is assumed to equal the ratio of mutated to wildtype HPCs in the donor's marrow. The clonal dynamics in the donor are simulated starting from wildtype cell homeostasis and one mutated HSC and run until the PB VAF at donation time is reached.

Figure 5 (F)

Wildtype cell parameters as in Section 2.1. Maximal proliferation rates, clearance rate and maximal self-renewal probabilities of mutated cells are $p_1^{max} = p_1^{max,M}$, $1.28 \cdot a_1^{max} = a_1^{max,M}$, $1.3 \cdot p_2^{i,max} = p_2^{i,max,M}$,

$a_2^{i,max} = a_2^{i,max,M}$, $d_3 = d_3^M$. $\sigma_{a_1} = \sigma_{a_2} = 1.27$, $\sigma_{p_1} = 1$, $\sigma_{p_2} = 1.05$. The donor's PB VAF equals 0.04 at the time of donation. The transplanted cell dose is set to $3.6 \cdot 10^6$ $CD34^+$ cells per kg of body weight, corresponding to $7 \cdot 10^5$ cells per kg of body weight contributing to granulopoiesis, $h_{HSC} = h_{HPC} = 0.5$.

The graft composition is assumed to reflect the marrow composition of the donor: The ratio of mutated to wildtype HSCs in the graft is assumed to equal the ratio of mutated to wildtype HSCs in the donor's marrow. Analogously, the ratio of mutated to wildtype HPCs in the graft is assumed to equal the ratio of mutated to wildtype HPCs in the donor's marrow. The clonal dynamics in the donor are simulated starting from wildtype cell homeostasis and one mutated HSC and run until the PB VAF at doation time is reached.

Figure 6 (D)

Wildtype cell parameters as in Section 2.1. Maximal proliferation rates, clearance rate and maximal self-renewal probabilities of wildtype and mutated cells are identical, i.e., $p_1^{max} = p_1^{max,M}$, $a_1^{max} = a_1^{max,M}$, $p_2^{i,max} = p_2^{i,max,M}$, $a_2^{i,max} = a_2^{i,max,M}$, $d_3 = d_3^M$. The transplanted cell dose is set to $3.6 \cdot 10^6$ $CD34^+$ cells per kg of body weight, corresponding to $7 \cdot 10^5$ cells per kg of body weight contributing to granulopoiesis and a graft as in Figure 3 (B). Furthermore, $h_{HSC} = h_{HPC} = 1$. The VAF in the transplant is 0.1 for each cell type (20% of cells are mutated). Furthermore, $\sigma_{a_1} = \sigma_{p_1} = 1.1$, $\sigma_{a_2} = \sigma_{p_2} = 1$.

Figure 7 (A)-(D)

Wildtype cell parameters as in Section 2.4.1, mutated cell parameters as in Section 2.4.2. The homing parameter is fixed at $h_{HSC} = h_{HPC} = 0.5$ for all individuals, instead 4 different transplant doses are considered. These doses are within clinically plausible ranges [27].

- dose 1: $1 \cdot 10^6$ CD34+ cells per kg of body weight, corresponding to $1.9 \cdot 10^5$ progenitors (CFU-G and CFU-GM) and 800 HSCs per kg of body weight, see Section 2.2.
- dose 2: $5 \cdot 10^6$ CD34+ cells per kg of body weight, corresponding to $9.7 \cdot 10^5$ progenitors (CFU-G and CFU-GM) and 4000 HSCs per kg of body weight, see Section 2.2.
- dose 3: $1 \cdot 10^7$ CD34+ cells per kg of body weight, corresponding to $1.9 \cdot 10^6$ progenitors (CFU-G and CFU-GM) and 8000 HSCs per kg of body weight, see Section 2.2.
- dose 4: $1.5 \cdot 10^7$ CD34+ cells per kg of body weight, corresponding to $2.9 \cdot 10^6$ progenitors (CFU-G and CFU-GM) and 12000 HSCs per kg of body weight, see Section 2.2.

Since $a_1^{max} = a_1^{max,M}$ mutated HSCs do not expand in absence of hematopoietic stress or infection. Donor marrow mutated HSC counts are chosen such that the equilibrium (equilibrium with nonzero wildtype and nonzero mutated cells) exhibits the randomly chosen donor PB VAF. Ratios of the different CD 34+ cell types in the graft equal the respective ratios in the donor's marrow. The ratio of mutated to wildtype cells in each CD 34+ compartment in the graft equals the respective ratio in the donor's marrow. Panels (A)-(B) are without cytokine support, panels (C)-(D) with cytokine support. Parameters of cytokine support are as specified in Section 2.3 with $\hat{S} = 1.3$.

Figure 7 (E)-(H)

Wildtype cell parameters as in Section 2.4.1, mutated cell parameters as in Section 2.4.3. The homing parameter is fixed at $h_{HSC} = h_{HPC} = 0.5$ for all individuals, instead 4 different transplant doses are considered. These doses are the same as in Figure 7 (A)-(D).

The pre-donation period of the donor is simulated starting from the healthy equilibrium and one mutated HSC per kg of body weight. Simulations are run until the donor PB VAF in the simulations equals the prescribed randomly chosen donor PB VAF at the time of transplantation. If the prescribed donor PB VAF is not reached within 50 years the parameter set is excluded. Ratios of the different CD 34+ cell types in the graft equal the respective ratios in the donor's marrow. The ratio of mutated to wildtype cells in each CD 34+ compartment in the graft equals the respective ratio in the donor's marrow. Panels (E)-(F) are without cytokine support, panels (G)-(H) with cytokine support. Parameters of cytokine support are as specified in Section 2.3 with $\hat{S} = 1.3$.

Figure 8 (A)

Parameters as in Figure 7 (A)-(B). Transplanted HSC doses as in Figure 7 (A)-(B). Transplanted CD34 $^+$ progenitor dose fixed at $9.7 \cdot 10^5$ per kg of body weight.

Figure 8 (B)

Parameters as in Figure 7 (A)-(B). Transplanted CD34 $^+$ HPC doses as in as in Figure 7 (A)-(B). Transplanted HSC dose fixed at 4000 per kg of body weight.

Figure 8 (C)

Parameters as in Figure 7 (E)-(F). Transplanted HSC doses as in Figure 7 (E)-(F). Transplanted CD34 $^+$ progenitor dose fixed at $9.7 \cdot 10^5$ per kg of body weight.

Figure 8 (D)

Parameters as in Figure 7 (E)-(F). Transplanted CD34 $^+$ HPC doses as in as in Figure 7 (E)-(F). Transplanted HSC dose fixed at 4000 per kg of body weight.

Figure 9 (A)-(C)

Parameters as in Figure 7 (A)-(B), no cytokine support, transplanted cells equal to dose 1.

Figure 9 (D)-(I)

Parameters as in Figure 7 (E)-(F), no cytokine support, transplanted cells equal to dose 1.

Figure 10 (A)-(C)

We consider the model from Section 1.5. Wildtype cell parameters as in Section 2.4.1. Mutated cell parameters equal to wildtype cell parameters, except $a_1^{max,M} = 0.6$, $\sigma_{a_1} = \sigma_{a_2} = \sigma_{p_1} = \sigma_{p_2} = 1$, $k_{infl} = 10^{-8}$, $\tilde{\delta} = 0$, $\alpha_{infl} = 5 \cdot 10^{-9}$. Background inflammation in donor and recipient 1 is $\iota = 7.4 \cdot 10^7$, in recipient 3 $\iota = 7.4 \cdot 10^6$, in recipient 2 $\iota = 2.9 \cdot 10^7$. The transplanted cell dose is set to $3.6 \cdot 10^6$ CD34$^+$ cells per kg of body weight, corresponding to $7 \cdot 10^5$ cells per kg of body weight contributing to granulopoiesis and 4000 HSCs per kg of body weight. Donor's bone marrow composition is obtained by simulating clonal dynamics in the donor starting from healthy equilibrium and one mutated HSC per kg of body weight.

Figure 10 (D)-(I)

We consider the model from Section 1.5. Wildtype cell parameters as in Section 2.4.1. Mutated cell parameters equal to wildtype cell parameters, except $a_1^{max,M} = 0.6$, $\sigma_{a_1} = \sigma_{a_2} = \sigma_{p_1} = \sigma_{p_2} = 1$, $k_{infl} = 10^{-8}$, $\tilde{\delta} = 0.1$, $\alpha_{infl} = 5 \cdot 10^{-9}$. Background inflammation in the donor is $\iota = 7.4 \cdot 10^7$, in the recipients it is $\iota = 0$. The transplanted cell dose is set to $3.6 \cdot 10^6$ CD34$^+$ cells per kg of body weight, corresponding to $7 \cdot 10^5$ cells per kg of body weight contributing to granulopoiesis and 4000 HSCs per kg of body weight. Donor's bone marrow composition is obtained by simulating clonal dynamics in the donor starting from healthy equilibrium and one mutated HSC per kg of body weight.

Supplemental Figure 7

Wildtype cell parameters as in Section 2.1. Clone 1: $p_1^{max} = p_1^{max,M}$, $a_1^{max,M} = 0.7021$, $p_2^{i,max} = p_2^{i,max,M}$, $a_2^{i,max} = a_2^{i,max,M}$, $d_3 = d_3^M$, $\sigma_{a_1} = 1$, $\sigma_{a_2} = 1.55$, $\sigma_{p_1} = 0.73$ (this means that mutated HSC proliferation rate is less responsive to feedback signals compared to wildtype HSC proliferation rate. This is in line with the concept that mutated cells are resistant to inflammatory stimuli [3]), $\sigma_{p_2} = 1$. Clone 2: $p_1^{max,M} = 0.0065/day$, $a_1^{max,M} = 0.7930$, $p_2^{i,max} = p_2^{i,max,M}$, $a_2^{i,max} = a_2^{i,max,M}$, $d_3 = d_3^M$, $\sigma_{a_1} = 1$, $\sigma_{a_2} = 1$, $\sigma_{p_1} = 1$,$\sigma_{p_2} = 1$. Clone 1 is introduced 42.4 years before transplantation, i.e., 1 mutated HSC per kg of body weight is added to the healthy equilibrium, clone 2 is introduced 10.1 years before transplantation, i.e., 1 HSC per kg of body weight is added to the monoclonal system.

The donor's PB VAFs for clones 1 and 2 equal 0.026 and 0.006 at the time of donation, respectively. The transplanted cell dose is set to $3.6 \cdot 10^6$ CD34$^+$ cells per kg of body weight, corresponding to $7 \cdot 10^5$ cells per kg of body weight contributing to granulopoiesis and calculated as in Fig. 4 (B), (C). We set $h_{HSC} = h_{HPC} = 0.7$.

Supplemental Figure 8-10

We use the model from Supplemental Section 1.4.1. The parameters of Supplemental Figures 8 - 10 are as in Figures 1, 7 and 8, respectively. In addition, we set $\tilde{k}_2 = (2a_1^{max} - 1)/\bar{c}_1$ for Supplemental Figure 8 (B) and in the virtual trials we set $\tilde{k}_2 = (2\hat{a}_1^{max} - 1)/\hat{\bar{c}}_1$, where $\hat{a}_1^{max}$ and $\hat{\bar{c}}_1$ are the individual parameters of the respective virtual patient. These modifications imply that the HSC self-renewal probability is regulated by a local (i.e., HSC-dependent) signal instead of a systemic (i.e., mature cell-dependent) signal. The ratio of equilibrium HSC self-renewal and HSC self-renewal under maximal endogenous stimulation (i.e., $\tilde{s}_2 = 1$) is as in Figures 1, 7 and 8.

Supplemental Figure 11-13

We use the model from Supplemental Section 1.4.2. HSC self-renewal is parametrized as in Supplemental Figures 8 - 10. In addition, we set $\tilde{k}_0 = 7/\bar{c}_1$ for Supplemental Figure 11 (B) and in the virtual trials we set $\tilde{k}_0 = (\gamma - 1)/\hat{\bar{c}}_1$, where γ and $\hat{\bar{c}}_1$ are the individual parameters of the respective virtual patient.

The other parameters of Supplemental Figure 11 - 13 are as in Figures 1, 7 and 8, respectively. These modifications imply that the HSC self-renewal probability and proliferation rate are regulated by local (i.e., HSC-dependent) signals instead of systemic (i.e., mature cell-dependent) signals. The ratio of equilibrium HSC self-renewal and HSC self-renewal under maximal endogenous stimulation (i.e., $\tilde{s}_2 = 1$) is as in Figures 1, 7 and 8. Furthermore, the ratio of equilibrium HSC proliferation and HSC proliferation under maximal endogenous stimulation (i.e., $\tilde{s}_0 = 1$) is as in Figures 1, 7 and 8.

4 Additional Figures

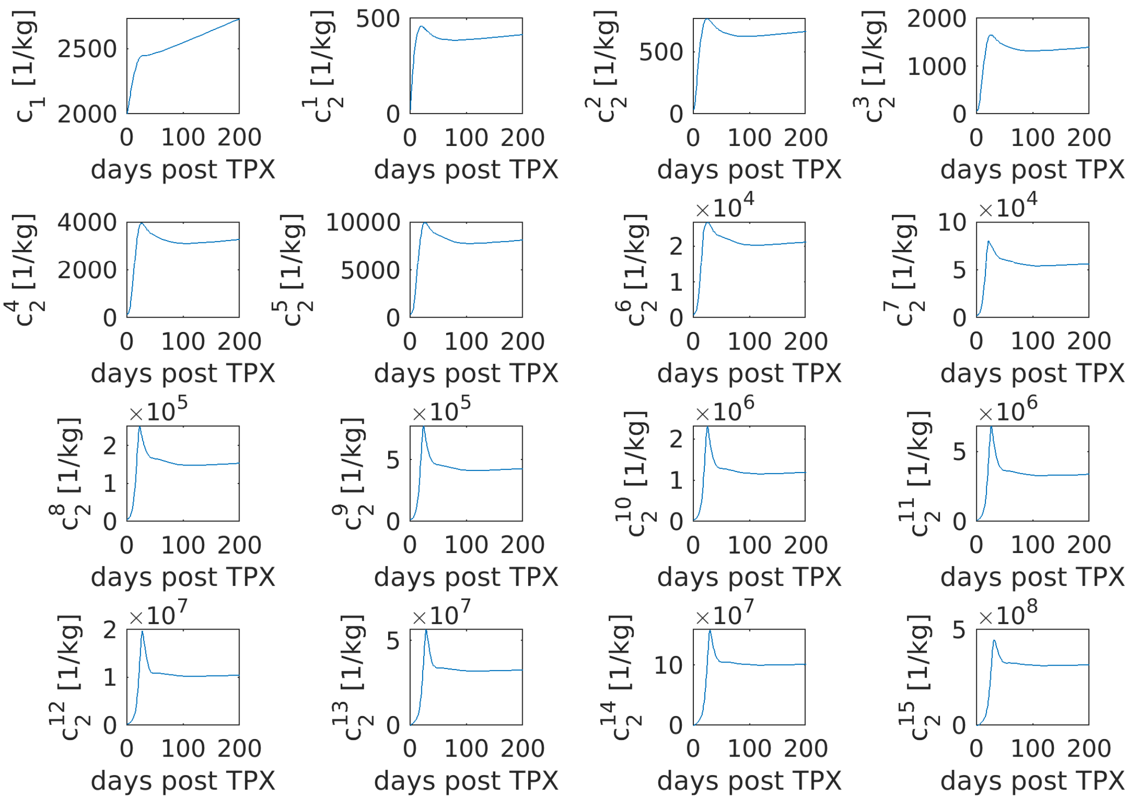

Supplementary Figure 4: **Simulated time dynamics of immature cells after allogeneic transplantation without growth factor support.** Mature cell dynamics are shown in Fig. 1 (B) of the main text.

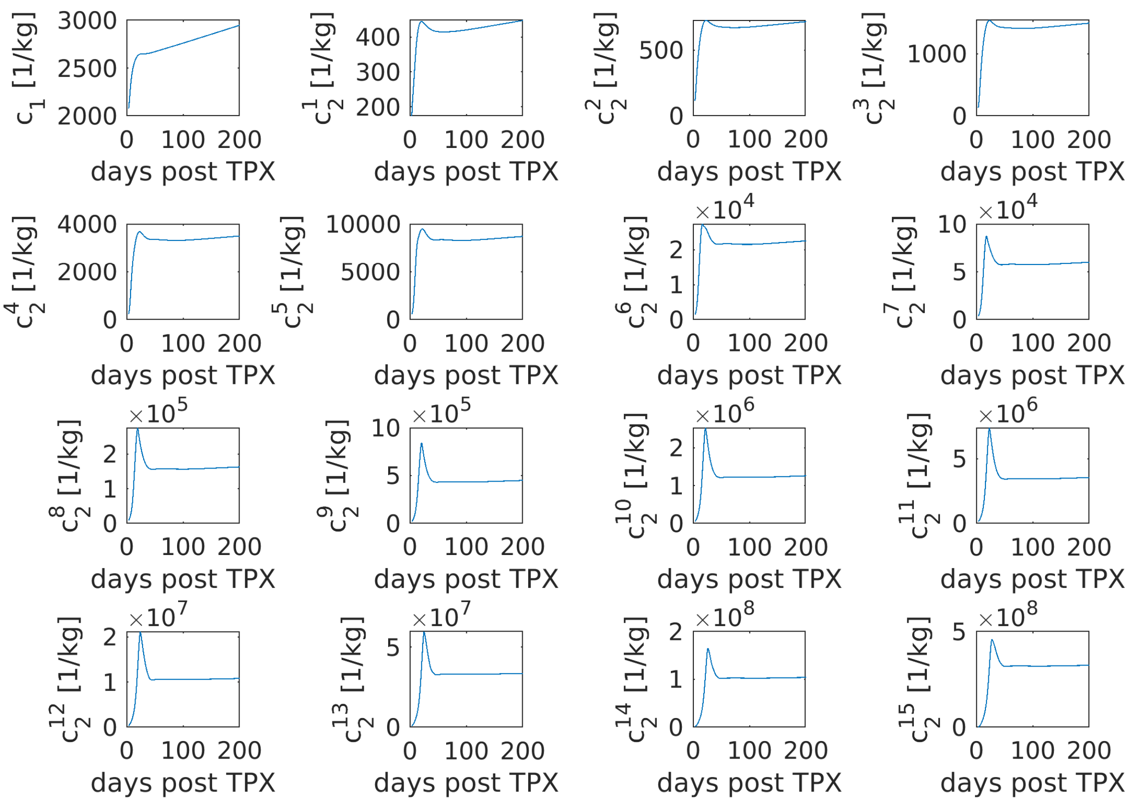

Supplementary Figure 5: **Simulated time dynamics of immature cells after allogeneic transplantation with growth factor support.** Mature cell dynamics are shown in Fig. 1 (B) of the main text.

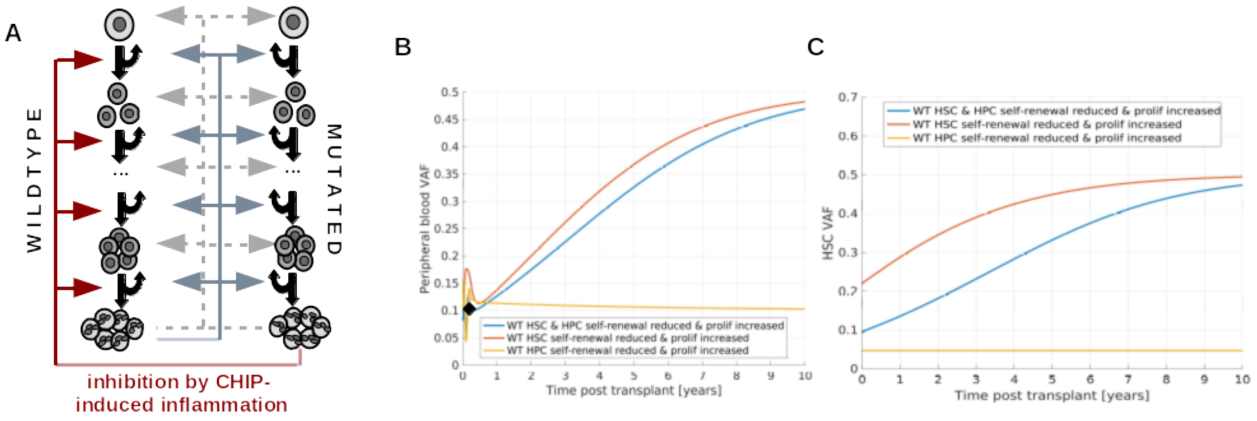

Supplementary Figure 6: **Impairment of wildtype cells by chronic inflammation.** The figure considers a scenario where CHIP-induced chronic inflammation reduces the fitness of wildtype cells. (A) Mutated white blood cells trigger chronic inflammation. The higher the inflammatory burden, the lower the self-renewal probability of wildtype HSC and the amplification of wildtype progenitors and the higher the proliferation rate of wildtype cells. Dynamics of PB VAF after HSCT. (B) The black diamond indicates the VAF in the donor's PB. Blue line: Wildtype HSC self-renewal probability and HPC amplification are reduced in response to the inflammatory cytokines. Wildtype HSC and HPC proliferation rates are increased in response to the inflammatory cytokines. Red line: Wildtype HSC self-renewal probability is decreased in response to the inflammatory cytokines. Wildtype HSC proliferation rate is increased in response to the inflammatory cytokines. Yellow line: Wildtype HPC amplification are reduced in response to the inflammatory cytokines and wildtype HPC proliferation rate is increased. Since HSC properties are identical for mutated and wildtype cells we observe no persistent VAF increase. (C) VAF dynamics in the stem cell compartment. Color coding as in (B). Compared to Figure 4 (G)-(I) of the main text, where proliferation rates are not affected by chronic inflammation, we observe faster VAF dynamics.

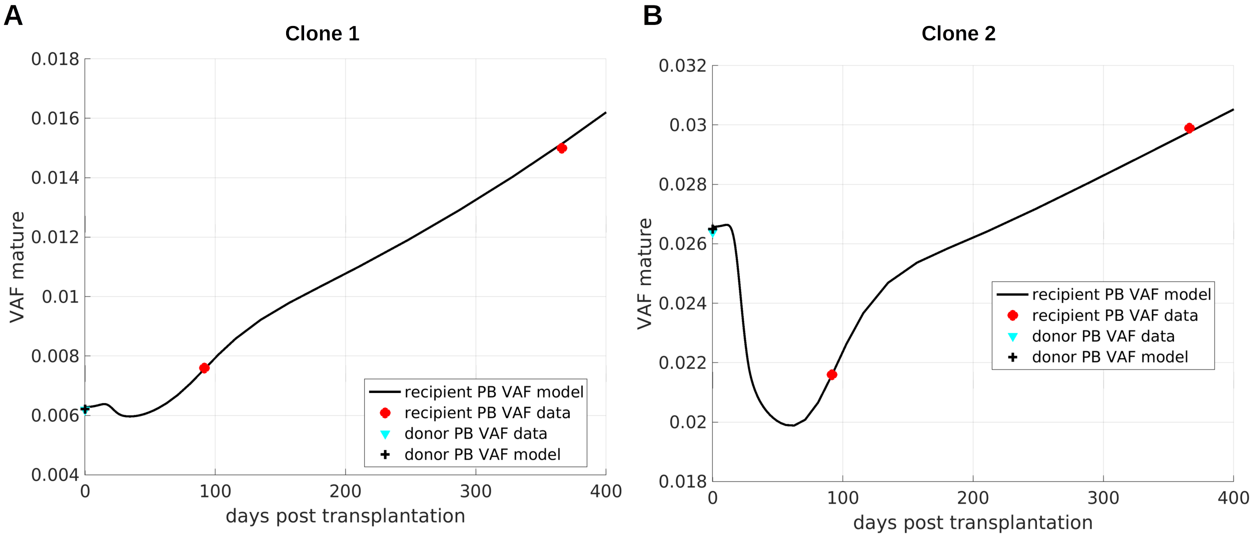

Supplementary Figure 7: **Comparison of the model to a patient harboring two clones.** The data has been taken from the Supplement of [17] (ID: DCH0704). (A) Frameshift mutation of TET2, (B) non-synonymous mutation of DNMT3A.

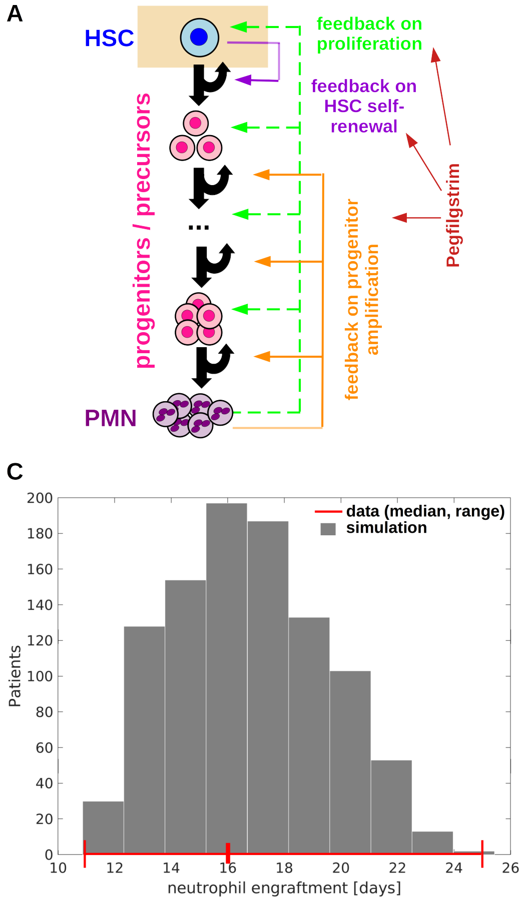

Supplementary Figure 8: **Model of allogeneic hematopoietic stem cell transplantation where HSC self-renewal is regulated by the stem cell niche.** (A) Overview of model structure and feedback regulations. We assume that HSC self-renewal is regulated by the stem cell niche. If there is a shortage of HSCs in the niche, HSC self-renewal probability increases. If there is a shortage of mature cells, HSC and HPC proliferation as well as the number of divisions HPC perform before terminal differentiation increase. Exogenous cytokines such as pegfilgrastim have the same effects as endogenous feedback signals. (B) Example simulation of neutrophil engraftment with and without growth factor support. At time zero $4.7 \cdot 10^6$ CD34+ cells per kg of body weight are transplanted. The time to neutrophil engraftment (time to reach $5 \cdot 10^8$ neutrophils per liter of blood) is in line with observations from clinical trials [40]. (C) Neutrophil engraftment after simulated allogeneic HSCT in a cohort of 1000 virtual patients. Each patient is characterized by an individual set of model parameters which was randomly generated. The number of transplanted CD34+ is $4.7 \cdot 10^6$ per kg of body weigh in analogy to [40]. The red lines indicate the median and the range of the time to neutrophil engraftment observed in the trial from [40]. The deviations between data and simulation are less than one day, which is acceptable since in clinics engraftment is measured in full days. (D) Neutrophil engraftment after simulated allogeneic HSCT with pegfilgrastim support (one dose at day 3). The transplantations are simulated for the same virtual patient cohort as shown in (C). The reduction of the time to neutrophil engraftment by approx. 3 days is in line with clinical observations [20]. Details are provided in Section 1.4.1.

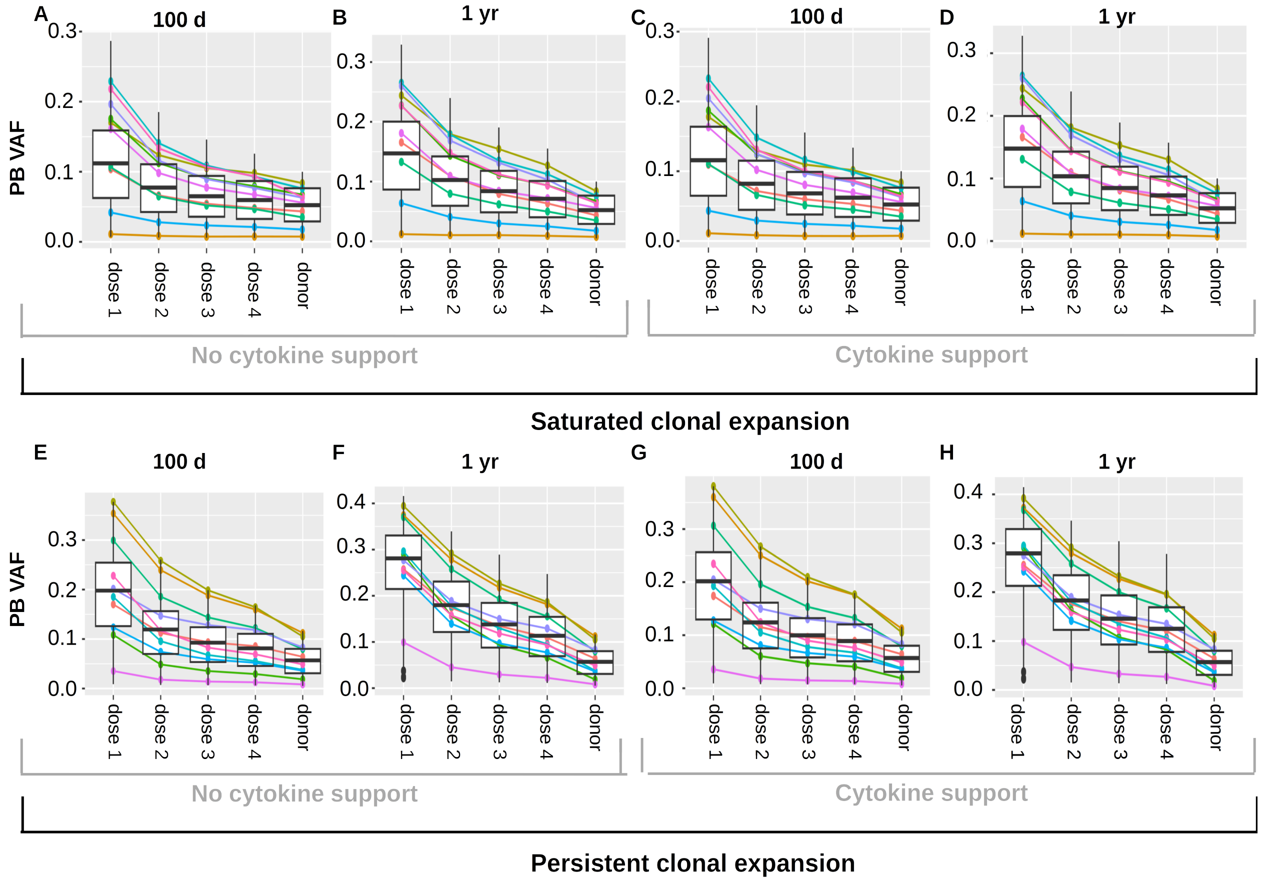

Supplementary Figure 9: **Impact of transplant dose on clonal dynamics for a model where HSC self-renewal is regulated by the niche**. The figure summarizes the results of a virtual clinical trial. (A)-(D) depict a cohort with saturated clonal expansion, (E)-(H) depict a cohort with persistent clonal expansion. Each cohort consists of 1000 virtual patients. The colored lines visualize the results for 10 randomly chosen virtual patients. Donors' PB VAFs are between 0.005 and 0.125. Different cell doses were transplanted and the recipient VAFs in PB were recorded 100 days and one year after transplantation. For the sake of direct comparison all results shown in (A)-(D) were obtained from the same virtual patient cohort. The columns "dose 1", "dose 2", "dose 3", dose 4" show the recipients' VAFs 100 days or 1 year after transplantation of the cell doses 1-4. The column "donor" shows the PB VAF of the donors. Analogously, all results shown in (E)-(H) were obtained from the same virtual patient cohort. (A), (C), (E), (F) relate the PB donor VAF to the PB recipient VAF 100 days after transplantation. (B), (D), (F), (H) relate the PB donor VAF to the PB recipient VAF 1 year after transplantation. In (A), (B), (E), (F) no growth factors were administered after transplantation, in (C), (D), (G), (H) one dose of pegfilgrastim was administered at day 3 after transplantation. Dose 1: $1 \cdot 10^6$ CD34+ cells per kg of body weight, dose 2: $5 \cdot 10^6$ CD34+ cells per kg of body weight, dose 3: $1 \cdot 10^7$ CD34+ cells per kg of body weight, dose 4: $1.5 \cdot 10^7$ CD34+ cells per kg of body weight. The model assumes that HSC self-renewal probability is regulated by the stem cell niche. All other cell properties are subjected to systemic feedbacks. Details are provided in Section 1.4.1.

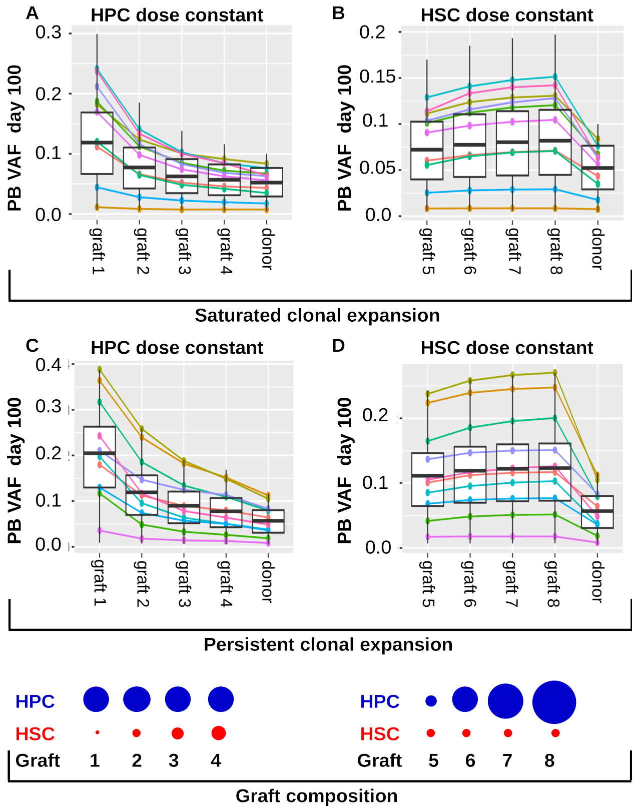

Supplementary Figure 10: **Impact of transplant composition on clonal expansion dynamics for a model where HSC self-renewal is regulated by the niche.** Panels (A)-(B) refer to individuals exhibiting saturated clonal expansion. (A) The 1000 virtual patients from SFig. 9 (A)-(D) are transplanted with grafts containing different HSC numbers. The ratios of transplanted HSC numbers are as follows: graft 1: graft 2: graft 3: graft 4 = 1 : 5 : 10: 15. The number of transplanted non-stem progenitors (HPCs) is the same for all virtual patients. The total transplant dose is $5 \cdot 10^6$ CD34+ cells per kg of body weight. The leftmost 4 columns "graft 1", "graft 2", "graft 3", "graft 4" show the recipients' VAFs 100 days after transplantation of graft 1, graft 2, graft 3, graft 4, respectively. The rightmost column shows the VAF in the donors' peripheral blood. We observe that the recipient's VAFs are higher if the number of transplanted HSCs decreases. The colored lines show the VAFs of 10 randomly selected example virtual patients. (B) The 1000 virtual patients from SFig. 9 (A)-(D) are transplanted with grafts containing different HPC numbers but the same HSC number. The ratios of HPCs are as follows graft 5: graft 6: graft 7: graft 8 = 1 : 5 : 10: 15, corresponding to total transplant doses of $1 \cdot 10^6$, $5 \cdot 10^6$, $1 \cdot 10^7$, $1.5 \cdot 10^7$ CD34+ cells per kg of body weight. The number of HSCs is the same in all grafts and corresponds to the HSC content of a graft of $5 \cdot 10^6$ CD34+ cells per kg of body weight. The leftmost 4 columns "graft 5", "graft 6", "graft 7", "graft 8" show the recipients' VAFs 100 days after transplantation of graft 5, graft 6, graft 7, graft 8, respectively. The rightmost column shows the VAFs in the donors' peripheral blood. We observe that the recipients' VAFs are similar for the different grafts. Panels (C)-(D) refer to individuals exhibiting persistent clonal expansion. The virtual patients are identical to those from SFig. 9 (E)-(H). The grafts in (C) are as in (A), the grafts in (D) are as in (B). The blue and red circles at the bottom of the Figure visualize the transplant composition. We note that graft 2 and graft 6 have have identical compositions. The model assumes that HSC self-renewal probability is regulated by the stem cell niche. All other cell properties are subjected to systemic feedbacks. Details are provided in Section 1.4.1.

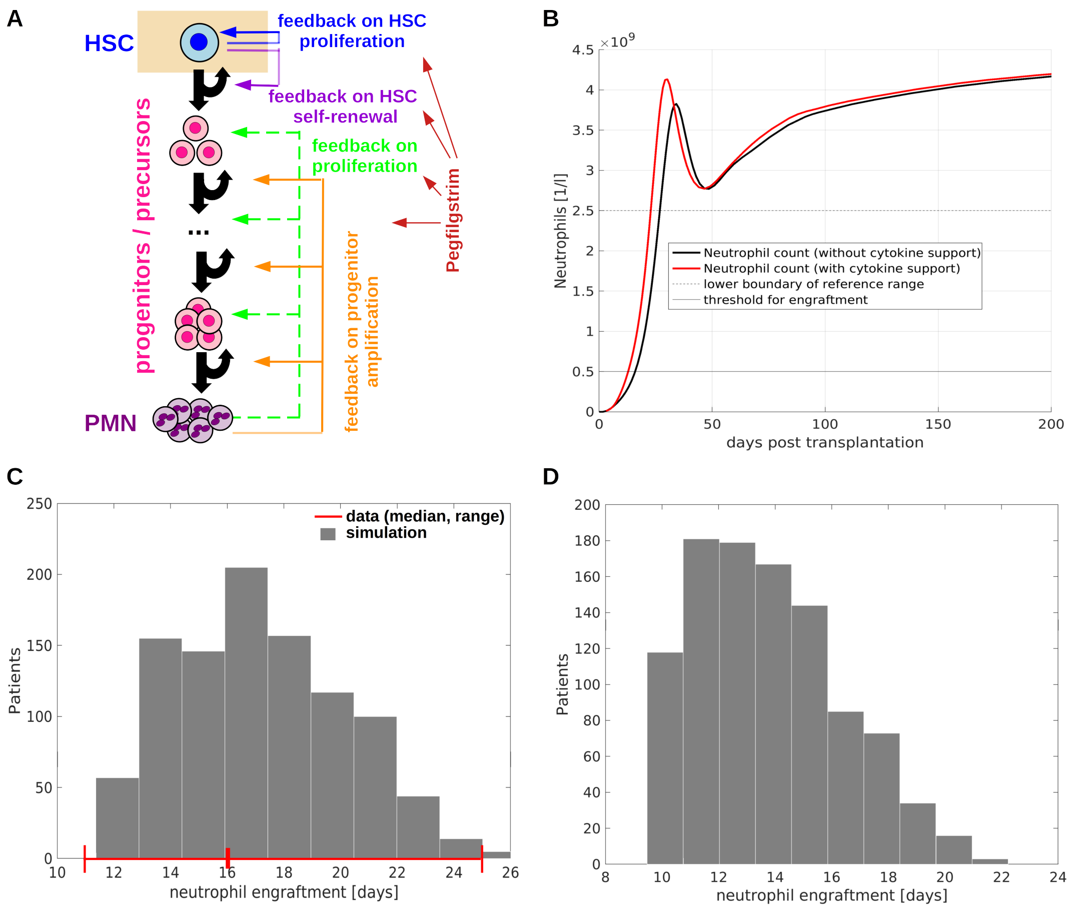

Supplementary Figure 11: **Model of allogeneic hematopoietic stem cell transplantation where HSC self-renewal and proliferation are regulated by the stem cell niche.** (A) Overview of model structure and feedback regulations. We assume that HSC self-renewal and proliferation are regulated by the stem cell niche. If there is a shortage of HSCs in the niche, HSC self-renewal probability and proliferation rate increase. If there is a shortage of mature cells, HPC proliferation as well as the number of divisions HPC perform before terminal differentiation increase. Exogenous cytokines such as pegfilgrastim have the same effects as endogenous feedback signals. (B) Example simulation of neutrophil engraftment with and without growth factor support. At time zero $4.7 \cdot 10^6$ CD34+ cells per kg of body weight are transplanted. The time to neutrophil engraftment (time to reach $5 \cdot 10^8$ neutrophils per liter of blood) is in line with observations from clinical trials [40]. (C) Neutrophil engraftment after simulated allogeneic HSCT in a cohort of 1000 virtual patients. Each patient is characterized by an individual set of model parameters which was randomly generated. The number of transplanted CD34+ is $4.7 \cdot 10^6$ per kg of body weigh in analogy to [40]. The red lines indicate the median and the range of the time to neutrophil engraftment observed in the trial from [40]. The deviations between data and simulation are less than one day, which is acceptable since in clinics engraftment is measured in full days. (D) Neutrophil engraftment after simulated allogeneic HSCT with pegfilgrastim support (one dose at day 3). The transplantations are simulated for the same virtual patient cohort as shown in (C). The reduction of the time to neutrophil engraftment by approx. 3 days is in line with clinical observations [20]. Details are provided in Section 1.4.2.

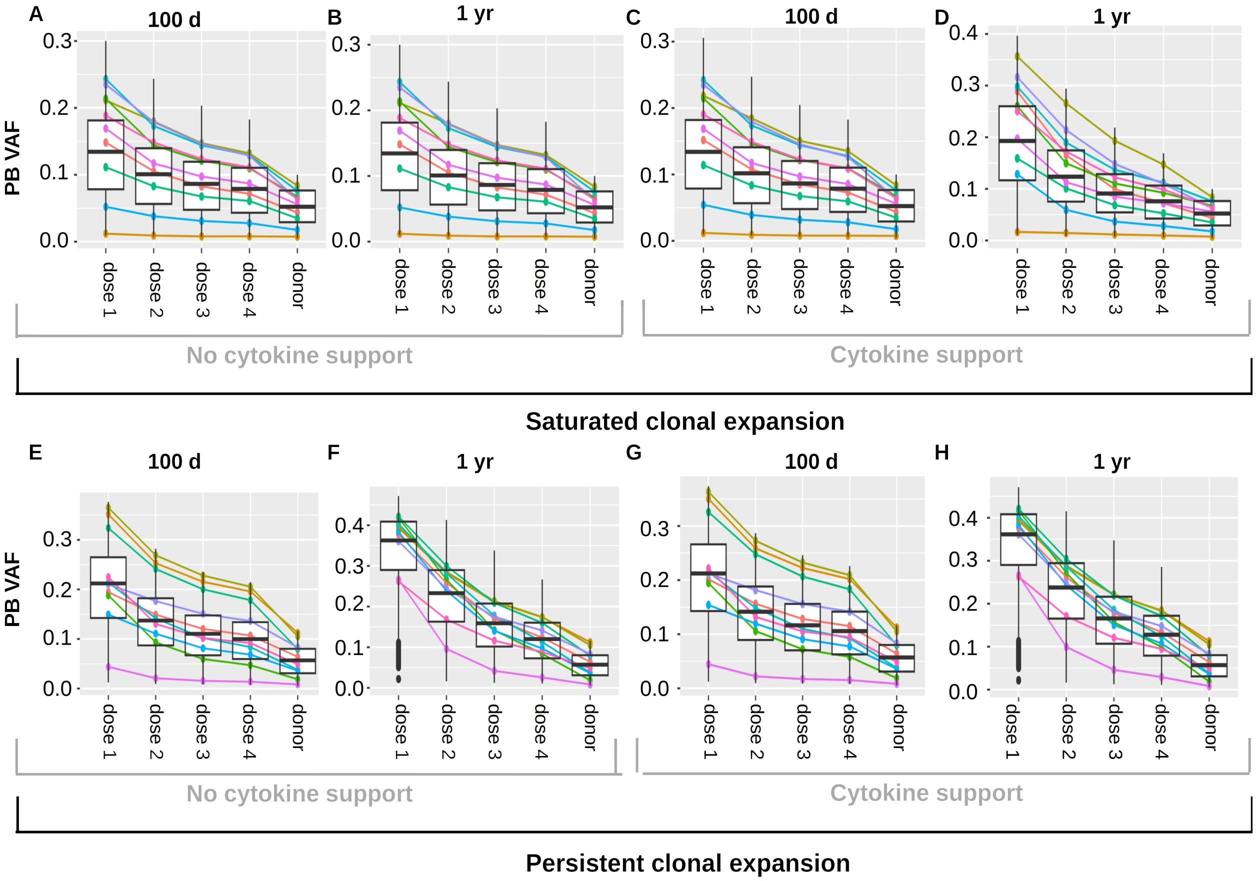

Supplementary Figure 12: **Impact of transplant dose on clonal dynamics for a model where HSC self-renewal and proliferation are regulated by the niche**. The figure summarizes the results of a virtual clinical trial. (A)-(D) depict a cohort with saturated clonal expansion, (E)-(H) depict a cohort with persistent clonal expansion. Each cohort consists of 1000 virtual patients. The colored lines visualize the results for 10 randomly chosen virtual patients. Donors' PB VAFs are between 0.005 and 0.125. Different cell doses were transplanted and the recipient VAFs in PB were recorded 100 days and one year after transplantation. For the sake of direct comparison all results shown in (A)-(D) were obtained from the same virtual patient cohort. The columns "dose 1", "dose 2", "dose 3", dose 4" show the recipients' VAFs 100 days or 1 year after transplantation of the cell doses 1-4. The column "donor" shows the PB VAF of the donors. Analogously, all results shown in (E)-(H) were obtained from the same virtual patient cohort. (A), (C), (E), (F) relate the PB donor VAF to the PB recipient VAF 100 days after transplantation. (B), (D), (F), (H) relate the PB donor VAF to the PB recipient VAF 1 year after transplantation. In (A), (B), (E), (F) no growth factors were administered after transplantation, in (C), (D), (G), (H) one dose of pegfilgrastim was administered at day 3 after transplantation. Dose 1: $1 \cdot 10^6$ CD34+ cells per kg of body weight, dose 2: $5 \cdot 10^6$ CD34+ cells per kg of body weight, dose 3: $1 \cdot 107$ CD34+ cells per kg of body weight, dose 4: $1.5 \cdot 10^7$ CD34+ cells per kg of body weight. The model assumes that HSC self-renewal probability and proliferation rate are regulated by the stem cell niche. All other cell properties are subjected to systemic feedbacks. Details are provided in Section 1.4.2.

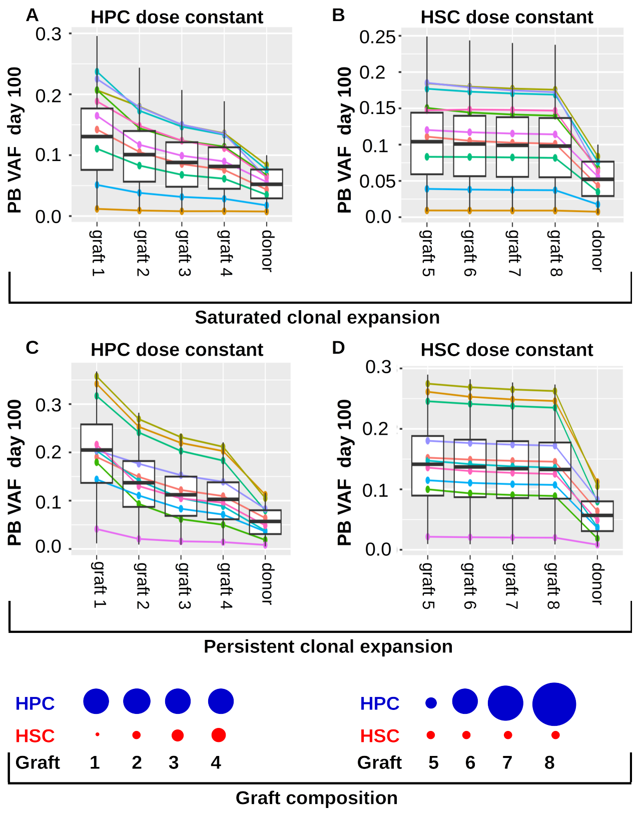

Supplementary Figure 13: **Impact of transplant composition on clonal expansion dynamics for a model where HSC self-renewal and proliferation are regulated by the niche.** Panels (A)-(B) refer to individuals exhibiting saturated clonal expansion. (A) The 1000 virtual patients from SFig. 12 (A)-(D) are transplanted with grafts containing different HSC numbers. The ratios of transplanted HSC numbers are as follows: graft 1: graft 2: graft 3: graft 4 = 1 : 5 : 10: 15. The number of transplanted non-stem progenitors (HPCs) is the same for all virtual patients. The total transplant dose is $5 \cdot 10^6$ CD34+ cells per kg of body weight. The leftmost 4 columns “graft 1”, “graft 2”, “graft 3”, “graft 4” show the recipients’ VAFs 100 days after transplantation of graft 1, graft 2, graft 3, graft 4, respectively. The rightmost column shows the VAF in the donors’ peripheral blood. We observe that the recipient’s VAFs are higher if the number of transplanted HSCs decreases. The colored lines show the VAFs of 10 randomly selected example virtual patients. (B) The 1000 virtual patients from SFig. 12 (A)-(D) are transplanted with grafts containing different HPC numbers but the same HSC number. The ratios of HPCs are as follows graft 5: graft 6: graft 7: graft 8 = 1 : 5 : 10: 15, corresponding to total transplant doses of $1 \cdot 10^6$, $5 \cdot 10^6$, $1 \cdot 10^7$, $1.5 \cdot 10^7$ CD34+ cells per kg of body weight. The number of HSCs is the same in all grafts and corresponds to the HSC content of a graft of $5 \cdot 10^6$ CD34+ cells per kg of body weight. The leftmost 4 columns “graft 5”, “graft 6”, “graft 7”, “graft 8” show the recipients’ VAFs 100 days after transplantation of graft 5, graft 6, graft 7, graft 8, respectively. The rightmost column shows the VAFs in the donors’ peripheral blood. We observe that the recipients’ VAFs are similar for the different grafts. Panels (C)-(D) refer to individuals exhibiting persistent clonal expansion. The virtual patients are identical to those from SFig. 12 (E)-(H). The grafts in (C) are as in (A), the grafts in (D) are as in (B). The blue and red circles at the bottom of the Figure visualize the transplant composition. We note that graft 2 and graft 6 have have identical compositions. The model assumes that HSC self-renewal probability and proliferation rate are regulated by the stem cell niche. All other cell properties are subjected to systemic feedbacks. Details are provided in Section 1.4.2.

References

[1] S. O. Abegunde, R. Buckstein, R. A. Wells, and M. J. Rauh. An inflammatory environment containing tnf alpha favors tet2-mutant clonal hematopoiesis. *Exp Hematol*, 59:60–65, Mar 2018.

[2] European Medicines Agency. Neulasta. *EPAR Product Information*, Annex 1:10, June 2023.

[3] S. Avagyan, J. E. Henninger, W. P. Mannherz, M. Mistry, J. Yoon, S. Yang, M. C. Weber, J. L. Moore, and L. I. Zon. Resistance to inflammation underlies enhanced fitness in clonal hematopoiesis. *Science*, 374(6568):768–772, Nov 2021.

[4] F. Caiado, L. V. Kovtonyuk, N. G. Gonullu, J. Fullin, S. Boettcher, and M. G. Manz. Aging drives Tet2+/- clonal hematopoiesis via IL-1 signaling. *Blood*, 141(8):886–903, Feb 2023.

[5] G. E. CARTWRIGHT, J. W. ATHENS, and M. M. WINTROBE. THE KINETICS OF GRANULOPOIESIS IN NORMAL MAN. *Blood*, 24:780–803, Dec 1964.

[6] S. N. Catlin, L. Busque, R. E. Gale, P. Guttorp, and J. L. Abkowitz. The replication rate of human hematopoietic stem cells in vivo. *Blood*, 117(17):4460–4466, Apr 2011.

[7] I. Cobo, T. Tanaka, C. K. Glass, and C. Yeang. Clonal hematopoiesis driven by DNMT3A and TET2 mutations: role in monocyte and macrophage biology and atherosclerotic cardiovascular disease. *Curr Opin Hematol*, 29(1):1–7, Jan 2022.

[8] C. Colijn and M. C. Mackey. A mathematical model of hematopoiesis: II. Cyclical neutropenia. *J Theor Biol*, 237(2):133–146, Nov 2005.

[9] E. K. Cook, M. Luo, and M. J. Rauh. Clonal hematopoiesis and inflammation: Partners in leukemogenesis and comorbidity. *Exp Hematol*, 83:85–94, Mar 2020.

[10] K. M. Cook, Z. C. Sifri, G. M. Baranski, A. M. Mohr, and D. H. Livingston. The role of plasma granulocyte colony stimulating factor and bone marrow dysfunction after severe trauma. *J Am Coll Surg*, 216(1):57–64, Jan 2013.

[11] GM Cooper and RE Hausman. The eukaryotic cell cycle. In GM Cooper and RE Hausman, editors, *The cell - A moelcular approach , 4th ed.*, chapter 16, pages 650–688. Sinauer Associates, Sunderland, 2007.

[12] M. Craig, A. R. Humphries, and M. C. Mackey. A Mathematical Model of Granulopoiesis Incorporating the Negative Feedback Dynamics and Kinetics of G-CSF/Neutrophil Binding and Internalization. *Bull Math Biol*, 78(12):2304–2357, 12 2016.

[13] M. Craig, A. R. Humphries, F. Nekka, J. Belair, J. Li, and M. C. Mackey. Neutrophil dynamics during concurrent chemotherapy and G-CSF administration: Mathematical modelling guides dose optimisation to minimise neutropenia. *J Theor Biol*, 385:77–89, Nov 2015.

[14] EP. Cronkite. Kinetics of granulocytopoiesis. *Clin Haematol*, 8(2):351–370, 1979.

[15] A. H. Cull, B. Snetsinger, R. Buckstein, R. A. Wells, and M. J. Rauh. Tet2 restrains inflammatory gene expression in macrophages. *Exp Hematol*, 55:56–70, Nov 2017.

[16] Y. Demerdash, B. Kain, M. A. G. Essers, and K. Y. King. Yin and Yang: The dual effects of interferons on hematopoiesis. *Exp Hematol*, 96:1–12, Apr 2021.

[17] C. J. Gibson, H. T. Kim, L. Zhao, H. M. Murdock, B. Hambley, A. Ogata, R. Madero-Marroquin, S. Wang, L. Green, M. Fleharty, T. Dougan, C. A. Cheng, B. Blumenstiel, C. Cibulskis, J. Tsuji, M. Duran, C. D. Gocke, J. H. Antin, S. Nikiforow, A. E. DeZern, Y. B. Chen, V. T. Ho, R. J. Jones, N. J. Lennon, D. R. Walt, J. Ritz, R. J. Soiffer, L. P. Gondek, and R. C. Lindsley. Donor Clonal Hematopoiesis and Recipient Outcomes After Transplantation. *J Clin Oncol*, 40(2):189–201, Jan 2022.

[18] R. Hans, R. R. Sharma, and N. Marwaha. Effect of plateletpheresis on postdonation serum thrombopoietin levels and its correlation with platelet counts in healthy voluntary donors. *Asian J Transfus Sci*, 13(1):10–16, 2019.

[19] M. Hoeglund. Hematopoietic effects and clinical application of granulopoietic growth factors. *Ups J Med Sci*, 101(2):121–148, 1996.

[20] H. J. Khoury, F. R. Loberiza, O. n, A. J. Barrett, B. J. Bolwell, J. Y. Cahn, R. E. Champlin, R. P. Gale, G. A. Hale, A. Urbano-Ispizua, R. Martino, P. L. McCarthy, P. Tiberghien, L. F. Verdonck, and M. M. Horowitz. Impact of posttransplantation G-CSF on outcomes of allogeneic hematopoietic stem cell transplantation. *Blood*, 107(4):1712–1716, Feb 2006.

[21] J. E. Layton, H. Hockman, W. P. Sheridan, and G. Morstyn. Evidence for a novel in vivo control mechanism of granulopoiesis: mature cell-related control of a regulatory growth factor. *Blood*, 74(4):1303–1307, Sep 1989.

[22] H. Lee-Six, N. F. Øbro, M. S. Shepherd, S. Grossmann, K. Dawson, M. Belmonte, R. J. Osborne, B. J. P. Huntly, I. Martincorena, E. Anderson, L. O'Neill, M. R. Stratton, E. Laurenti, A. R. Green, D. G. Kent, and P. J. Campbell. Population dynamics of normal human blood inferred from somatic mutations. *Nature*, 561(7724):473–478, 09 2018.

[23] H. Maeda, Y. Hitomi, R. Hirata, H. Tohyama, J. Suwata, S. Kamata, Y. Fujino, and N. Murata. The effect of phlebotomy on serum erythropoietin levels in normal healthy subjects. *Int J Hematol*, 55(2):111–115, Apr 1992.

[24] A. Marciniak-Czochra, A. Mikelic, and T. Stiehl. Renormalization group second order approximation for singularly perturbed nonlinear ordinary differential equations. *Math. Methods in Applied Sciences*, 41:5691–5710, 2018.

[25] A. Marciniak-Czochra, T. Stiehl, A. D. Ho, W. Jäger, and W. Wagner. Modeling of asymmetric cell division in hematopoietic stem cells–regulation of self-renewal is essential for efficient repopulation. *Stem Cells Dev*, 18(3):377–385, Apr 2009.

[26] S. Pinho and P. S. Frenette. Haematopoietic stem cell activity and interactions with the niche. *Nat Rev Mol Cell Biol*, 20(5):303–320, 05 2019.

[27] M. Remberger, B. nvold, M. Ali, J. Mattsson, T. Egeland, K. U. Lundin, A. Myhre, I. Abrahamsen, D. Heldal, I. Dybedal, G. E. nnfjord, T. Gedde-Dahl, and Y. isand. Cell Dose Matters in Hematopoietic Stem Cell Transplantation with Peripheral Blood Stem Cells from Sibling Donors. *Clin Hematol Int*, 2(2):74–81, Jun 2020.

[28] DH Ryan. Examination of blood cells. In K Kaushansky, MA Lichtman, E Beutler, TJ Kipps, U Seligsohn, and JT Prchal, editors, *Williams Hematology, 8th ed.*, chapter 2, pages 11–24. McGraw-Hill Medical, New York, 2010.

[29] S. Sano, K. Oshima, Y. Wang, Y. Katanasaka, M. Sano, and K. Walsh. CRISPR-Mediated Gene Editing to Assess the Roles of Tet2 and Dnmt3a in Clonal Hematopoiesis and Cardiovascular Disease. *Circ Res*, 123(3):335–341, Jul 2018.

[30] L. G. Schuettpelz, J. N. Borgerding, M. J. Christopher, P. K. Gopalan, M. P. Romine, A. C. Herman, J. R. Woloszynek, A. M. Greenbaum, and D. C. Link. G-CSF regulates hematopoietic stem cell activity, in part, through activation of Toll-like receptor signaling. *Leukemia*, 28(9):1851–1860, Sep 2014.

[31] B. E. Shepherd, P. Guttorp, P. M. Lansdorp, and J. L. Abkowitz. Estimating human hematopoietic stem cell kinetics using granulocyte telomere lengths. *Exp Hematol*, 32(11):1040–1050, Nov 2004.

[32] T. Stiehl, N. Baran, A. D. Ho, and A. Marciniak-Czochra. Cell division patterns in acute myeloid leukemia stem-like cells determine clinical course: a model to predict patient survival. *Cancer Res*, 75(6):940–949, Mar 2015.

[33] T. Stiehl, A. D. Ho, and A. Marciniak-Czochra. Assessing hematopoietic (stem-) cell behavior during regenerative pressure. *Adv Exp Med Biol*, 844:347–367, 2014.

[34] T. Stiehl, A. D. Ho, and A. Marciniak-Czochra. The impact of CD34+ cell dose on engraftment after SCTs: personalized estimates based on mathematical modeling. *Bone Marrow Transplant*, 49(1):30–37, Jan 2014.

[35] T. Stiehl, A. D. Ho, and A. Marciniak-Czochra. Mathematical modeling of the impact of cytokine response of acute myeloid leukemia cells on patient prognosis. *Sci Rep*, 8(1):2809, 02 2018.

[36] T. Stiehl, W. Wang, C. Lutz, and A. Marciniak-Czochra. Mathematical Modeling Provides Evidence for Niche Competition in Human AML and Serves as a Tool to Improve Risk Stratification. *Cancer Res*, 80(18):3983–3992, 09 2020.

[37] H. Tanaka, K. Ishikawa, M. Nishino, T. Shimazu, and T. Yoshioka. Changes in granulocyte colony-stimulating factor concentration in patients with trauma and sepsis. *J Trauma*, 40(5):718–725, May 1996.

[38] I. Thornley, D. R. Sutherland, R. Nayar, L. Sung, M. H. Freedman, and H. A. Messner. Replicative stress after allogeneic bone marrow transplantation: changes in cycling of CD34+CD90+ and CD34+CD90- hematopoietic progenitors. *Blood*, 97(6):1876–1878, Mar 2001.

[39] G. Vanstraelen, P. Frere, M. C. Ngirabacu, E. Willems, G. Fillet, and Y. Beguin. Pegfilgrastim compared with Filgrastim after autologous hematopoietic peripheral blood stem cell transplantation. *Exp Hematol*, 34(3):382–388, Mar 2006.

[40] A. C. Vigorito, W. M. Azevedo, J. F. Marques, A. M. Azevedo, K. A. Eid, F. J. Aranha, I. Lorand-Metze, G. B. Oliveira, M. E. Correa, A. R. Reis, E. C. Miranda, and C. A. de Souza. A randomised, prospective comparison of allogeneic bone marrow and peripheral blood progenitor cell transplantation in the treatment of haematological malignancies. *Bone Marrow Transplant*, 22(12):1145–1151, Dec 1998.

[41] W. Wang, W. Liu, T. Fidler, Y. Wang, Y. Tang, B. Woods, C. Welch, B. Cai, C. Silvestre-Roig, D. Ai, Y. G. Yang, A. Hidalgo, O. Soehnlein, I. Tabas, R. L. Levine, A. R. Tall, and N. Wang. Macrophage Inflammation, Erythrophagocytosis, and Accelerated Atherosclerosis in Jak2 V617F Mice . *Circ Res*, 123(11):e35–e47, Nov 2018.

[42] W. Wang, T. Stiehl, S. Raffel, V. T. Hoang, I. Hoffmann, L. Poisa-Beiro, B. R. Saeed, R. Blume, L. Manta, V. Eckstein, T. Bochtler, P. Wuchter, M. Essers, A. Jauch, A. Trumpp, A. Marciniak-Czochra, A. D. Ho, and C. Lutz. Reduced hematopoietic stem cell frequency predicts outcome in acute myeloid leukemia. *Haematologica*, 102(9):1567–1577, 09 2017.

[43] Y. Wang, S. Sano, Y. Yura, Z. Ke, M. Sano, K. Oshima, H. Ogawa, K. Horitani, K. D. Min, E. Miura-Yura, A. Kour, M. A. Evans, M. A. Zuriaga, K. K. Hirschi, J. J. Fuster, E. M. Pietras, and K. Walsh. Tet2-mediated clonal hematopoiesis in nonconditioned mice accelerates age-associated cardiac dysfunction. *JCI Insight*, 5(6), Mar 2020.

[44] WC. Zamboni. Pharmacokinetics of pegfilgrastim. *Pharmacotherapy*, 23(8 Pt 2):9S–14S, Aug 2003.

[45] Q. Zhang, K. Zhao, Q. Shen, Y. Han, Y. Gu, X. Li, D. Zhao, Y. Liu, C. Wang, X. Zhang, X. Su, J. Liu, W. Ge, R. L. Levine, N. Li, and X. Cao. Tet2 is required to resolve inflammation by recruiting Hdac2 to specifically repress IL-6. *Nature*, 525(7569):389–393, Sep 2015.

[46] C. Zhu and T. Stiehl. Modelling post-chemotherapy stem cell dynamics in the bone marrow niche of AML patients. *Sci Rep*, 14(1):25060, Oct 2024.
